# Genome-Wide Analysis of lncRNA and mRNA Expression During Differentiation of Abdominal Preadipocytes in the Chicken

**DOI:** 10.1534/g3.116.037069

**Published:** 2017-01-20

**Authors:** Tao Zhang, Xiangqian Zhang, Kunpeng Han, Genxi Zhang, Jinyu Wang, Kaizhou Xie, Qian Xue

**Affiliations:** College of Animal Science and Technology, Yangzhou University, Yangzhou, 225009 Jiangsu, China

**Keywords:** Preadipocyte differentiation, RNA-sequencing, Stage-specific module, Chicken, GenPred, Shared data resources, genomic selection

## Abstract

Long noncoding RNAs (lncRNAs) regulate adipogenesis and other processes associated with metabolic tissue development and function. However, little is known about the function and profile of lncRNAs during preadipocyte differentiation in the chicken (*Gallus gallus*). Herein, lncRNA and mRNA expression in preadipocytes at different stages of differentiation were analyzed using RNA sequencing. A total of 1,300,074,528 clean reads and 27,023 novel lncRNAs were obtained from 12 samples. The number of genes (1336 lncRNAs and 1759 mRNAs; 3095 in total) differentially expressed across various stages declined as differentiation progressed. Differentially expressed genes were found to be involved in several pathways related to preadipocyte differentiation that have been extensively studied, including glycerolipid metabolism, and the mammalian target of rapamycin, peroxisome proliferator-activated receptor, and mitogen-activated protein kinase signaling pathways. To our knowledge, some pathways are being reported for the first time, including the propanoate metabolism, fatty acid metabolism, and oxidative phosphorylation pathways. Furthermore, 3095 differentially expressed genes were clustered into eight clusters, and their expression patterns were determined through K-means clustering. Genes involved in the K2 cluster likely play important roles in preadipocyte differentiation. Six stage-specific modules related to A0 (day 0), A2 (day 2), and A6 (day 6) stages were identified, using weighted coexpression network analysis. Nine central, highly connected .genes in stage-specific modules were subsequently identified, including *XLOC_068731*, *XLOC_022661*, *XLOC_045161*, *XLOC_070302*, *CHD6*, *LLGL1*, *NEURL1B*, *KLHL38*, and *ACTR6*. This study provides a valuable resource for further study of chicken lncRNA and facilitates a better understanding of preadipocyte differentiation in the chicken

Abdominal fat is an important carcass trait of broilers. Production performance of broilers has been significantly improved over decades of breeding and selection. However, the overemphasis on selection for rapid growth rate leads to excessive fat accumulation, especially in local Chinese chicken breeds. Excessive fat is often discarded as waste. Furthermore, excess fat deposition results in reduced feed conversion ratio, carcass yield, laying rate, fertility rate, and hatching rate. Lower levels of abdominal fat have therefore become a major breeding goal in broilers.

Adipose tissue is a complex, essential, highly active metabolic and endocrine organ (Kershaw and Flier 2004). The growth of adipose tissue is primarily due to an increase in the number of adipocyte cells (hyperplasia) and enlargement of adipocytes (hypertrophy). Adipocytes are derived from pluripotent mesenchymal stem cells (MSCs) during the process of adipogenesis (Ding *et al.* 2015). The MSCs have the ability to develop into adipoblasts that then develop into preadipocytes, which are capable of storing lipids. Preadipocytes finally differentiate into adipocytes under specific conditions (Leclercq 1984). The number of cells in mature adipose tissue is thought to be indicative of the proliferation of preadipocytes and their subsequent differentiation into mature adipocytes (Matsubara *et al.* 2013).

Adipogenesis is controlled by a complex process that is regulated by various transcriptional events. In mammals, particularly humans and mice, preadipocyte differentiation has been extensively investigated. Previous studies have identified peroxisome proliferator-activated receptor γ (*PPAR*γ) and CCAAT/enhancer-binding protein (*C/EBPs*) as key genes that regulate adipocyte differentiation (MacDougald and Lane 1995; Morrison and Farmer 2000). Matsubara *et al.* (2005) reported that *PPAR*γ is also a key regulator of preadipocyte differentiation. Recent studies in mammals have demonstrated the functions of some novel transcription factors associated with adipogenesis, such as transcriptional factor of zinc finger protein 423 (*Zfp423*) (Gupta *et al.* 2010); Krüppel-like transcription factors (KLFs) (Banerjee *et al.* 2003; Kaczynski *et al.* 2003; Mori *et al.* 2005); and fibroblast growth factor 10 (*FGF10*) (Yamasaki *et al.* 1999; Sakaue *et al.* 2002).

Several genes have been identified as regulators of adipogenesis and preadipocyte differentiation in chickens, including *KLF2* (Zhang *et al.* 2014a), *KLF3* (Zhang *et al.* 2014b), and *FATP1* (Qi *et al.* 2013). Several recent studies have investigated the regulatory mechanisms of chicken adipogenesis using genome-wide analysis of mRNA (Ji *et al.* 2012; Regassa and Kim 2015) and microRNA (Wang *et al.* 2015). However, little is known about the regulatory mechanisms of adipogenesis. Furthermore, the functions of lncRNAs in chicken adipogenesis remain unknown. In the present study, profiles of preadipocyte lncRNA and mRNA were analyzed during differentiation, using RNA sequencing. This study focused on characterization of the features of lncRNA and identification of differentially expressed lncRNAs and mRNAs during different stages of preadipocyte differentiation. The functions of differentially expressed genes (DEGs) were annotated and the pathways involved were enriched. The present study provides a valuable resource for further study of chicken lncRNA and facilitates a better understanding of the biology of preadipocyte differentiation.

## Materials and Methods

### Primary culture of chicken preadipocytes from abdominal adipose tissue

Chicken preadipocytes from abdominal adipose tissue were cultured according to the method described by Shang *et al.* (2014), with some modifications (see Supplemental Material, File S1). Abdominal adipose tissue weighing ∼4 g was collected from three 14-d-old Jinghai Yellow chickens under sterile conditions. Adipose tissue was washed with phosphate-buffered saline supplemented with penicillin (100 units/ml) and streptomycin (100 µg/ml). The washed tissue was cut into 1-mm^3^ sections with a surgical scissors, and digested in 2 mg/ml collagenase type I (Sangon Biotech, Shanghai, China) with shaking for 65 min at 37°. The digested cell suspension was filtered using 200 and 500 mesh screens, and centrifuged at 300 × *g* for 10 min (22°) to separate the stromal vascular fractions from undigested tissue debris and mature adipocytes. Stromal vascular cells were plated on a 60-mm culture plate at a density of 1 × 10^5^ cells/ml, and cultured with Dulbecco’s modified Eagle’s medium/Ham’s nutrient mixture F-12 and basic medium [10% (v/v) fetal bovine serum, 100 units/ml penicillin, and 100 µg/ml streptomycin] in a humidified atmosphere with 5% (v/v) CO_2_ at 37°, until reaching 90% confluence. The cell culture method used in the present study and the methods described by Shang *et al.* (2014) are presented in the supplemental material (see File S1).

### Induction of abdominal preadipocytes

**Figure 1:**
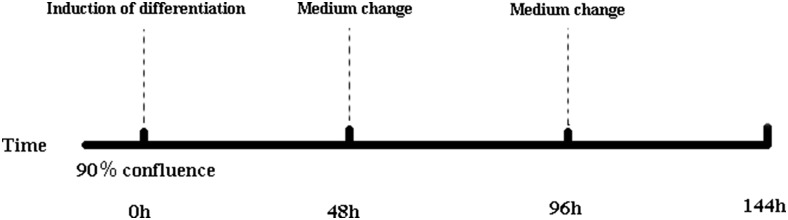
Induction of differentiation in abdominal preadipocytes. The basic medium consisted of Dulbecco’s modified Eagle’s medium/Ham’s nutrient mixture F-12 and 10% fetal bovine serum. The induction differentiation medium consisted of basic medium, insulin, dexamethasone, and 3-isobutyl-1-methylxanthine. The maintenance medium consisted of basic medium and insulin. The induction differentiation medium was replaced with the maintenance medium at 48 hr, whereas the maintenance medium was replaced with basic medium at 96 hr.

After achieving 90% cell confluence, the cells were passaged to 12-well plates and cultured until achieving 90% confluence yet again. The basic medium was then removed and replaced with differentiation medium (0.25 µM dexamethasone, 10 µg/ml insulin, and 0.5 mM 3-isobutyl-1-methylxanthine; all from Takara Bio Inc.) for 48 hr. The differentiation medium was replaced with maintenance medium (10 µg/ml insulin; Takara Bio Inc.) and incubated for 48 hr. The detailed procedure for induction of abdominal preadipocytes is outlined in Figure 1. Cells were collected after being induced for 0, 48, 96, and 144 hr (0, 2, 4, and 6 d). Each interval included three biological replicates (*n* = 3).

### RNA extraction, library construction, and sequencing

A total of 12 cell samples were successfully collected. Total RNA was extracted using TRIzol reagent (Invitrogen, Hong Kong, China). The purity, concentration, and integrity of total RNA were checked using the Nanodrop spectrophotometer, Qubit 2.0 fluorometer, and Agilent 2100 Bioanalyzer, respectively. All RNA samples had an RNA integrity number value >8.0, an optical density 260:280 ratio >1.9, and were selected for library construction and deep sequencing. The rRNA was removed and mRNA was enriched using magnetic beads with oligo (dT) primer, and then randomly fragmented using Fragmentation buffer. The mRNA was used as a template to synthesize the first-strand cDNA, using First Strand Enzyme Mix (Vazyme Biotech Co., Ltd, Nanjing, China). The second-strand cDNA was synthesized using Second Strand Marking Buffer and Second Strand/End Repair Enzyme Mix (Vazyme Biotech Co., Ltd). The products were purified by VAHTS DNA Clean Beads and the end of the double strand was then repaired and A-tailed. An adapter was joined to A-tailed products using ligation mix. Suitably sized fragments were selected using VAHTS DNA Clean Beads to construct the cDNA library by PCR. The RNA sequencing was performed using Illumina XTen (Vazyme Biotech Co., Ltd).

### Quality control

The raw data were subjected to quality control using FastQC (v0.11.4) (http://www.bioinformatics.babraham.ac.uk/projects/fastqc/). The base composition and quality distribution of reads and the GC and AT base content were analyzed, as this could indicate the quality of the overall raw data. Clean data were obtained by removing reads containing adapter contamination, reads containing >10% poly-*N*, and low-quality reads (>50% of bases whose *Q* scores were ≤10%) from the raw data.

### Sequencing data analysis and transcriptome assembly

The clean data were mapped to the chicken reference genome using the Bowtie/TopHat/Cufflinks/Cuffmerge pipeline (Trapnell *et al.* 2012). A filtering step using SAMtools and Linux commands (Li *et al.* 2009) was performed to eliminate those reads showing more than two mismatches to the reference genome and reads with multiple mapping hits. The reference genome files were downloaded directly from the genome website (http://hgdownload.soe.ucsc.edu/goldenPath/galGal4/bigZips/galGal4.fa.gz). The transcripts were assembled based on the genome annotation file (Gallus_gallus.Galgal4.83.gtf) using the Cufflinks v2.2.1 program (Trapnell *et al.* 2012), according to reference annotation based transcript assembly techniques (Roberts *et al.* 2011).

### lncRNA prediction

**Figure 2:**
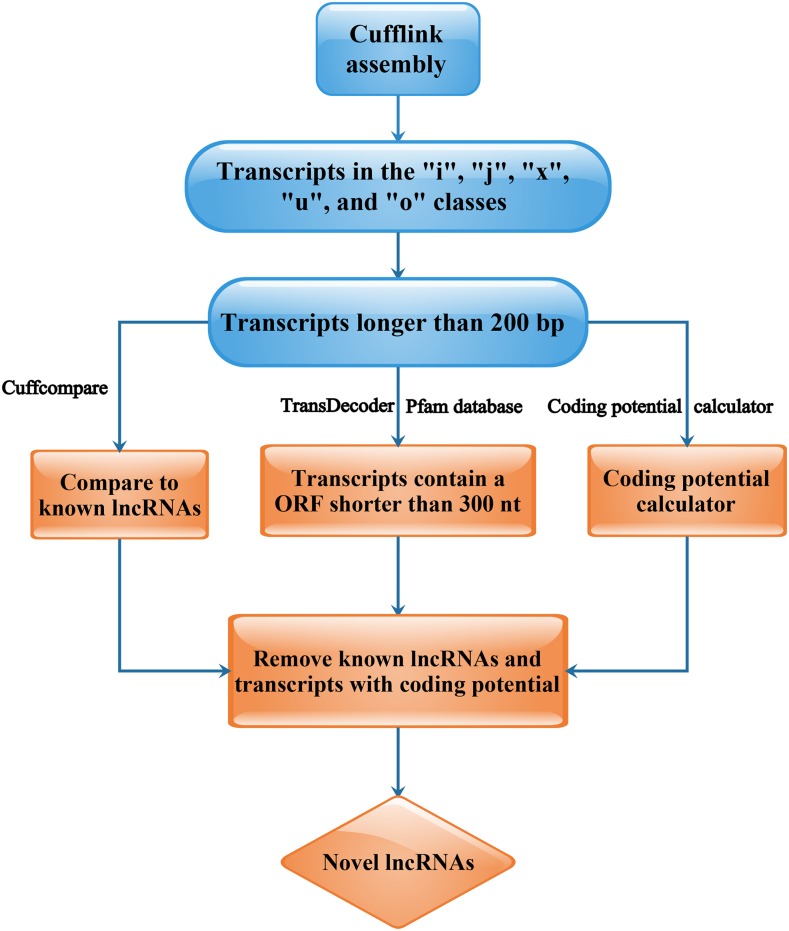
Flow chart of novel lncRNA prediction. For a more detailed flow chart, see File S2. ORF, open reading frame.

Based on the assembly results, transcripts with fragments per kilobase of transcript per million mapped reads = 0 were removed. The filter criteria of lncRNAs were as follows: (1) transcripts in the “i,” “j,” “x,” “u,” and “o” classes were included; (2) transcripts shorter than 200 nt were excluded; (3) transcripts with an open reading frame longer than 300 nt were removed; (4) transcripts containing a specific domain were removed; (5) transcripts that were similar to known proteins were removed; and (6) transcripts that were predicted by the Coding Potential Calculator to encode proteins were removed. The detailed flow of novel lncRNA prediction is presented in Figure 2.

### lncRNA targets prediction and annotation

lncRNA functions by acting on protein-coding genes via the *cis*-acting element and *trans*-acting factor. In the present study, lncRNA targets were predicted based on prediction of *cis* function (Sun *et al.* 2012; Ren *et al.* 2016; Wang *et al.* 2016). The closest coding genes 10 kb upstream and downstream of lncRNAs were screened using the BEDTools v2.25.0 program (Quinlan and Hall 2010). The target genes were then subjected to functional enrichment analysis using the DAVID (v6.7) database (Huang da *et al.* 2009).

### DEG identification and clustering

To identify the genes associated with chicken preadipocyte differentiation, the transcriptome-wide gene expression profiles of RNA-sequencing (RNA-seq) libraries were compared at different differentiation stages. The HTseq v0.6.1 software (Anders *et al.* 2015) was used to calculate reads per kilobase of transcript per million reads of both mRNA and lncRNA in each sample, based on the TopHat BAM files and the reference GTF file. These counts were used in analyses of differential gene expression using the edgeR v3.16.0 software (Robinson *et al.* 2010). For biological replicates, transcripts, or genes with a *P*-value *<*0.05 and fold change ≥2 were identified as DEGs between different groups (Ren *et al.* 2016). The DEGs were subjected to K-means clustering using the Euclidean distance method associated with complete linkage on the BMKCloud platform (https://www.biocloud.net/). All parameters were set as the default.

### Coexpression network analysis

The coexpression network was constructed via the Weighted Gene Co-expression Network Analysis (WGCNA v1.49) package (Langfelder and Horvath 2008) on the BMKCloud platform (https://www.biocloud.net/) with DEGs. All parameters were set as the default. Modules were detected by the dynamic tree cutting method. Stage-specific modules were then identified, based on the correlation between gene significance (GS) and module membership (MM). Modules with significantly correlated GS and MM (*P* < 0.05) were defined as stage-specific. Central and highly connected genes were identified through visualization of the top 200 connections of the top 150 genes, using the Cytoscape v 3.4.0 software platform for each stage-specific module.

### Gene ontology and Kyoto Encyclopedia of Genes and Genomes analysis

Functional annotation enrichment analysis for gene ontology (GO) and Kyoto Encyclopedia of Genes and Genomes (KEGG) were conducted using the DAVID database (Huang da *et al.* 2009). GO terms and pathways with *P*-values <0.05 were considered significantly enriched.

### Validation of gene and lncRNA expression by quantitative real-time PCR

Primers were designed using the NCBI Primer-BLAST tool (http://www.ncbi.nlm.nih.gov/tools/primer-blast/) (Table 1). The first cDNA strand was synthesized using the PrimeScript RT Master Mix (Perfect Real Time) kit (Takara Bio Inc.) according to the user manual. The housekeeping gene was β-actin. Expression of lncRNA and mRNA was quantified using the SYBR Premix Ex Taq II kit (Takara Bio Inc.). The 20 µl PCR reaction mixture contained 10 µl SYBR Premix Ex Taq II (Takara Bio Inc., Dalian, China); 0.4 µl (10 pmol/µl) specific forward primer; 0.4 µl (10 pmol/µl) reverse primer; 0.4 µl ROX reference dye; 2 µl (10 ng/µl) diluted cDNA; and 6.8 µl RNase free water. Cycling parameters were as follows: 95° for 30 sec; followed by 40 cycles at 95° for 5 sec; and 60° for 34 sec. Melting curve analyses were performed following amplification. The ABI 7500 software was used to detect fluorescent signals. Quantification of selected gene expression was performed using the comparative threshold cycle (2^−ΔΔCT^) method. The quantitative real-time PCR (RT-qPCR) results for all genes were statistically tested using the Student’s *t*-test. For detailed bioinformatics analysis, see File S2.

**Table 1:** Primers of genes used in RT-qPCR

Gene ID	Primer Sequence	Accession Number	Product Length (bp)
*ACTR6*	F: CACGTCAGCGTCATTCCCAA	NM_204637.1	99
	R: GCCGGAGGGGTCTTTGATTT		
*CHD6*	F: ACACACAGGGCAATCCTCTC	XM_015296251.1	146
	R: CCTGTTCTTCAAGCGATGCG	
*LLGL1*	F: CTCCAGCAAGGAGGCCAAC	XM_015294418.1	146
	R: AGGTGTCGGCGAAGTAAAGG	
*NEURL1B*	F: ACAGCAGCTTCCAAGACACA	XM_015293830.1	71
	R: GTTGGGCAGGCTGTAGTAGG	
*XLOC_045161*	F: TCAGAGGCCAATACTCCGAAA	TCONS_00063548*^a^*	102
	R: AACACCCTGGAAAGAGCGTG	
*XLOC_070302*	F: ATGTGGGTGAGTTGTGTCGG	TCONS_00097398	87
	R: TGTGATCCAAGGCATCGCTC	
*XLOC_068731*	F: GGCTTATCCCTCAAGCCCC	TCONS_00095558	101
	R: ATGGCCGGAAATGATTCGCA	
*XLOC_022661*	F: CATGCTCTGGTGCTGGAATC	TCONS_00031800	107
	R: CTGCTATCCGGAAGCGTGAA	

a For sequences of lncRNAs see Table S1.

### Data availability

The sequencing data were submitted to the Sequence Read Archive (study accession number SRP093789, run accession number SRR5046458) in NCBI. The details of sequencing run and metadata are presented in Table S15. The assembly files of the whole transcriptome and lncRNA were visualized by submitting to the UCSC genome browser (whole transcriptome: http://genome.ucsc.edu/cgi-bin/hgTracks?hgS_doOtherUser=submit&hgS_otherUserName=Tao%20Zhang&hgS_otherUserSessionName=chicken%20transcriptome; lncRNA: http://genome.ucsc.edu/cgi-bin/hgTracks?hgS_doOtherUser=submit&hgS_otherUserName=Tao%20Zhang&hgS_otherUserSessionName=chicken%20lncRNA). Supplemental materials include 19 files. Table S1 and File S4 contain the sequences, locations, and structures of all lncRNAs. Table S2 contains the target genes 10 kb upstream and downstream of all novel lncRNAs. Table S3 and Table S4 contain the GO and pathway analyses of the target genes, respectively. Table S5 contains all differentially expressed lncRNAs and mRNAs. The common differentially expressed lncRNAs and mRNAs among six comparisons (A0 *vs.* A2; A0 *vs.* A4; A0 *vs.* A6; A2 *vs.* A4; A2 *vs.* A6; and A4 *vs.* A6) are included in Table S6. Table S7 contains genes involved in eight clusters based on the K-means clustering analysis. Table S8 and Table S9 contain the GO and pathway analyses of all DEGs. Table S10 contains genes clustered in the 10 modules. Table S11, Table S12, and Table S13 contain the annotation of genes in A0, A2, and A6 stage-specific modules, respectively. The RNA-seq and RT-qPCR results are included in Table S14. Table S15 contains details of sequencing run and associated metadata in the Sequence Read Archive (SRA). File S1 outlines the method by which chicken preadipocyte were cultured from abdominal adipose tissue in the present study and that of Shang *et al.* (2014). File S2 contains the details of bioinformatics analysis in the present study. File S3 contains the quality control results of sequencing data.

## Results

### Sequencing results and quality control

Raw reads totaling 1,394,219,096 were produced from 12 cDNA libraries. All sequencing data of 12 samples met the requirements for subsequent analysis following quality control (see File S3). Clean reads totaling 1,300,074,582 (195.02 Gb) were obtained. The percentage of clean reads among raw reads in each library ranged from 91.41 to 94.73%. Among the clean reads, the percentage of reads with a Phred quality value >30 ranged from 92.95 to 94.30%. The average GC content of clean reads in 12 samples was 52.51%. Subsequently, clean reads were aligned with the chicken reference genome (http://hgdownload.soe.ucsc.edu/goldenPath/galGal4/bigZips/galGal4.fa.gz). The mapped rate of 12 samples ranged from 79.40 to 84.30%. Among these mapped reads, 66.17–70.07% were mapped to coding DNA sequence regions, 5.34–8.18% to intron regions, 14.49–16.08% to intergenic regions, and 8.83–11.00% to untranslated regions (Table 2). High Pearson correlation coefficients were found among biological replicates of the same differentiation stage, indicating the reproducibility of sample preparation (Figure 5A).

**Table 2:** Statistics of clean reads in chicken preadipocytes

	A0-1	A0-2	A0-3	A2-1	A2-2	A2-3	A4-1	A4-2	A4-3	A6-1	A6-2	A6-3
Total Clean Reads	114,225,220	138,217,716	110,196,390	124,574,594	104,305,988	117,527,324	103,271,936	118,853,588	116,411,784	97,058,458	80,727,160	47,404,424
Base Number (G)	17.13	20.73	16.53	18.69	15.65	17.63	15.49	17.83	17.46	14.56	12.11	11.21
Q30 Reads (%)	94.13	94.28	94.30	93.86	93.35	93.90	92.81	93.04	93.15	93.18	93.20	92.95
Mapped Reads	95,086,176	114,733,972	89,511,292	105,009,893	86,995,436	98,963,734	86,984,909	94,319,305	92,922,064	81,432,206	66,010,294	61,915,627
CDS (%)	68.53	69.93	70.07	69.32	68.73	68.55	66.91	69.80	67.62	66.67	68.50	66.17
Intron (%)	5.86	5.34	5.85	6.36	6.82	7.09	7.32	6.31	7.38	8.18	6.77	8.28
Intergenic (%)	14.60	14.50	14.83	14.49	14.65	14.59	14.93	14.90	15.68	15.11	15.90	16.08
UTR (%)	11.00	10.22	9.25	9.83	9.80	9.77	10.84	9.00	9.33	10.03	8.83	9.47

CDS, coding DNA sequence; UTR, untranslated region.

### Identification of lncRNAs in abdominal preadipocytes

**Figure 3:**
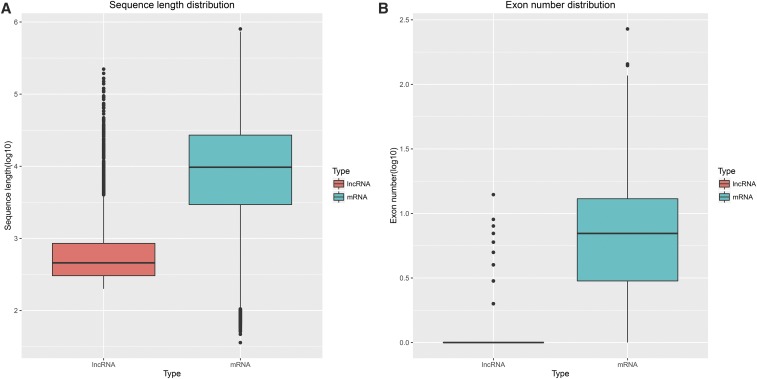
Length and exon number of chicken preadipocyte lncRNAs and mRNAs. (A) Length distribution of mRNAs and lncRNAs. (B) Exon number distribution of mRNAs and lncRNAs.

The lncRNAs identified totaled 27,023 (see Table S1 and File S4). The lengths and exon numbers of lncRNAs were analyzed. lncRNAs were found to be shorter in length and fewer in exon number than protein-coding genes in abdominal preadipocytes (*P* < 0.05)—findings that are in agreement with those of previous studies (Trapnell *et al.* 2010; Wang *et al.* 2016) (Figure 3).

### Functional prediction of lncRNAs in preadipocyte samples

Coding genes 10 kb upstream and downstream of lncRNAs were selected as the *cis* target genes. A total of 4915 target genes were identified (Table S2). To predict the function of lncRNAs in chicken preadipocytes, GO and KEGG analyses were performed using the *cis* target genes (Table S3 and Table S4). A total of 1746, 1544, and 2174 genes were assigned to biological process, cellular component, and molecular function GO categories, respectively. In the biological process category, 27 terms including cellular process, system development, and anatomical structure development were significantly enriched. In the cellular component category, the top three terms were intracellular, intracellular membrane-bounded organelle, and membrane-bounded organelle; whereas in the molecular function category, protein binding and binding protein serine/threonine kinase activity were the most abundant terms. The KEGG enrichment analysis showed that 971 out of 4915 genes were significantly enriched in nine pathways, including the Wnt signaling pathway, mitogen-activated protein kinase (MAPK) signaling pathway, and vascular smooth muscle contraction pathway.

### Differential expression of lncRNAs and mRNAs during abdominal preadipocyte differentiation

**Figure 4:**
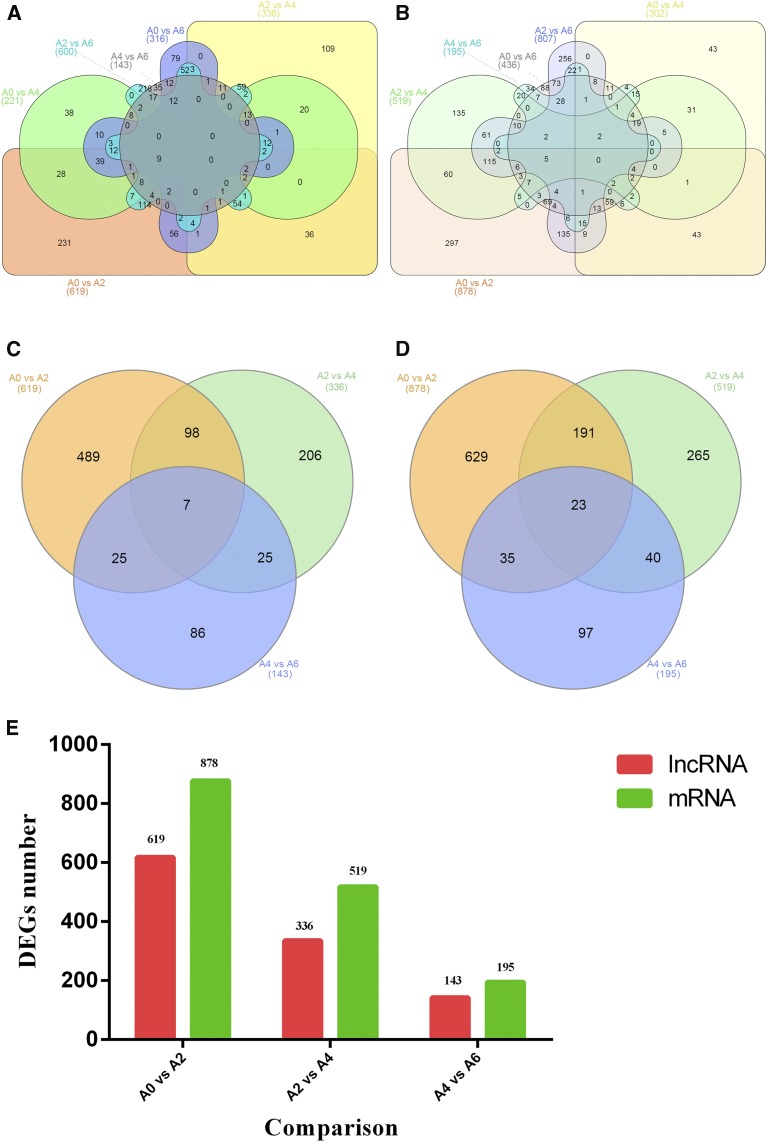
Venn diagram of differentially expressed genes (DEGs) at different stages. (A) Venn diagram of six comparisons of common lncRNAs (A0 *vs.* A2; A0 *vs.* A4; A0 *vs.* A6; A2 *vs.* A4; A2 *vs.* A6; and A4 *vs.* A6). (B) Venn diagram of six comparisons of common mRNAs (A0 *vs.* A2; A0 *vs.* A4; A0 *vs.* A6; A2 *vs.* A4; A2 *vs.* A6; and A4 *vs.* A6). (C) Venn diagram of three comparisons of common lncRNAs (A0 *vs.* A2; A2 *vs.* A4; and A4 *vs.* A6). (D) Venn diagram of three comparisons of common mRNAs (A0 *vs.* A2; A2 *vs.* A4; and A4 *vs.* A6). (E) Histogram of three comparisons of the number of DEGs.

A total of 1336 differentially expressed lncRNAs and 1759 differentially expressed mRNAs were obtained by pairwise comparison (A0 *vs.* A2; A0 *vs.* A4; A0 *vs.* A6; A2 *vs.* A4; A2 *vs.* A6; and A4 *vs.* A6) among the samples collected from preadipocytes on days 0, 2, 4, and 6 of differentiation (Figure 4, A and B, Table S5, and Table S6). No common genes were identified among six comparisons. A total of 936 differentially expressed lncRNAs and 1280 differentially expressed mRNAs were obtained by pairwise comparison (A0 *vs.* A2; A2 *vs.* A4; and A4 *vs.* A6) of the same samples. As shown in Figure 4, C and D and Table S6, 30 DEGs were common among three comparisons (seven lncRNAs and 23 mRNAs). The number of DEGs among three comparisons (A0 *vs.* A2; A2 *vs.* A4; and A4 *vs.* A6) was counted (Figure 4E), and the number of differentially expressed mRNAs and lncRNAs showed a decline as the differentiation of preadipocytes progressed.

**Figure 5:**
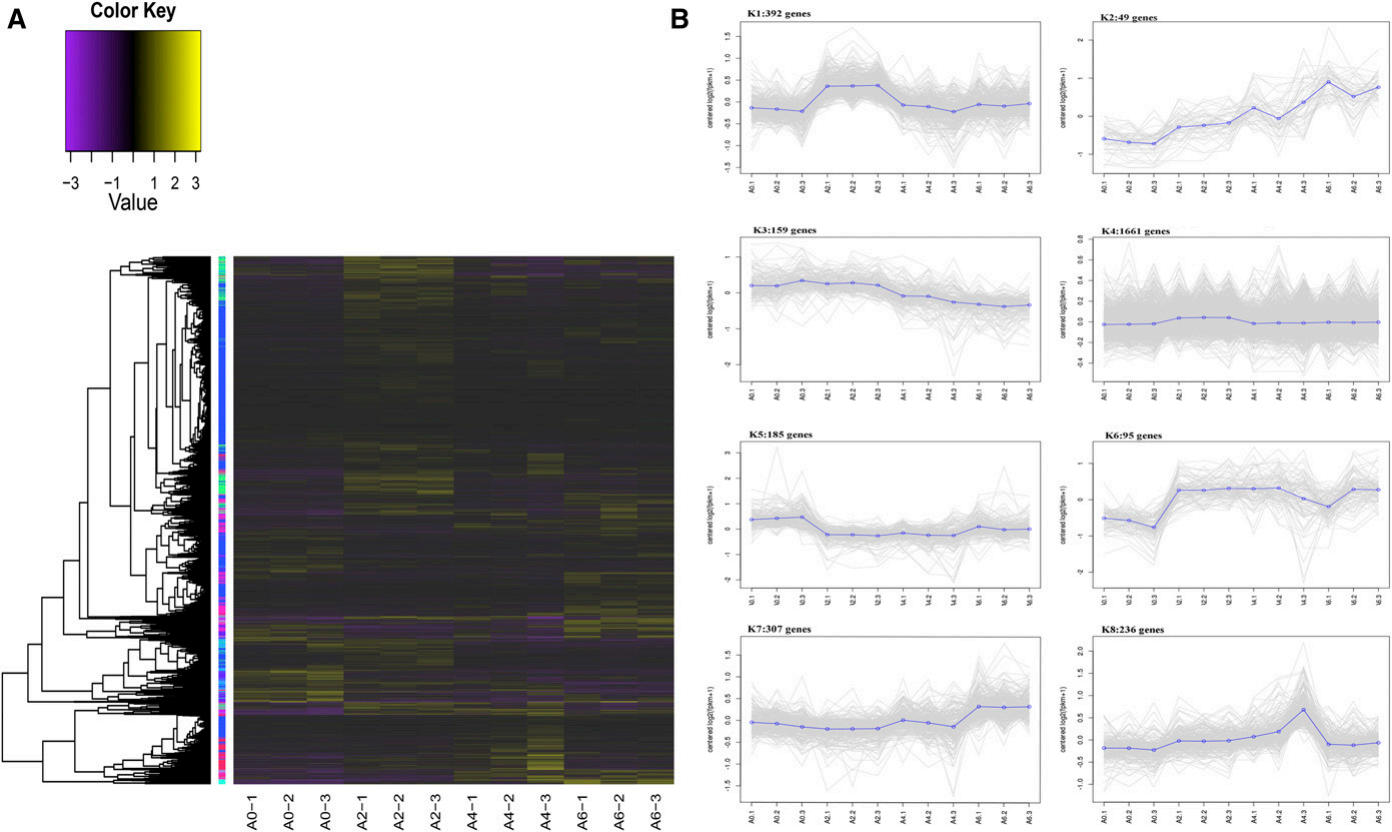
Clustering of all differentially expressed genes (DEGs) (lncRNAs and mRNAs). (A) Heat map shows the K-means clustering of transformed expression values for lncRNAs and mRNAs. Yellow represents higher expression and purple represents lower expression. (B) Expression patterns of genes in eight clusters corresponding to the heat map.

K-means clustering of the 3095 DEGs was conducted using the Euclidean distance method associated with complete linkage (Figure 5). Eight clusters were plotted with expression patterns of the genes involved (Table S7). The K1 cluster included 392 genes that showed upregulation at day 2 of differentiation and then downregulation at days 4 and 6 of differentiation. The 49 genes in the K2 cluster were upregulated across the entire induction process. The expressions of 159 genes in the K3 cluster were slightly downregulated at the A4 and A6 stages compared to the A0 and A2 stages. The K5 cluster included most of the DEGs that were uniformly expressed across four stages. The genes in the K5 and K6 clusters showed opposite patterns of expression. The genes in K5 were significantly downregulated at stages A2, A4, and A6 compared to the first stage; whereas the genes in K6 were significantly upregulated at stages A2, A4, and A6 compared to the first stage. The expression level of genes in K7 remained stable at the first two stages and was then upregulated during the last two stages.

### Functional annotation of DEGs during preadipocyte differentiation

A total of 3095 DEGs were subjected to GO and pathway analysis (Table S8 and Table S9). Terms were considered significantly enriched at *P* < 0.05. The top three enriched terms in the biological process category were cellular metabolic process, cellular process, and electron transport chain. In the molecular function category, the top three enriched terms were transferase activity, protein binding, and nucleoside binding. In the cellular component category, the top three enriched terms were cytoplasmic part, cytoplasm, and extracellular region. Pathway analysis showed that propanoate metabolism, fatty acid metabolism, protein processing in the endoplasmic reticulum, and valine, leucine, and isoleucine degradation pathways were significantly enriched (*P* < 0.05). In addition, dozens of pathways related to adipocyte differentiation were enriched, including those of ATP-binding cassette transporters, fatty acid degradation, and biosynthesis of unsaturated fatty acids.

### Coexpression network and module construction

**Figure 6:**
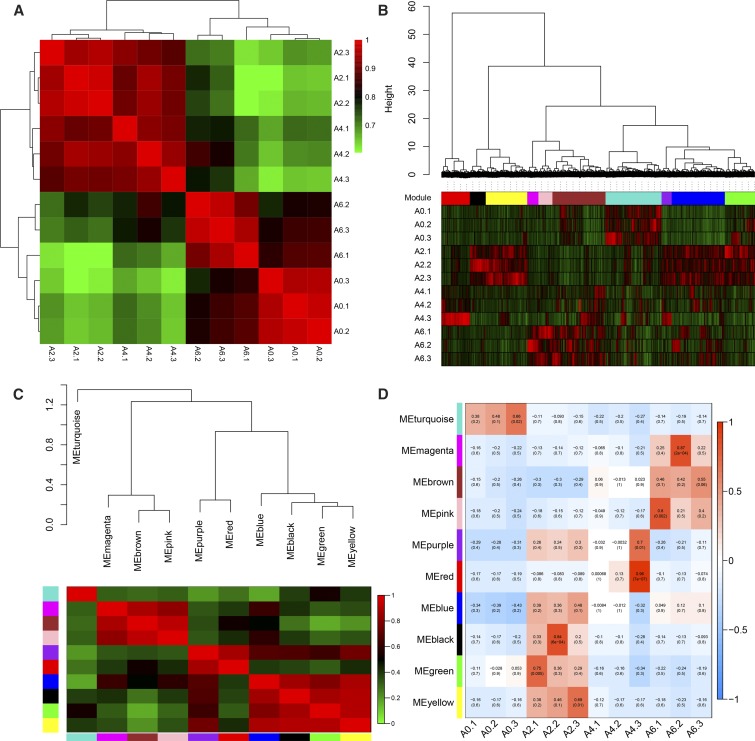
Visualization of construction of the coexpression network. (A) Heatmap of the differentiation of duplicate samples of chicken abdominal preadipocytes. The colors ranging from green to red represent Pearson correlation coefficients ranging from 0.6 to 1, indicating low to high correlations, respectively. All samples at the same differentiation stage are highly correlated, indicating reproducibility of the samples. (B) Hierarchical cluster tree (average linkage, dissTOM) of 3095 genes. The color bands provide a simple visual comparison of module assignments (branch cuttings) based on the dynamic hybrid branch cutting method. (C) Clustering of modules based on eigengenes. The colors ranging from green to red represent Pearson correlation coefficients ranging from 0 to 1, indicating low to high correlations, respectively. (D) Heatmap of correlations between module and differentiation stage. The colors ranging from blue through white to red indicate low through intermediate to high correlations, respectively. ME, the first principal component of the standardized expression profiles of a given module.

Weighted gene coexpression network analysis can be used to find clusters (modules) of highly correlated genes, summarize such clusters using the module eigengene or an intramodular hub gene, and correlate modules with one another and with external sample traits (Langfelder and Horvath 2008). In the present study, 3095 DEGs were subjected to coexpression network analysis, to identify groups of coexpressed genes, referred to as “modules.” Each module was assigned a uniquely colored label beneath the cluster tree (de Jong *et al.* 2012). Ten modules were identified, ranging in size from 99 genes in the purple module to 524 genes in the blue module (Figure 6B and Table S10). Coexpression modules could not exist independently, and formed a meta-network instead. The modules were subjected to clustering analysis based on their eigengenes, to explore and identify the correlations among modules. The results showed that 10 modules were classified into four groups: the first was the turquoise module; the second included the magenta, brown, and pink modules; the third included purple and red modules; and the fourth group included blue, black, green, and yellow modules (Figure 6C). Modules classified within the same group might have the same or similar functions and regulatory mechanisms.

### Identification and visualization of stage-specific modules

**Figure 7:**
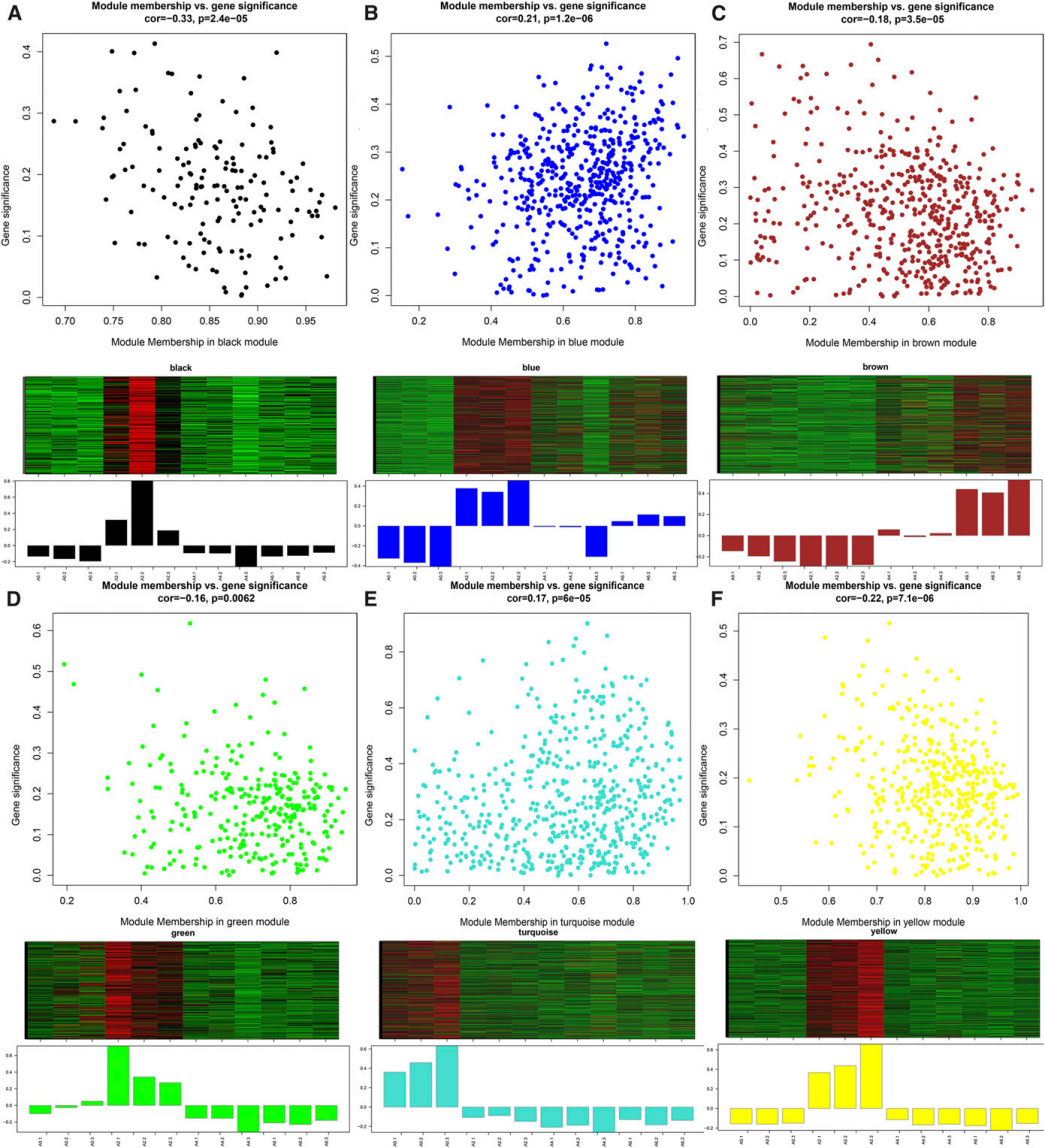
Visualization of gene significance (GS) *vs.* module membership (MM) and gene expression levels of significant modules. Scatterplot represents the GS and MM of each module that shows significant correlation (*P* < 0.05), implying that the module tends to be associated with a specific differentiation stage. Clustering heatmap and bar plot represent gene expression levels of each module. In the heatmaps, the colors range from green to red, indicating low to high expression levels, respectively. A-F represent black, blue, brown, green, turquoise, and yellow modules.

Six stage-specific modules were identified (*P* < 0.05) (Figure 7). The black, blue, green, and yellow modules were positively correlated with the A2 stage (Figure 7, A, B, D, and F); whereas the turquoise and brown modules were positively correlated with stages A0 and A6 (Figure 7, C and E), respectively. These findings indicated that the genes in the modules were predominantly upregulated on days 0, 2, and 6 of differentiation. Furthermore, the blue module was negatively correlated with the A0 stage, suggesting that the genes in this module were predominantly downregulated at day 0 of differentiation. The magenta, pink, and purple modules were also observed but were not significant (*P* > 0.05). The expression pattern of these modules might play an important role in preadipocyte differentiation (Figure 6, B and D). Genes in the magenta and pink modules were expressed at relatively low levels before day 4 of differentiation, after which relatively high expression levels were observed on day 6. Similarly, genes in the purple module showed low levels of expression on day 0 that were increased by day 2, and subsequently declined on days 4 and 6 of differentiation.

### Identification of central and highly connected genes

**Figure 8:**
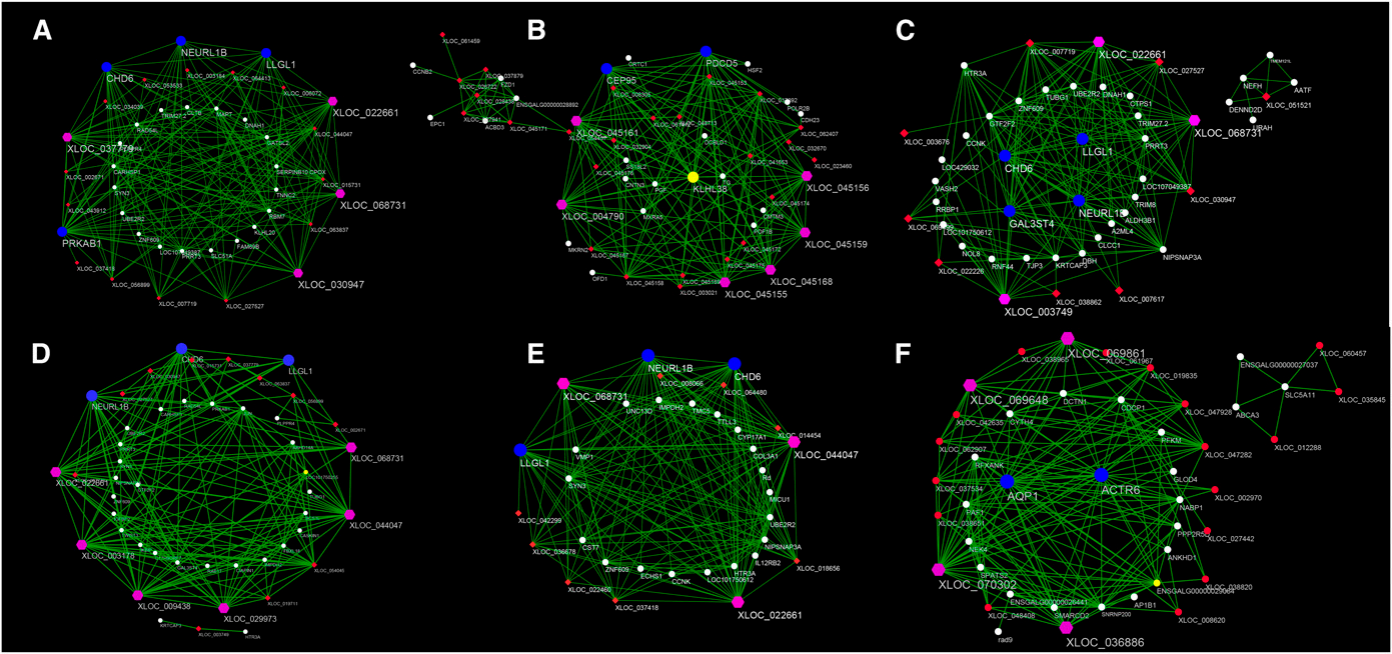
Visualization of connections of genes in various modules. (A–F) Connections of genes in black (A), brown (B), green (C), yellow (D), blue (E), and turquoise (F) modules. Red-colored nodes represent mRNAs. White-colored nodes represent lncRNAs. Pink- and blue-colored nodes indicate common and highly connected lncRNAs and mRNAs, respectively, suggesting their central role in the network.

The top 200 connections of the top 150 highly connected genes for each stage-specific module were analyzed and visualized using the Cytoscape 3.4.0 software to identify genes that were central and highly connected (Figure 8). Five common genes (three lncRNAs and two mRNAs) were identified among four highly correlated modules of black, blue, green, and yellow. This suggests that *XLOC_068731*, *XLOC_022661*, chromodomain helicase DNA binding protein 6 (*CHD6*), lethal giant larvae homolog 1 (*LLGL1*), and neuralized E3 ubiquitin protein ligase 1B (*NEURL1B*) might play important roles in differentiation at the A2 stage. In the brown module, Kelch-like family member 38 (*KLHL38*) and *XLOC_045161* exhibited the most highly connected mRNA and lncRNA, indicating their potential key roles in the regulation of preadipocyte differentiation at the A6 stage. The actin-related protein 6 homolog (*ACTR6*) and *XLOC_070302* shared the highest number of connections, and were observed to be important factors during differentiation at the A0 stage.

### GO and pathway analysis of genes in stage-specific modules

GO analysis revealed the functions of genes in stage-specific modules and pathway analysis revealed essential signaling and metabolic networks in preadipocyte differentiation. The enriched GO terms of biological processes and pathways are presented in Table S11, Table S12, and Table S13. For GO analysis, RNA metabolic process, regulation of localization, and cellular metabolic process were significantly enriched at the A0 stage; whereas generation of precursor metabolites and energy, electron transport chain, and energy derivation by oxidation of organic compounds were highly enriched at the A2 stage; and blastocyst development and cellular process were significantly enriched at the A6 stage (*P* < 0.05). In the pathway analysis, no significant pathway was identified at the A0 and A6 stages. Oocyte meiosis, lysosome, and galactose metabolism were the top three pathways at the A0 stage; whereas biosynthesis of unsaturated fatty acids, apoptosis, and propanoate metabolism were the top three pathways at the A6 stage. Oxidative phosphorylation was significantly enriched (*P* < 0.05) at the A2 stage. Interestingly, three common pathways for oxidative phosphorylation, lysosome, and propanoate metabolism were found at different stages of differentiation.

### Validation of DEGs by RT-qPCR

**Figure 9:**
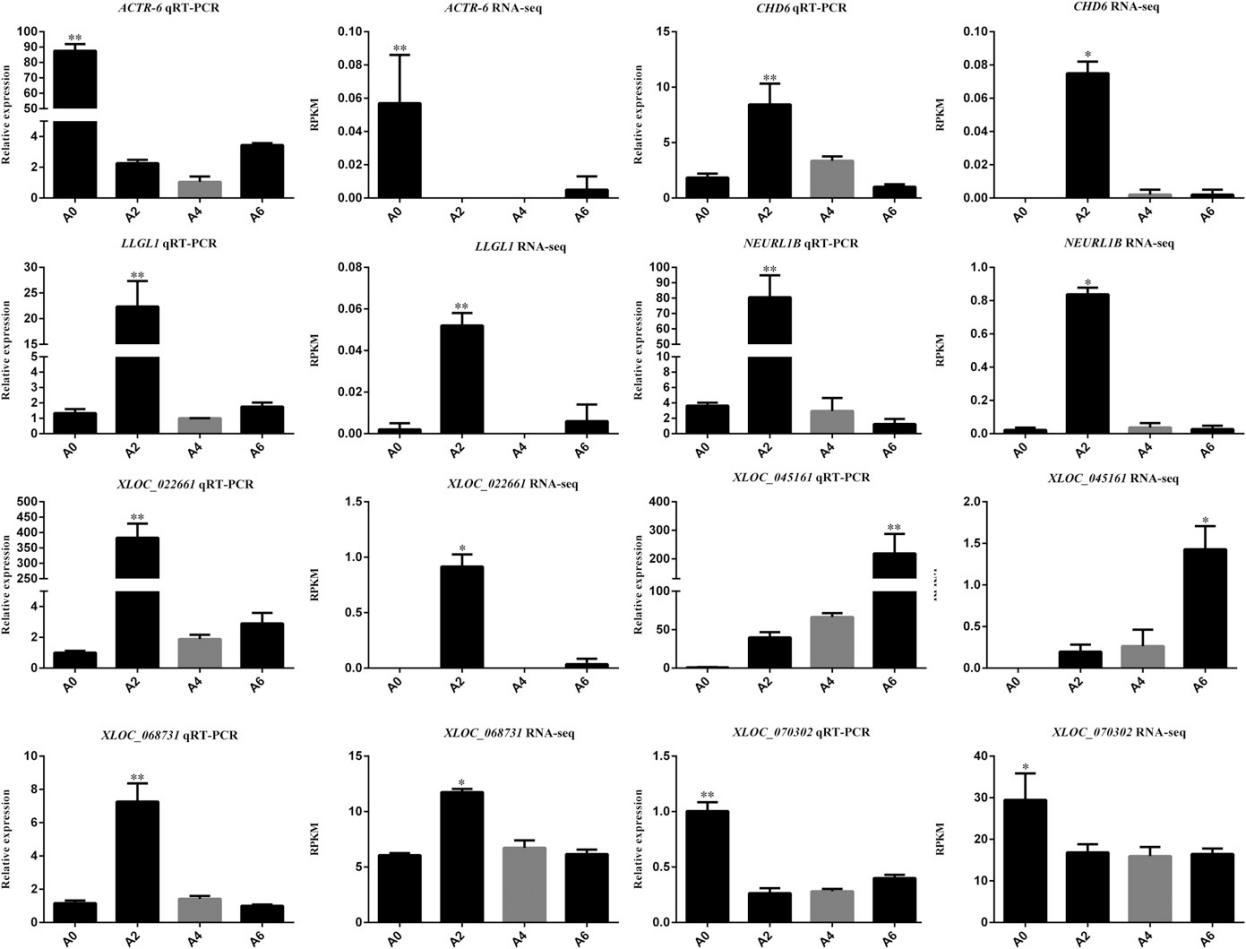
Validation of highly connected genes using RT-qPCR. *P < 0.05, **P < 0.01.

RT-qPCR was conducted to validate the central and highly connected genes *XLOC_045161*, *XLOC_070302*, *XLOC_068731*, *XLOC_022661*, *ACTR6*, *CHD6*, *LLGL1*, and *NEURL1B*. The same 12 cell samples used in RNA-seq were used for RT-qPCR validation. The results showed that the expression patterns of these eight genes were exceptionally consistent with the RNA-seq results (Figure 9 and Table S14).

## Discussion

Over the last 60 yr, the selection of economically important traits has been the main focus of breeding programs and significant genetic improvements have been achieved thus far (Dong *et al.* 2015). Genetic selection for an enhanced growth rate in meat-type chickens (*Gallus gallus domesticus*) is usually accompanied by excessive adiposity that has a negative impact on both feed efficiency and carcass quality (Resnyk *et al.* 2015). Genetic improvement of abdominal fat by standard selection has been minimal for two reasons: (1) the intensity of selection has declined because of the difficulty and cost to measure these phenotypes, and (2) genetic evaluations have been less accurate because they are based on information from relatives only (Demeure *et al.* 2013). Exploration of the molecular regulatory mechanisms of fat deposition in broilers is beneficial for optimal breeding.

Many studies have been conducted to analyze the mechanisms of adipogenesis in the chicken at the genome-wide mRNA expression level (Resnyk *et al.* 2013, 2015; Zhuo *et al.* 2015). The analysis of gene expression merely on mRNA levels has now become more feasible. With the increased feasibility of high-throughput technologies of large-scale expression, RNA-seq has accelerated the discovery and characterization of lncRNAs (Weikard *et al.* 2013). lncRNAs regulate processes associated with metabolic tissue development and function, including adipogenesis, hepatic lipid metabolism, islet function, and energy balance (Alvarez-Dominguez *et al.* 2015; Chen *et al.* 2015; Luan *et al.* 2015; You *et al.* 2015; Zhao and Lin 2015). Despite the fact that many studies indicate the importance of lncRNA in various tissues, little is known about the biological function of lncRNAs in fat deposition of chickens, particularly as it relates to preadipocyte differentiation. In the present study of lncRNAs during preadipocyte differentiation in chickens, 27,023 novel lncRNAs were identified (Table S1 and Table S2). The novel lncRNAs identified in chicken preadipocytes shared many characteristics with those identified in other species, but were relatively shorter in sequence length and lower in exon number.

Most research suggests that lncRNA expression could regulate and is highly correlated with the expression of neighboring mRNAs (Ren *et al.* 2016; Wang *et al.* 2016). To investigate lncRNA function, 4915 target protein-coding genes were identified by searching 10 kb upstream and downstream of the lncRNAs, and GO and pathway analyses were performed (Table S3 and Table S4). In the biological process category, most of the 27 significant terms were associated with the regulation of gene expression, including regulation of cellular process, regulation of biological process, and regulation of cell communication, demonstrating the regulatory role of lncRNAs in the genome. Pathway analysis revealed that nine pathways were significantly enriched, of which four were associated with preadipocyte differentiation including the Wnt, MAPK, and TGF-β signaling pathways. These results suggest that lncRNAs might act in *cis* on neighboring protein-coding genes to regulate abdominal preadipocyte differentiation.

A total of 1336 differentially expressed lncRNAs and 1759 differentially expressed mRNAs were obtained by pairwise comparison (Figure 3, Table S5, and Table S6). Adipocyte differentiation requires cells to process a variety of combinatorial biological groups during the determination of whether or not differentiation proceeds (Gregoire *et al.* 1998). Differentiation itself is characterized by changes in cell morphology and regulated by complex molecular events that are controlled by hormone signaling (Samulin *et al.* 2008). Thus, GO analysis was performed to explore the functions of DEGs during preadipocyte differentiation (Table S8). As expected, the analysis of biological processes showed that a considerable number of DEGs were enriched in processes associated with lipid metabolism and cellular processes. Some general functional terms were also found to be significantly enriched, including response to chemical stimulus, electron transport chain, and metabolic process. These general functional terms might play an essential role in the conversion of preadipocytes to adipocytes. One study has shown that the extracellular environment of intramuscular and subcutaneous preadipocytes might play an important role in adipogenic differentiation (Jiang *et al.* 2013). In the present study, both the extracellular region terms and the cytoplasm terms were significantly enriched, suggesting that both the extracellular and cytoplasmic environment of abdominal preadipocytes play important roles in adipogenic differentiation.

Dynamic changes in gene expression reflect intrinsic mechanisms of an organism’s response to developmental and environmental signals. Genes with similar expression patterns are often hypothesized to have functional dependence and might be coregulated by common regulatory factors (Ye *et al.* 2015). Genes with known functions in a particular cluster can indicate the potential roles of other genes with unknown functions, thereby facilitating the prediction of gene function. In the present study, 3095 DEGs were successfully assembled into eight clusters (Figure 4). Several important clusters associated with preadipocyte differentiation were found. Genes in the K1 cluster showed significant and specific upregulation at day 2 of differentiation, indicating the importance of their contribution to the initiation of preadipocyte differentiation. Genes in the K6 cluster were upregulated at days 2, 4, and 6 in comparison to day 0 of differentiation, suggesting their roles over the entire process of differentiation. The K7 cluster consisted of 307 genes that were significantly upregulated at day 6 of differentiation, demonstrating that those genes might play important roles in the later stages of differentiation. The K2 cluster included genes that showed a notable overall trend of upregulation, suggesting their key roles over the entire process of differentiation. Members of this cluster, such as *GPR39* and *CHCHD4*, reportedly regulate the differentiation of porcine intramuscular preadipocytes (Dong *et al.* 2016). However, their functions in chicken preadipocytes require experimental verification.

Studies have shown that genes and their protein products carry out cellular processes in the context of functional modules, and are related to each other through a complex network of interactions. Understanding the properties of an individual gene or protein within such networks could prove to be just as important as understanding its function in isolation (Hartwell *et al.* 1999; Barabasi and Oltvai 2004; Carlson *et al.* 2006). Therefore, the primary emphasis of the present study was on construction of the coexpression network and detection of modules related to preadipocyte differentiation. Six stage-specific modules were identified (Figure 7) (Zhang and Horvath 2005), suggesting that these modules included genes that were down-regulated or overexpressed during a particular stage of differentiation, and can be used to represent that corresponding stage (Jiang *et al.* 2014). The black, blue, green, and yellow modules were correlated with day 2 (the A2 stage) of differentiation (Figure 6, A, B, D, and F), whereas the turquoise and brown modules were correlated with days 0 (the A0 stage) and 6 (the A6 stage) of differentiation (Figure 6, C and E). Genes in one module suggested their involvement within a common network of biological processes and functions. Interestingly, *IGFBP2* was found to be a highly connected gene in the yellow module correlated with day 2 of differentiation. This gene shows inhibitory effects on preadipocyte differentiation in mice (Xi *et al.* 2013), as well as adipogenesis and lipogenesis in visceral adipocytes (Yau *et al.* 2015). These findings suggest that the yellow module might have an inhibitory effect on the differentiation of preadipocytes on day 2.

To date, several studies have shown that *PPAR*γ and *C/EBP*α (Yu *et al.* 2014), as well as *FATP1* (Qi *et al.* 2013) and *Klf7* (Zhang *et al.* 2013) regulate the differentiation of preadipocytes in the chicken. However, to our knowledge, investigations into the roles of lncRNAs in preadipocyte differentiation in the chicken have not yet been conducted. The molecular and cellular mechanisms regulating chicken preadipocyte differentiation are still poorly understood. In the present study, several highly connected lncRNAs and mRNAs in six stage-specific modules were identified (Figure 8). These genes might play critical roles in the corresponding stages of preadipocyte differentiation in the chicken. Visualization of each module showed that the number of nodes of lncRNAs were greater than that of mRNAs, demonstrating the regulatory role of lncRNAs. Two lncRNAs (*XLOC_068731* and *XLOC_022661*) and three protein-coding RNAs (*CHD6*, *LLGL1*, and *NEURL1B*) were identified as having an association with day 2 of differentiation. *KLHL38* and *XLOC_045161* were the most highly connected mRNA and lncRNA, indicating their potential key roles in the regulation of preadipocyte differentiation on day 6 of differentiation. *ACTR6* and *XLOC_070302* showed the greatest number of connections that were important factors on day 0 of differentiation. None of the lncRNAs obtained in the present study have been previously identified. The lncRNAs and mRNAs were validated by RT-qPCR and the results were consistent with RNA-seq findings (Figure 9), suggesting the reliability of our findings.

As far as we know, only a small number of the pathways involved in preadipocyte differentiation in the chicken have been validated to date, including the AMPK/ACC2 signaling pathway (Gan *et al.* 2015), the MAPK/ATF-2 and TOR/p70 S6 kinase pathways (Yan *et al.* 2013; Jun *et al.* 2014), and the glycerolipid metabolism pathway (Huang *et al.* 2015). In the present study, many well-known pathways associated with different stages of differentiation were identified, including glycerolipid metabolism, mammalian target of rapamycin (mTOR) signaling, PPAR signaling, and MAPK signaling pathways. These pathways might play essential roles in preadipocyte differentiation. Furthermore, many novel pathways associated with preadipocyte differentiation in the chicken were also identified, including propanoate metabolism, fatty acid metabolism, and oxidative phosphorylation. The enriched KEGG pathways associated with preadipocyte differentiation in the mRNAs of stage-specific modules and potential lncRNA targets greatly expanded our knowledge on the pathways involved in adipogenesis of chickens.

In conclusion, this study represents the first analysis of lncRNA and mRNA expression during differentiation of abdominal preadipocytes in the chicken. Several novel lncRNAs were identified. Structural analysis revealed that lncRNAs in chicken preadipocytes are comparatively shorter in sequence length and lower in exon number. A total 3095 DEGs were identified by pairwise comparison of preadipocytes at different stages of differentiation. Hierarchical clustering plotted these DEGs into eight main clusters, of which the K2 cluster might be the most crucial for preadipocyte differentiation. The DEGs were involved in glycerolipid metabolism, and the mTOR, PPAR, and MAPK signaling pathways, findings that are consistent with previous reported on preadipocyte differentiation in chickens. Six stage-specific modules correlating with days 0, 2, and 6 were detected. Nine highly connected genes in stage-specific modules were identified and validated by RT-qPCR, the results of which were consistent with the RNA-seq results. These findings provide a solid foundation for future studies on the molecular mechanisms underlying preadipocyte differentiation in chickens.

## Supplementary Material

Supplemental material is available online at www.g3journal.org/lookup/suppl/doi:10.1534/g3.116.037069/-/DC1.
