# Supplementary material for: Genome-Wide Analysis of lncRNA and mRNA Expression During Differentiation of Abdominal Preadipocytes in the Chicken
Source: G3 (Bethesda). 2017 Jan 20;7(3):953–66. doi: 10.1534/g3.116.037069 (PMC5345725; doi:10.1534/g3.116.037069)
Supplement: Supplementary file 16 [file 953FileS1.docx]

**Primary culture of chicken preadipocytes from abdominal adipose tissue**

In our study, chicken preadipocytes from abdominal adipose tissue were cultured according to the method described by Shang, with some modifications. Here, we give the detailed procedures of the method we used and the method described by Shang. The differences between two methods are marked in red.

Method reported by Shang: Abdominal adipose tissue was collected from 10-day-old broilers by sterile dissection following rapid decapitation. Adipose tissue was washed by pre-warmed PBS supplemented with penicillin (100 units/ml) and streptomycin (100 μg/ml), cut with surgical scissors, and digested in 2 mg/ml collagenase type I (Invitrogen) with shaking for 65 min at 37 ◦C. After digestion, the cell suspension was filtrated through a 20-μm mesh and centrifuged at 300 g for 10 min at room temperature (22 ◦C), to separate the stromal-vascular fractions from undigested tissue debris and mature adipocytes. Stromal-vascular cells (including preadipocytes) were seeded at a density of 5×10^4^ cells/ml in a basal medium [DMEM/F12 (Dulbecco’s modified Eagle’s medium/Ham’s nutrient mixture F-12), 10% (v/v) FBS, 100 units/ml penicillin and 100 μg/ml streptomycin], and maintained in a humidified atmosphere with 5% (v/v) CO2 at 37 ◦C until reaching confluence.

The method we used: Abdominal adipose tissue weighing approximately 4 gm was collected from three 14-day-old Jinghai yellow chicken under sterile condition. Adipose tissue was washed by PBS supplemented with penicillin (100 units/ml) and streptomycin (100 μg/ml). The washed tissue was cut to 1㎜3 by surgical scissors and then digested using 2 mg/ml collagenase type I (Sangon Biotech, Shanghai, China) with shaking for 65 min at 37 ◦C. The digested cell suspension was filtrated using 200 and 500 mesh screens and centrifuged at 300g for 10min (22 ◦C) to separate the stromal-vascular fractions from undigested tissue debris and mature adipocytes. Stromal-vascular cells were plated to 60 mm culture palte at a density of 1×105 cells/ml and cultured with DMEM/F12 (Dulbecco’s modified Eagle’s medium/Ham’s nutrient mixture F-12) basic medium (10% (v/v) FBS, 100 units/ml penicillin and 100 μg/ml streptomycin) in a humidified atmosphere with 5% (v/v) CO2 at 37 ◦C until reaching 90％confluence. After 90％cell confluence, the cells were passaged to twelve-well plates and cultured until the cells were 90％ confluent again. Then, the basic medium was removed and replaced with differentiation medium (0.25 μM dexamethasone (Takara), 10 μg/ml insulin (Takara) and 0.5 mM IBMX (Takara) for 48 hours. The differentiation medium was replaced with maintenance medium (10 μg/ml insulin (Takara)) and incubated for 48 hours. The detailed procedure for induction of abdominal preadipocytes was described in Figure 1. Cells were collected after induced for 0h, 48h, 96h and 144h (0, 2, 4, and 6 d). Each point included 3 biological replicates (n=3).
