# Supplementary material for: Genome-Wide Analysis of lncRNA and mRNA Expression During Differentiation of Abdominal Preadipocytes in the Chicken
Source: G3 (Bethesda). 2017 Jan 20;7(3):953–66. doi: 10.1534/g3.116.037069 (PMC5345725; doi:10.1534/g3.116.037069)
Supplement: Supplementary file 17 [file 953FileS2.docx]

**Details of the bioinformatics analysis**

**1 Quality control**

The quality control was carried out using the fastQC (v0.11.4) tool, which could be downloaded from the Babraham Bioinformatics Web site. http://www.bioinformatics.babraham.ac.uk/projects/fastqc/. FastQC aims to provide a QC report which can spot problems which originate either in the sequencer or in the starting library material. FastQC can be run in one of two modes. It can either run as a stand alone interactive application for the immediate analysis of small numbers of FastQ files, or it can be run in a non-interactive mode where it would be suitable for integrating into a larger analysis pipeline for the systematic processing of large numbers of files.

In our study, the fastQC was run in non-interactive mode on the HPCC system. The command lines for fastQC are as follows.

***1.1 Getting the data***

Copy your data to the working directory:

cp /mnt/research/common-data/Bio/yourdata

Simply copy the following files over to your working directory. First, a "good" sequence in fastq format:

cp /mnt/research/common-data/Bio/AngusData/good_sequence_short.fastq .

Then a "bad" one:

cp /mnt/research/common-data/Bio/AngusData/bad_sequence_short.fastq .

***1.2 Running FastQC***

To run FastQC on the HPCC in interactive mode, you will need to establish an X-connection over SSH. On workstations using the Linux operating system, simply open a terminal and enter:

ssh -X someUser@hpcc.msu.edu

Once you are connected to Gateway with an X-session, you will need to login to one of the dev-nodes before running FastQC:

ssh dev-amd09

Now, simply load the module file for FastQC (remember to do this on a dev-node):

module load FastQC

Analysis of “good” file:

fastqc ./good_sequence_short.fastqc

This will generate a self-contained directory called "good_sequence_short_fastqc" which contains an HTML formatted report that can be loaded into a browser. If we change into that directory and open the contents of the file " fastqc_report.html " we can see:


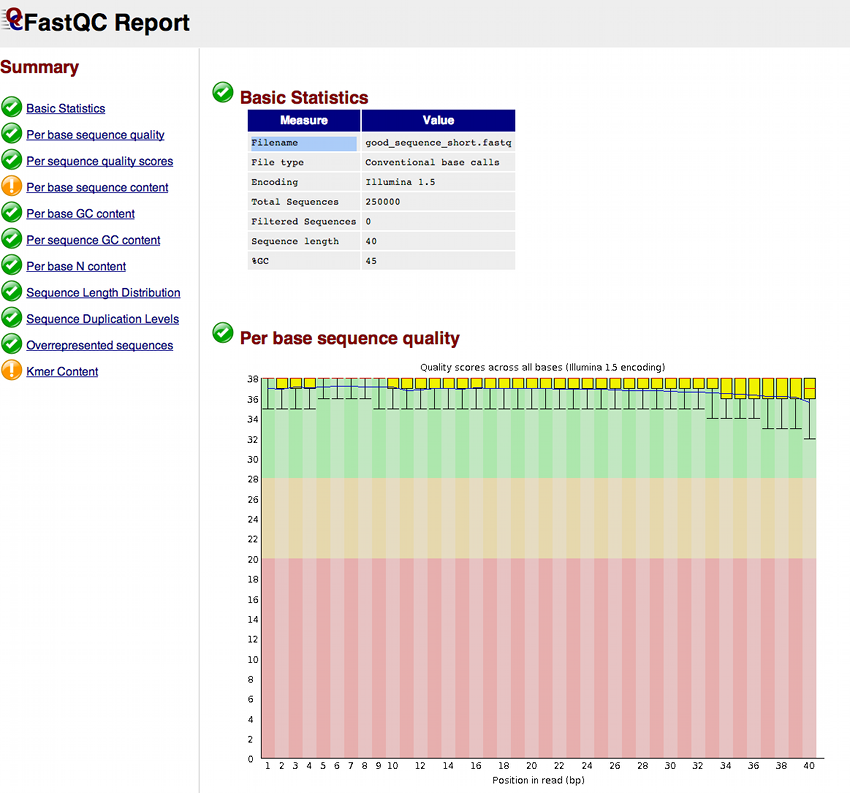


Now we can repeat this procedure using our file of "bad" sequences:

fastqc ./bad_sequence_short.fastqc

We can see:


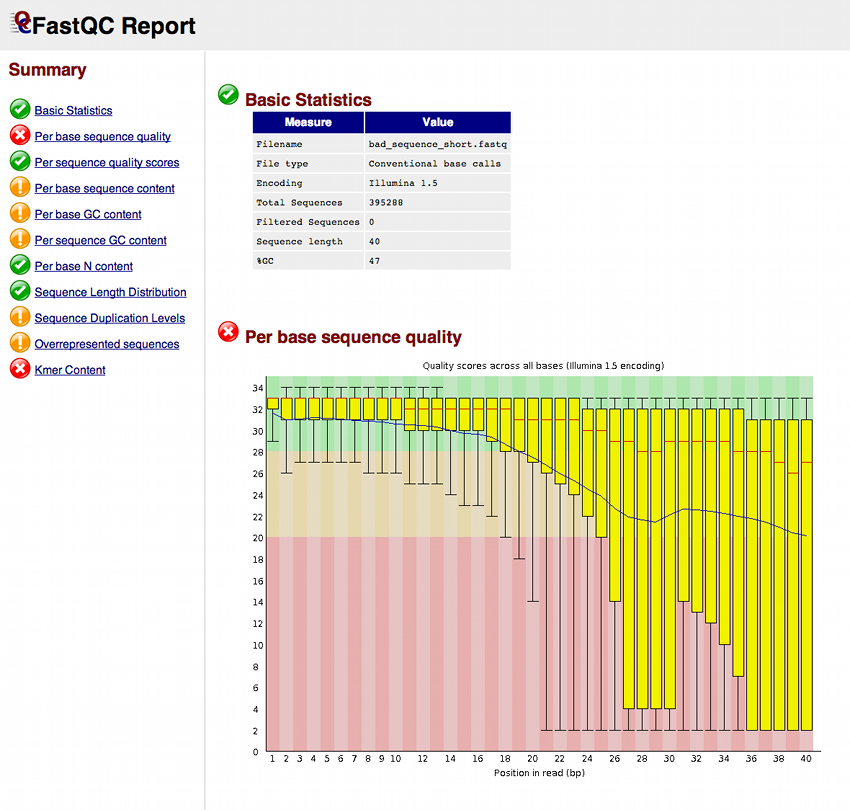


The interpretation of results in the figure above can be seen at Babraham Bioinformatics Web site: http://www.bioinformatics.babraham.ac.uk/projects/fastqc/Help/3%20Analysis%20Modules/.

Then, we obtain clean reads by removing all reads containing an adapter, reads containing poly-N sequences, and low-quality reads from the raw data. At the same time, Q20, Q30, and GC content were calculated for the “clean” dataset. All downstream analyses were based on the clean data. The quality control results of our data can be found in the File S3.

**2 Sequencing data analysis and transcriptome assembly**

The clean data was mapped to the chicken reference genome using the Bowtie/ TopHa/ Cufflinks/ Cuffmerge pipeline ([Trapnell *et al.* 2012](#_ENREF_13)). A filtering step using SAMtools and Linux command ([Li *et al.* 2009](#_ENREF_8)) was performed to eliminate those reads showing more than two mismatches to the reference genomeand reads with multiple mapping hits. The reference genome files were downloaded directly from the genome website (http://hgdownload.soe.ucsc.edu/goldenPath/galGal4/bigZips/galGal4.fa.gz). An index of the reference genome was built using Bowtie v2.1.0.0 (command: --transcriptome-index <dir/prefix>), and paired-end clean reads were aligned to the reference genome by TopHat v2.1.1 using default parameters. The mapped reads of each sample were assembled using Cufflinks v2.2.1 with RABT Assembling strategies ([Roberts *et al.* 2011](#_ENREF_11)). This method employed spliced reads to determine exon connectivity. Cufflinks uses a probabilistic model to simultaneously assemble and quantify the expression levels of a minimal set of isoforms, which provides a maximum likelihood explanation of the expression data in a given locus.

**3 lncRNA prediction**

Novel lncRNA were predicted by a series of strict conditions based on its features.

***3.1 Assembling results filtering***

Based on the assembling results, transcripts with low expression level (FPKM=0) was filtered (code: -F/–min-isoform-fraction <0.0>) to remove the influence of noise on the assembling results.

***3.2 Assembling results merging***

12 assemblies were merged into one master transcriptome using the cuffmerge ([Trapnell *et al.* 2012](#_ENREF_13)) program in cufflink v2.2.1. The merged transcriptome was compared to the known transcripts of chicken from the Ensembl website. Then the transcripts whose class code were “j, i, o, u, x” were selected as candidate lncRNAs.

***3.3 Sequence length filtering***

Long non-coding RNAs (long ncRNAs, lncRNA) are non-protein coding transcripts longer than 200 nucleotides. In order to remove the short miRNA, transcripts longer than 200 nt were selected for the subsequent analysis.

***3.4 Compare to the known lncRNAs***

Chicken lncRNA in the NONCODE v3.0 ([Bu *et al.* 2012](#_ENREF_3)) database were selected as known lncRNAs and aligned with the candidate lncRNAs obtained in step 4.3 using Blastn([Altschul *et al.* 1990](#_ENREF_1)). Transcripts similar to the known lncRNAs were removed.

***3.5 Filtering based on ORF length***

Generally speaking, the open reading frame (ORF) of lncRNA is shorter than that of protein coding RNA. Protein coding RNA usually has a ORF longer than 300nt or 100 amino acid. ORF shorter than 300nt indicates low protein coding possibility of transcript. In our study, the TransDecoder v3.0.0 (http://transdecoder.sourceforge.net/) program was used to find ORFs of the candidate lncRNAs obtained in step 4.3. Additional, the candidate lncRNAs in step 4.3 were aligned with the Pfam 29.0 ([Finn *et al.* 2014](#_ENREF_4)) database to find the protein domains of candidate lncRNAs. Finally, transcripts with ORF shorter than 300nt and containing no protein domains were selected for subsequent analysis.

***3.6 Coding potential analysis***

The CPC-0.9-r2 (Coding Potential Calculator) ([Kong *et al.* 2007](#_ENREF_6)) was used to assess the coding potential of the lncRNAs obtained in step 4.3. The introduction and guide of CPC can be found at the website: http://cpc.cbi.pku.edu.cn/docs/quick_guide.jsp. Transcripts in the noncoding class were selected for the subsequent analysis.

Finally, transcripts passed all the steps above were selected as novel lncRNA in our study.

**4 lncRNA target gene prediction and annotation**

lncRNA functions by acting on protein coding gene via cis-acting element and trans-acting factor. In the present study, lncRNA targets were predicted based on cis function prediction. The closest coding genes to lncRNAs in 10kb of upstream and downstream were screened using Bedtools v2.25.0([Quinlan and Hall 2010](#_ENREF_9)) program. The tutorial of the Bedtools ca be seen at the website: (http://quinlanlab.org/tutorials/bedtools/bedtools.html).

Then the target genes were conducted to functional enrichment analysis using the The Database for Annotation, Visualization and Integrated Discovery (DAVID ) v6.7 ([Huang da *et al.* 2009](#_ENREF_5)). The guide for using DAVID can be seen at: (https://david-d.ncifcrf.gov/content.jsp?file=list_manager.html#upload).

**5 Quantitation of gene expression**

HTseq v0.6.1 ([Anders *et al.* 2015](#_ENREF_2)) was used to calculate reads per kilobase of transcript per million reads (RKPM) of both mRNA and lncRNA in each sample based on the Tophat BAM files and the reference GTF file. These counts were used for gene differential expression analyses using edgeR v3.16.0 ([Robinson *et al.* 2010](#_ENREF_12)). For biological replicates, transcripts or genes with a *p*-value *<* 0.05 and fold change ≥ 2 were defined as differential expressed genes or lncRNAs between two groups([Ren *et al.* 2016](#_ENREF_10)). The tour for using HTseq can be seen at: http://www-huber.embl.de/users/anders/HTSeq/doc/tour.html.

**6 Co-expression network analysis**

The co-expression network was constructed via the Weighted Gene Co-expression Network Analysis (WGCNA v1.49) package ([Langfelder and Horvath 2008](#_ENREF_7)) on the BMKCloud platform (https://www.biocloud.net/) with differentially expressed genes. All parameters were set as the default. Modules were detected by the dynamic tree cutting method. Stage-specific modules were then identified, based on the correlation between gene significance (GS) and module membership (MM). Modules with significantly correlated GS and MM (p < 0.05) were defined as stage-specific. Central and highly connected genes were identified through visualization of the top 200 connections of the top 150 genes, using the Cytoscape (v 3.4.0) software platform for each stage-specific module.

**7 GO and pathway analysis**

Functional annotation enrichment analysis for Gene Ontology (GO) and Kyoto Encyclopedia of Genes and Genomes (KEGG) were conducted using DAVID v6.7 ([Huang da *et al.* 2009](#_ENREF_5)) database. Go terms and pathways with P value less than 0.05 were considered as significantly enriched.

**8 K-means clustering of differentially expressed genes**

The differentially expressed genes were performed to K-means clustering using the expression_cluster_heatmap program at: https://console.biocloud.net/software/softwaretemplate/softwaretemplate?xmlname=expression_cluster_heatmap. This program could be used for free. However, users need to register before using this program.

**9 Primer deisgn**

Primers were designed using the Primer-BLAST in NCBI (http://www.ncbi.nlm.nih.gov/tools/primer-blast/). The product size was set to 70-150 bp. The genome (reference assembly for selected organisms) database and Gallus gallus organism were selected. For the protein coding RNA, primer pair must be separated by at least one intron on the corresponding genomic DNA.

Altschul, S. F., Gish, W., Miller, W., Myers, E. W., and Lipman, D. J., 1990 Basic local alignment search tool. J Mol Biol **215**: 403-10.

Anders, S., Pyl, P. T., and Huber, W., 2015 HTSeq--a Python framework to work with high-throughput sequencing data. Bioinformatics **31**: 166-9.

Bu, D., Yu, K., Sun, S., Xie, C., Skogerbo, G., Miao, R., Xiao, H., Liao, Q., Luo, H., Zhao, G., Zhao, H., Liu, Z., Liu, C., Chen, R., and Zhao, Y., 2012 NONCODE v3.0: integrative annotation of long noncoding RNAs. Nucleic Acids Res **40**: D210-5.

Finn, R. D., Bateman, A., Clements, J., Coggill, P., Eberhardt, R. Y., Eddy, S. R., Heger, A., Hetherington, K., Holm, L., Mistry, J., Sonnhammer, E. L., Tate, J., and Punta, M., 2014 Pfam: the protein families database. Nucleic Acids Res **42**: D222-30.

Huang da, W., Sherman, B. T., and Lempicki, R. A., 2009 Systematic and integrative analysis of large gene lists using DAVID bioinformatics resources. Nat Protoc **4**: 44-57.

Kong, L., Zhang, Y., Ye, Z. Q., Liu, X. Q., Zhao, S. Q., Wei, L., and Gao, G., 2007 CPC: assess the protein-coding potential of transcripts using sequence features and support vector machine. Nucleic Acids Res **35**: W345-9.

Langfelder, P., and Horvath, S., 2008 WGCNA: an R package for weighted correlation network analysis. BMC Bioinformatics **9**: 559.

Li, H., Handsaker, B., Wysoker, A., Fennell, T., Ruan, J., Homer, N., Marth, G., Abecasis, G., and Durbin, R., 2009 The Sequence Alignment/Map format and SAMtools. Bioinformatics **25**: 2078-9.

Quinlan, A. R., and Hall, I. M., 2010 BEDTools: a flexible suite of utilities for comparing genomic features. Bioinformatics **26**: 841-2.

Ren, H., Wang, G., Chen, L., Jiang, J., Liu, L., Li, N., Zhao, J., Sun, X., and Zhou, P., 2016 Genome-wide analysis of long non-coding RNAs at early stage of skin pigmentation in goats (Capra hircus). BMC Genomics **17**: 67.

Roberts, A., Pimentel, H., Trapnell, C., and Pachter, L., 2011 Identification of novel transcripts in annotated genomes using RNA-Seq. Bioinformatics **27**: 2325-9.

Robinson, M. D., McCarthy, D. J., and Smyth, G. K., 2010 edgeR: a Bioconductor package for differential expression analysis of digital gene expression data. Bioinformatics **26**: 139-40.

Trapnell, C., Roberts, A., Goff, L., Pertea, G., Kim, D., Kelley, D. R., Pimentel, H., Salzberg, S. L., Rinn, J. L., and Pachter, L., 2012 Differential gene and transcript expression analysis of RNA-seq experiments with TopHat and Cufflinks. Nat Protoc **7**: 562-78.
