# Supplementary figures and images for: Genome-Wide Analysis of lncRNA and mRNA Expression During Differentiation of Abdominal Preadipocytes in the Chicken
Source: G3 (Bethesda). 2017 Jan 20;7(3):953–66. doi: 10.1534/g3.116.037069 (PMC5345725; doi:10.1534/g3.116.037069)

## Classification of Raw Reads(A0-1)

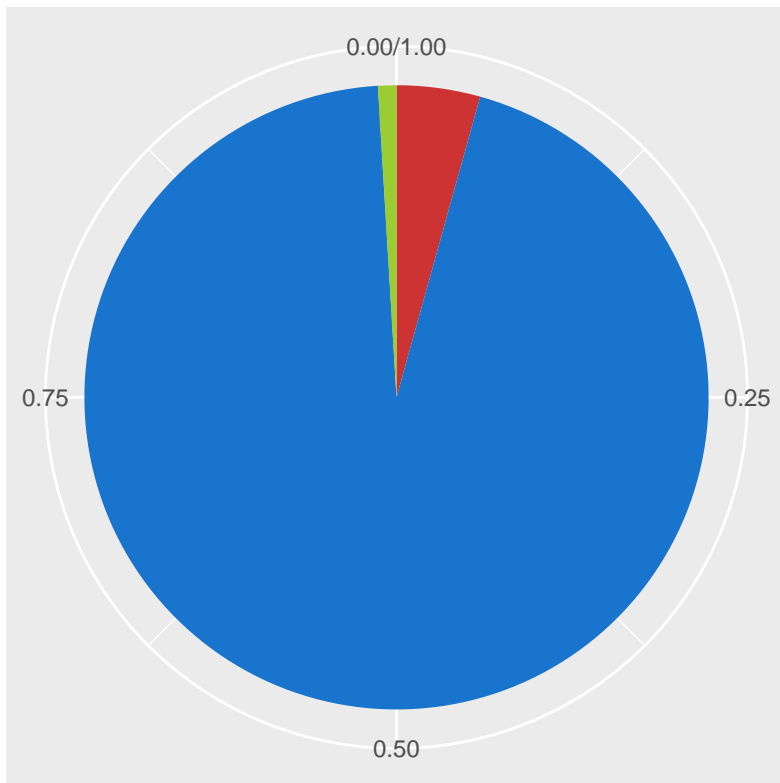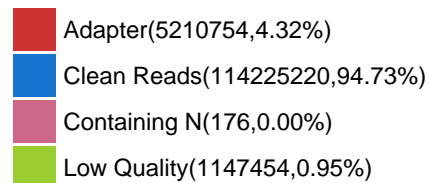

Supplement: Supplementary file 18 [file 953FileS3.zip › QC/A0-1.qc.pdf]

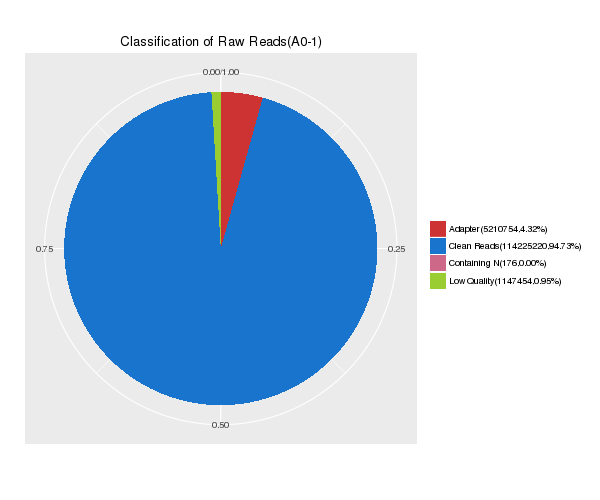

Supplement: Supplementary file 18 [file 953FileS3.zip › QC/A0-1.qc.png]

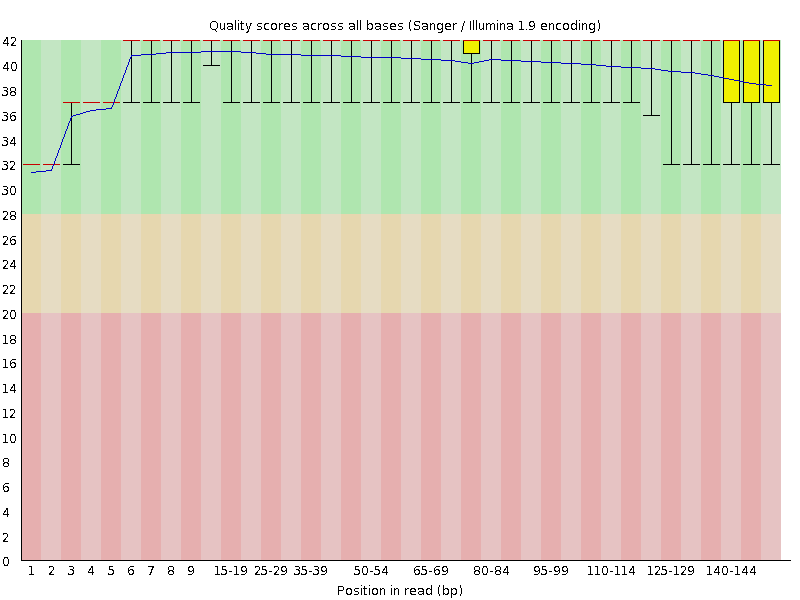

Supplement: Supplementary file 18 [file 953FileS3.zip › QC/A0-1_1.per_base_quality.png]

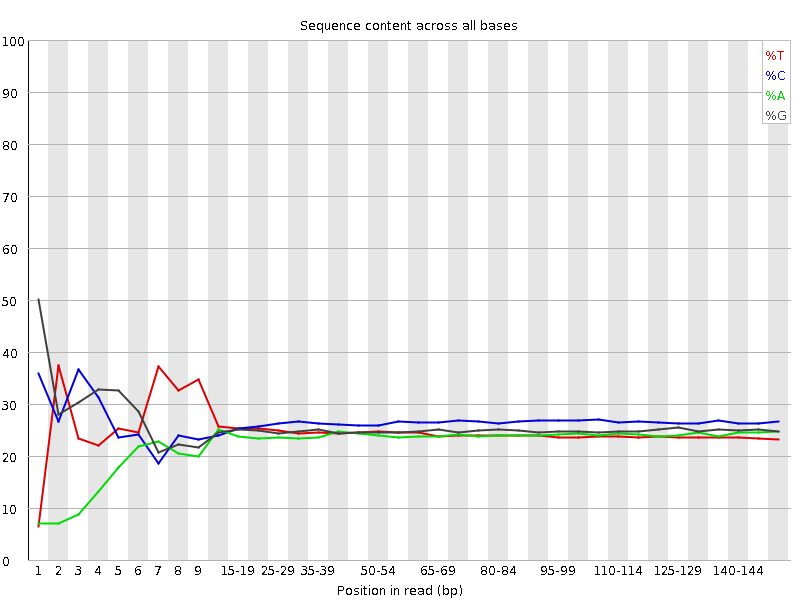

Supplement: Supplementary file 18 [file 953FileS3.zip › QC/A0-1_1.per_base_sequence_content.png]

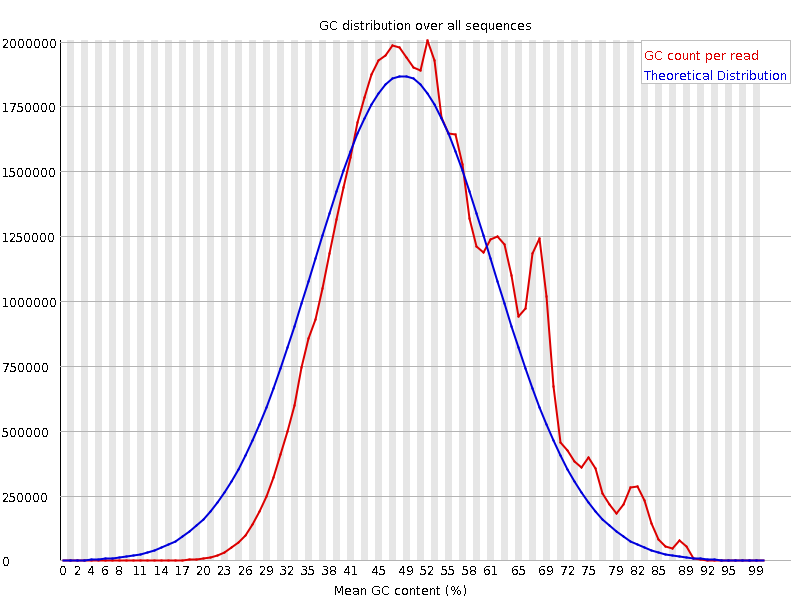

Supplement: Supplementary file 18 [file 953FileS3.zip › QC/A0-1_1.per_sequence_gc_content.png]

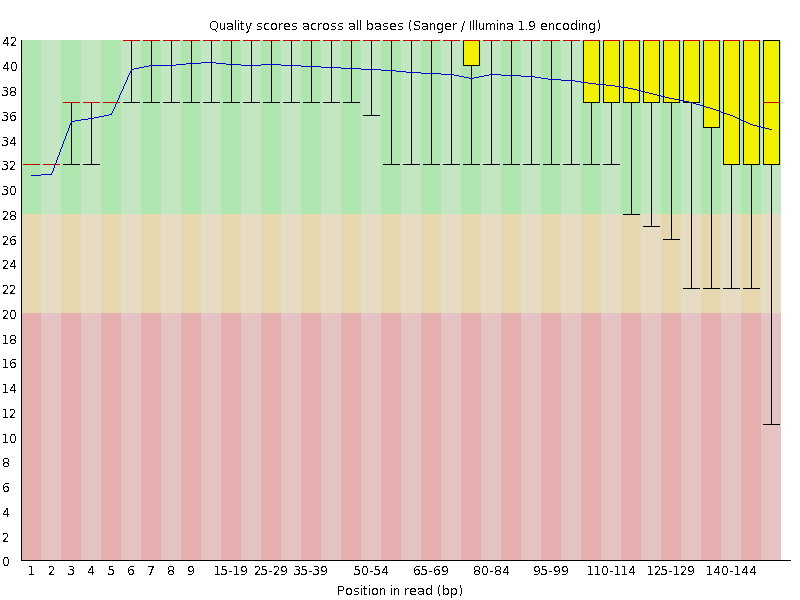

Supplement: Supplementary file 18 [file 953FileS3.zip › QC/A0-1_2.per_base_quality.png]

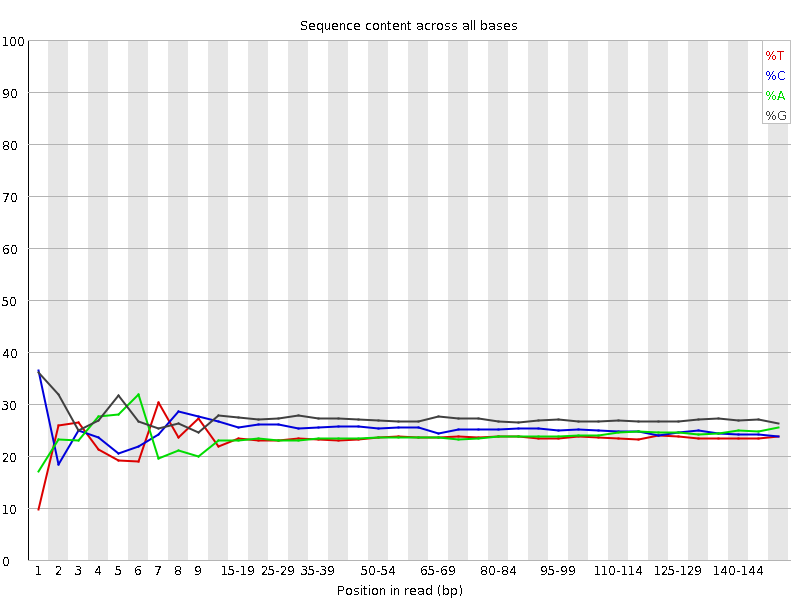

Supplement: Supplementary file 18 [file 953FileS3.zip › QC/A0-1_2.per_base_sequence_content.png]

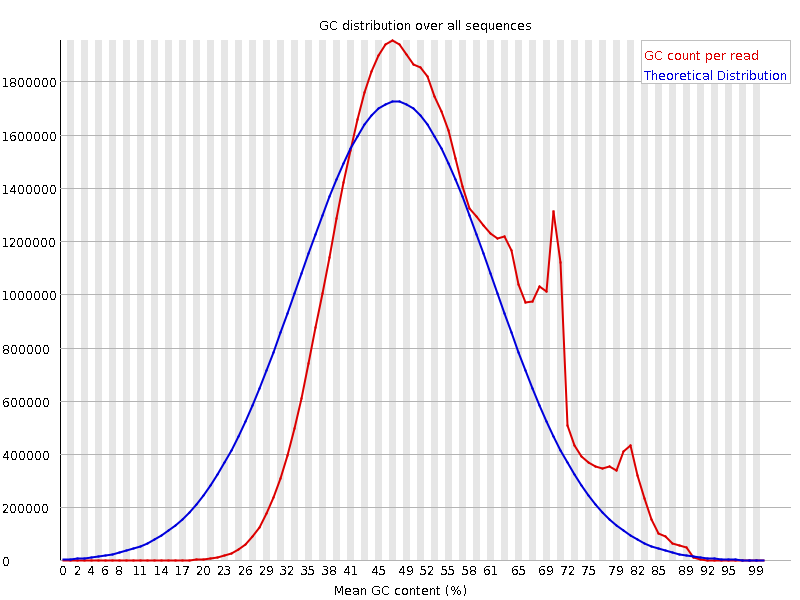

Supplement: Supplementary file 18 [file 953FileS3.zip › QC/A0-1_2.per_sequence_gc_content.png]

## Classification of Raw Reads(A0-2)

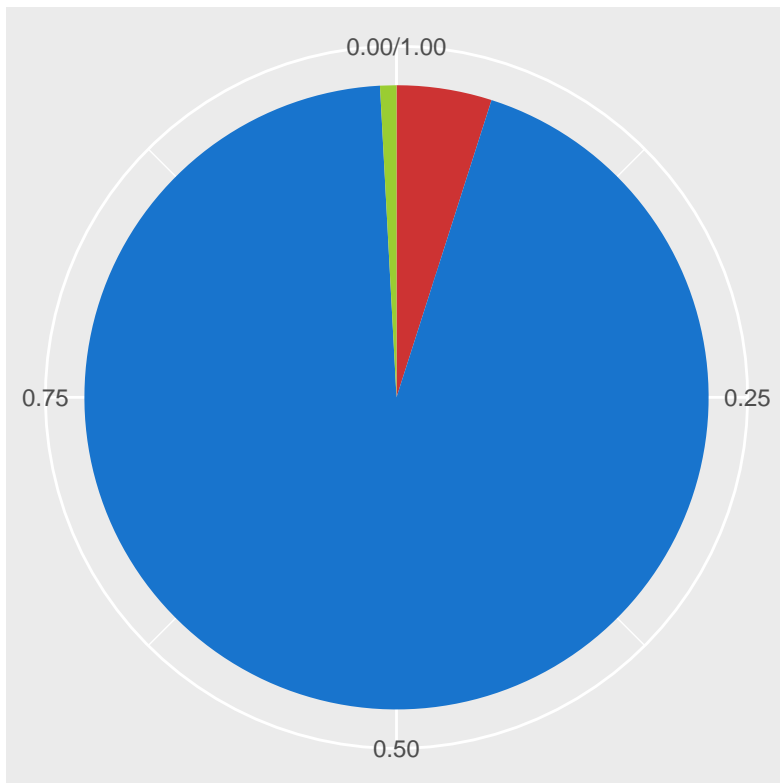

- Adapter(7224392,4.93%)
- Clean Reads(138217716,94.23%)
- Containing N(206,0.00%)
- Low Quality(1238552,0.84%)

Supplement: Supplementary file 18 [file 953FileS3.zip › QC/A0-2.qc.pdf]

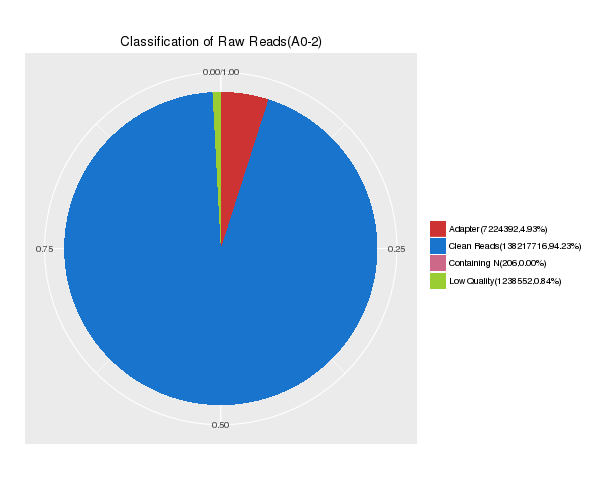

Supplement: Supplementary file 18 [file 953FileS3.zip › QC/A0-2.qc.png]

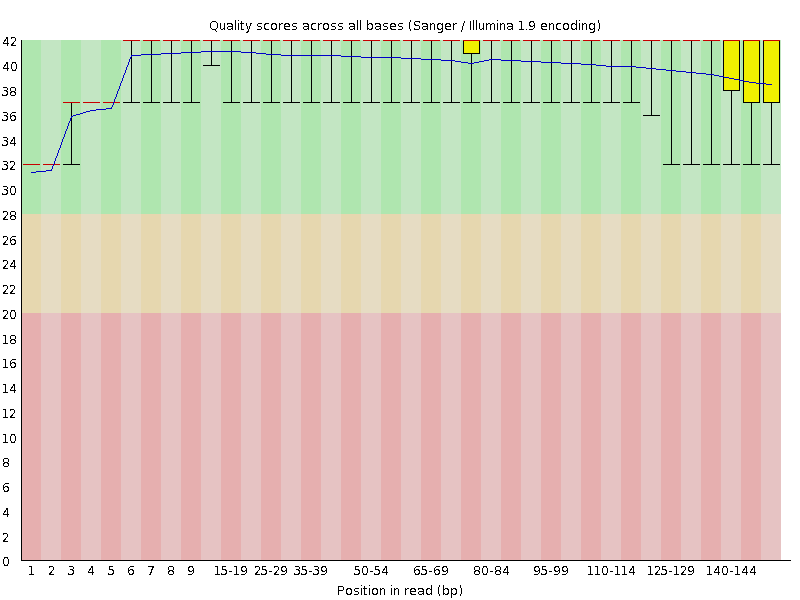

Supplement: Supplementary file 18 [file 953FileS3.zip › QC/A0-2_1.per_base_quality.png]

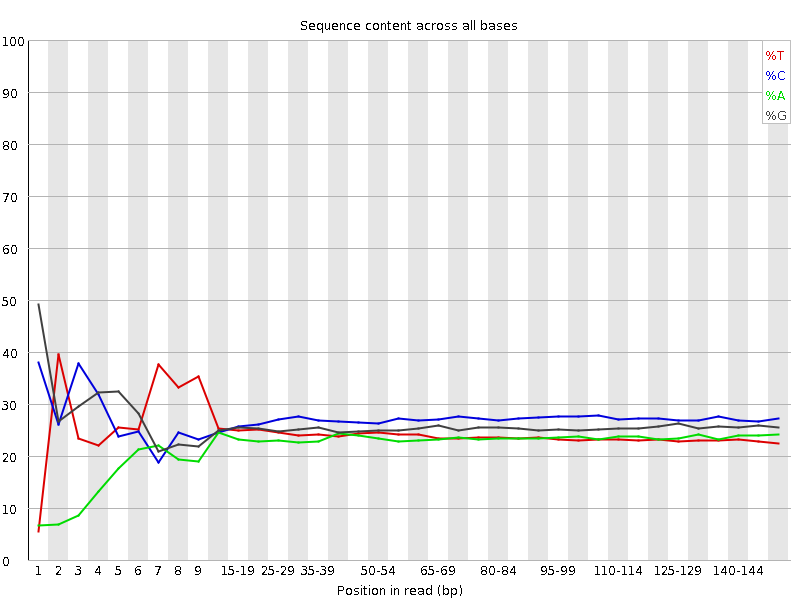

Supplement: Supplementary file 18 [file 953FileS3.zip › QC/A0-2_1.per_base_sequence_content.png]

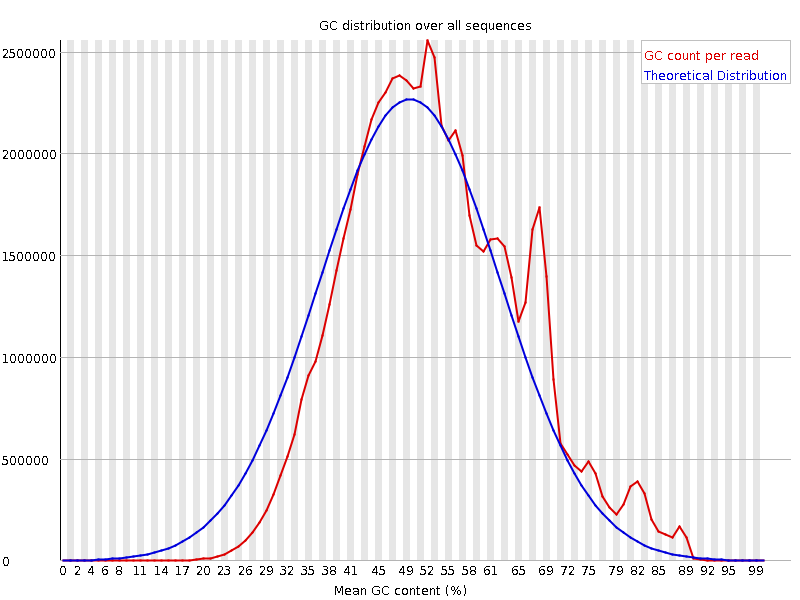

Supplement: Supplementary file 18 [file 953FileS3.zip › QC/A0-2_1.per_sequence_gc_content.png]

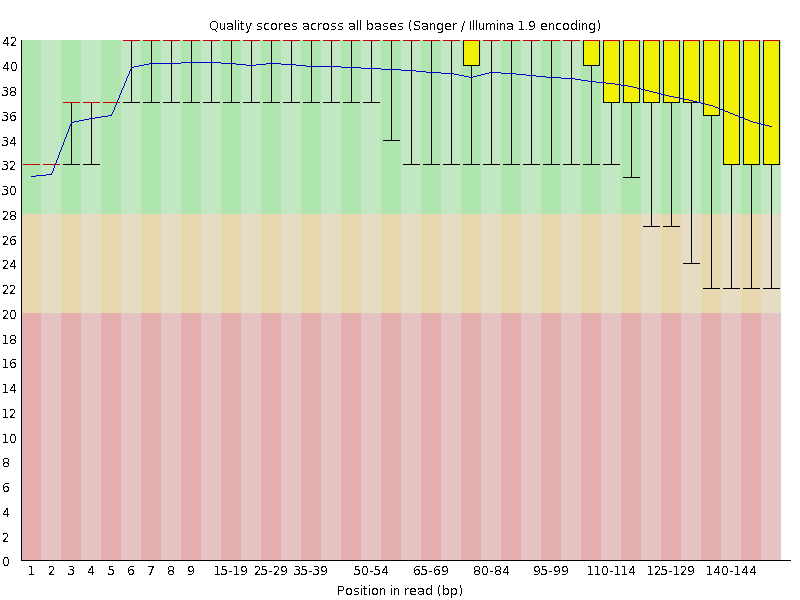

Supplement: Supplementary file 18 [file 953FileS3.zip › QC/A0-2_2.per_base_quality.png]

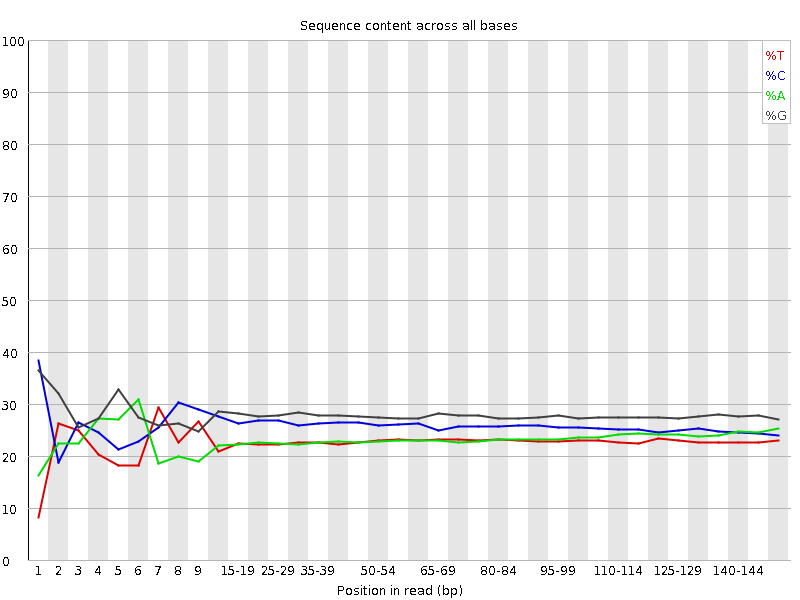

Supplement: Supplementary file 18 [file 953FileS3.zip › QC/A0-2_2.per_base_sequence_content.png]

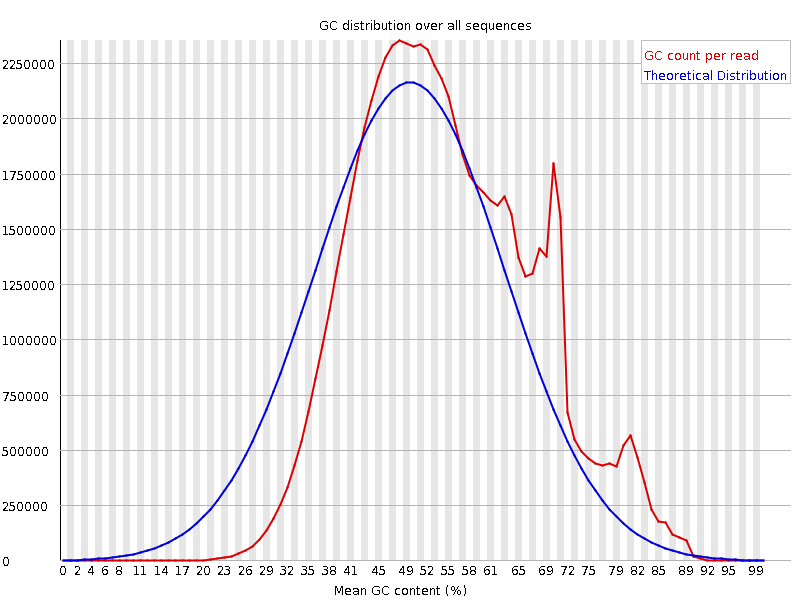

Supplement: Supplementary file 18 [file 953FileS3.zip › QC/A0-2_2.per_sequence_gc_content.png]

## Classification of Raw Reads(A0-3)

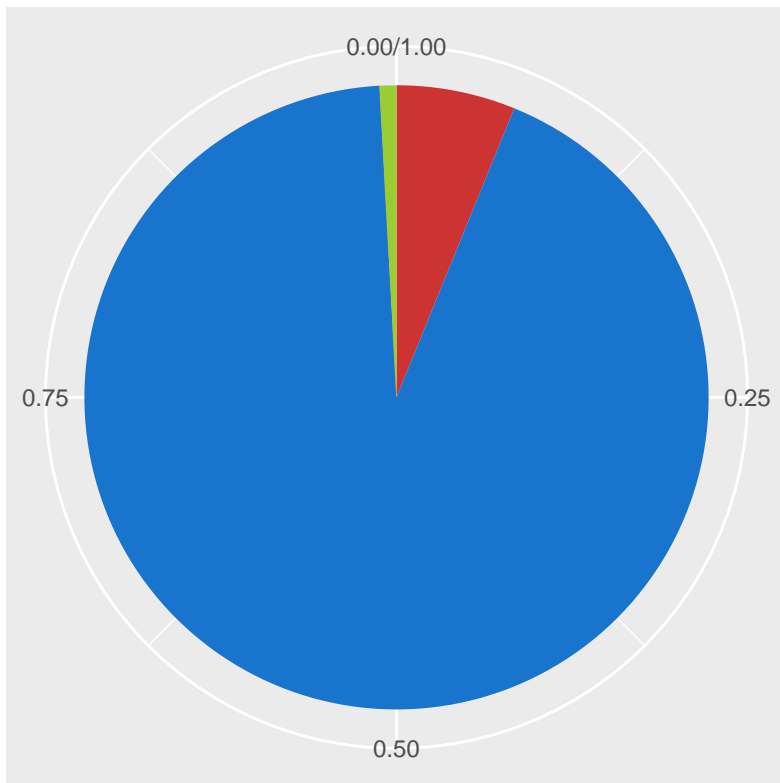

- Adapter(7299804,6.16%)
- Clean Reads(110196390,92.97%)
- Containing N(200,0.00%)
- Low Quality(1031846,0.87%)

Supplement: Supplementary file 18 [file 953FileS3.zip › QC/A0-3.qc.pdf]

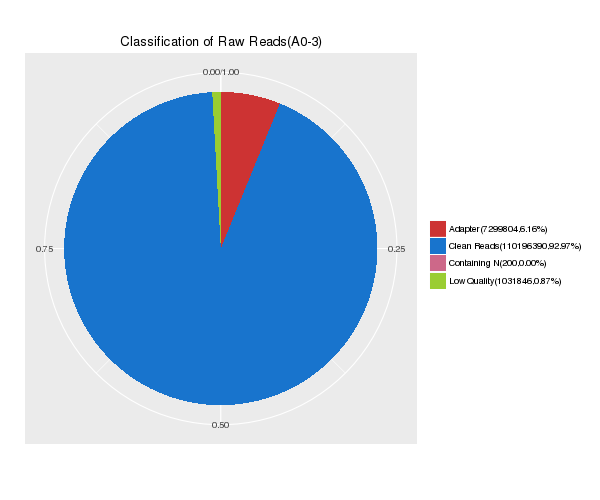

Supplement: Supplementary file 18 [file 953FileS3.zip › QC/A0-3.qc.png]

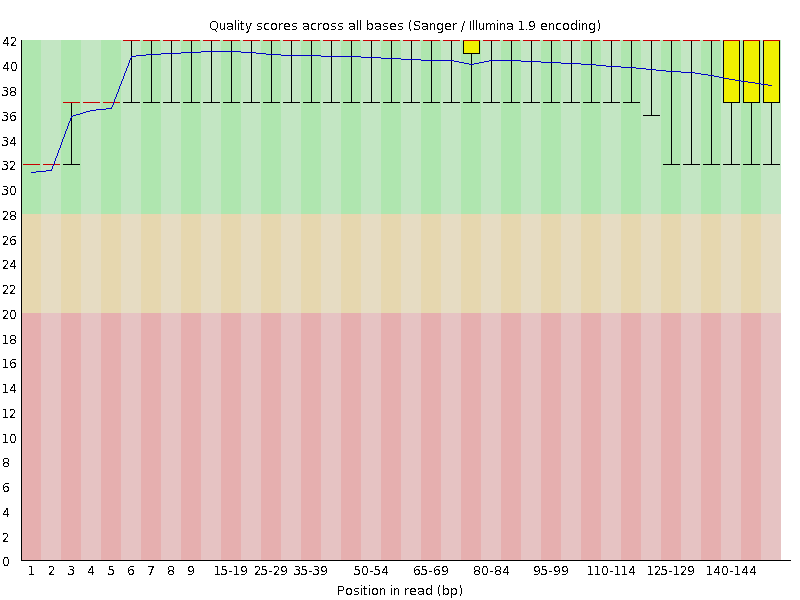

Supplement: Supplementary file 18 [file 953FileS3.zip › QC/A0-3_1.per_base_quality.png]

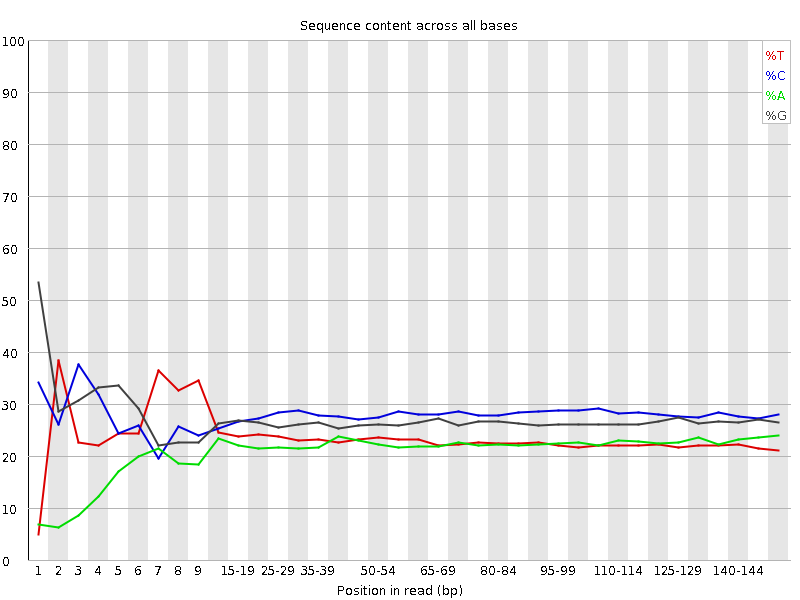

Supplement: Supplementary file 18 [file 953FileS3.zip › QC/A0-3_1.per_base_sequence_content.png]

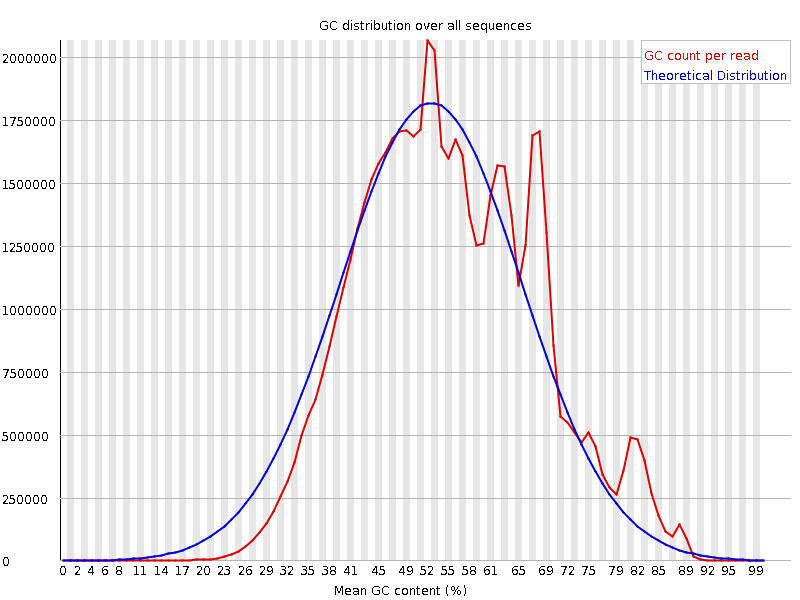

Supplement: Supplementary file 18 [file 953FileS3.zip › QC/A0-3_1.per_sequence_gc_content.png]

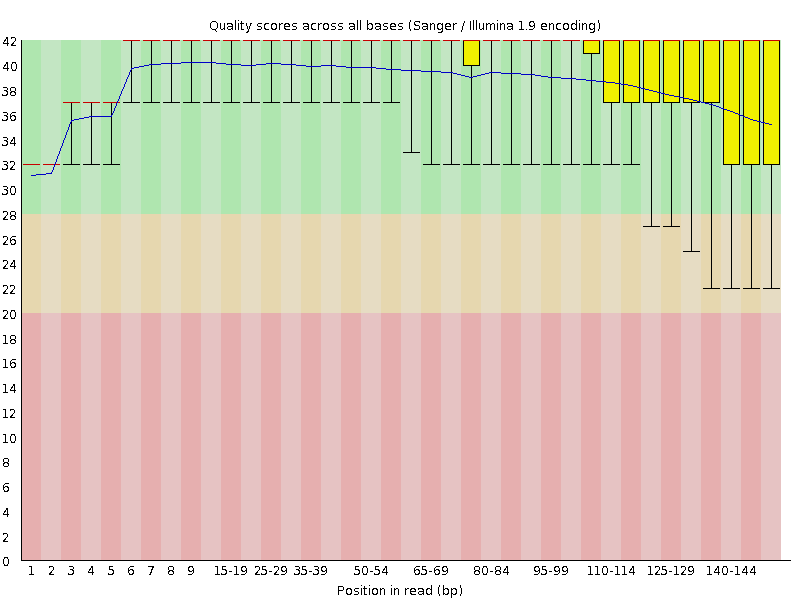

Supplement: Supplementary file 18 [file 953FileS3.zip › QC/A0-3_2.per_base_quality.png]

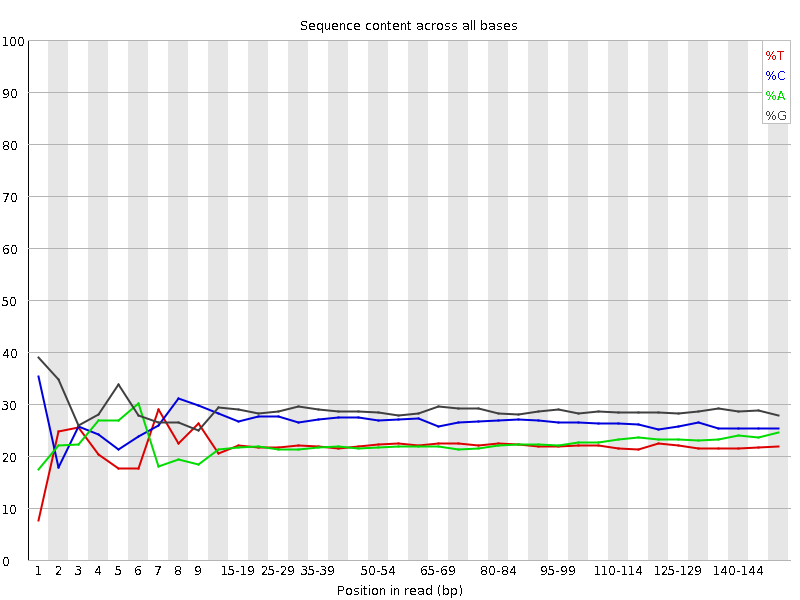

Supplement: Supplementary file 18 [file 953FileS3.zip › QC/A0-3_2.per_base_sequence_content.png]

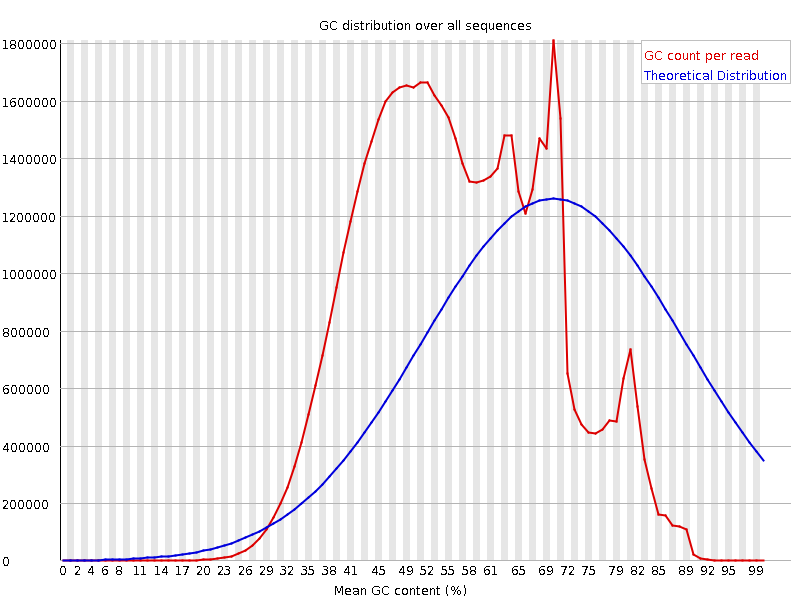

Supplement: Supplementary file 18 [file 953FileS3.zip › QC/A0-3_2.per_sequence_gc_content.png]

## Classification of Raw Reads(A2-1)

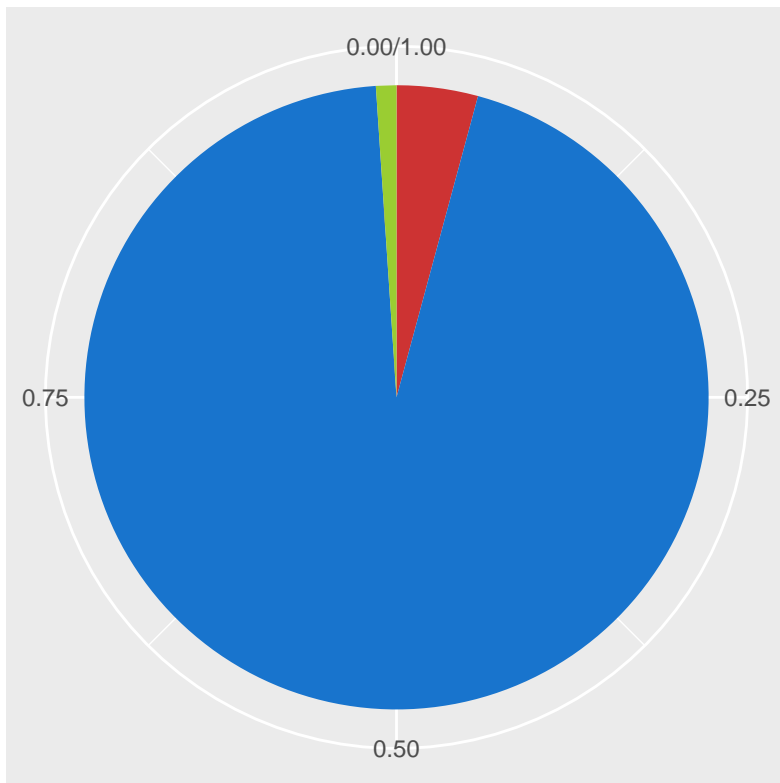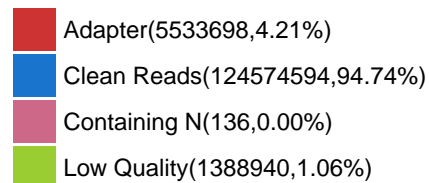

Supplement: Supplementary file 18 [file 953FileS3.zip › QC/A2-1.qc.pdf]

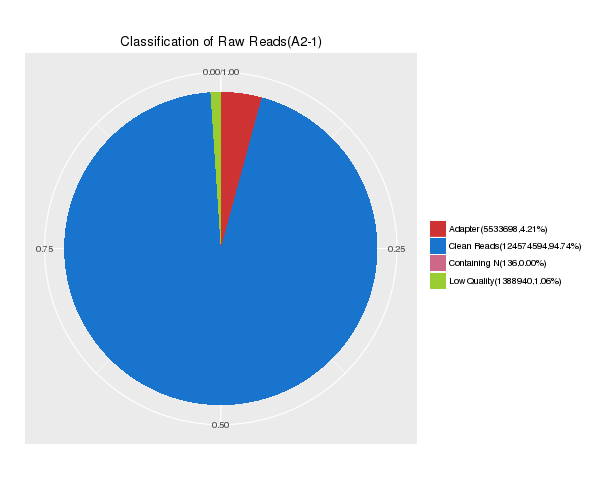

Supplement: Supplementary file 18 [file 953FileS3.zip › QC/A2-1.qc.png]

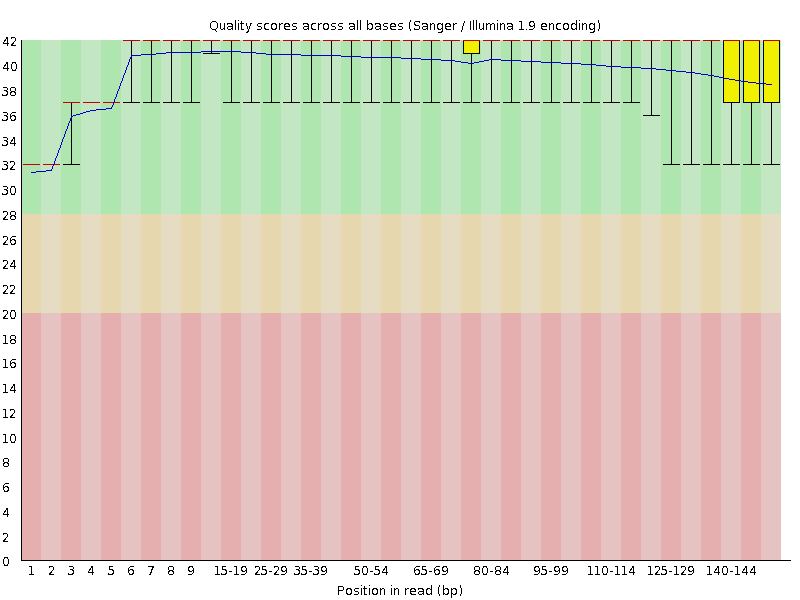

Supplement: Supplementary file 18 [file 953FileS3.zip › QC/A2-1_1.per_base_quality.png]

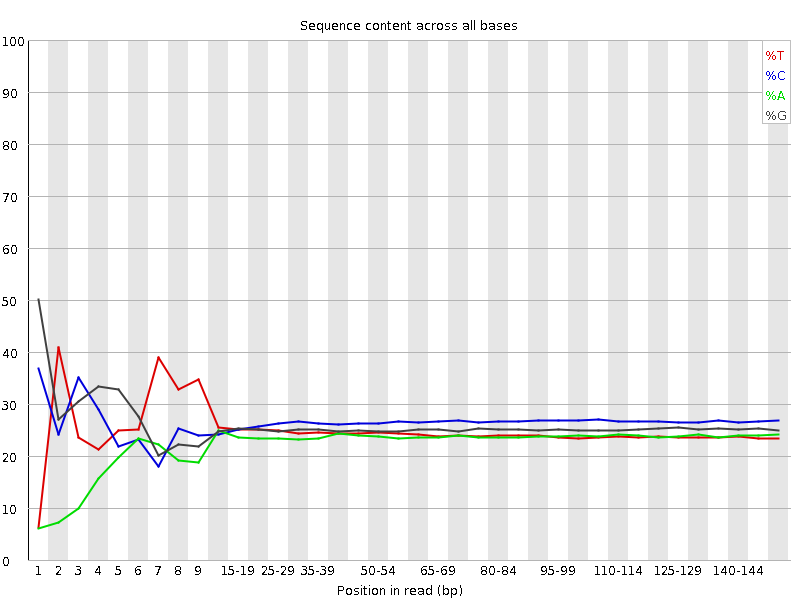

Supplement: Supplementary file 18 [file 953FileS3.zip › QC/A2-1_1.per_base_sequence_content.png]

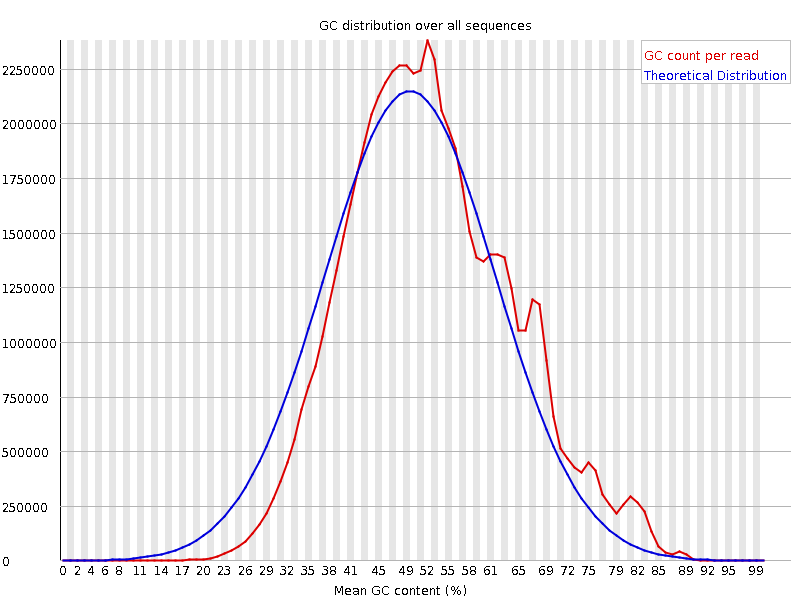

Supplement: Supplementary file 18 [file 953FileS3.zip › QC/A2-1_1.per_sequence_gc_content.png]

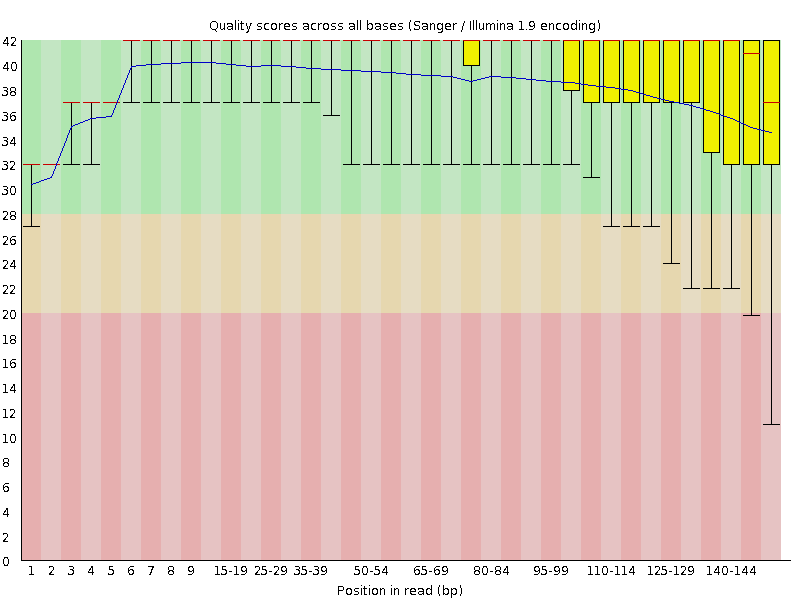

Supplement: Supplementary file 18 [file 953FileS3.zip › QC/A2-1_2.per_base_quality.png]

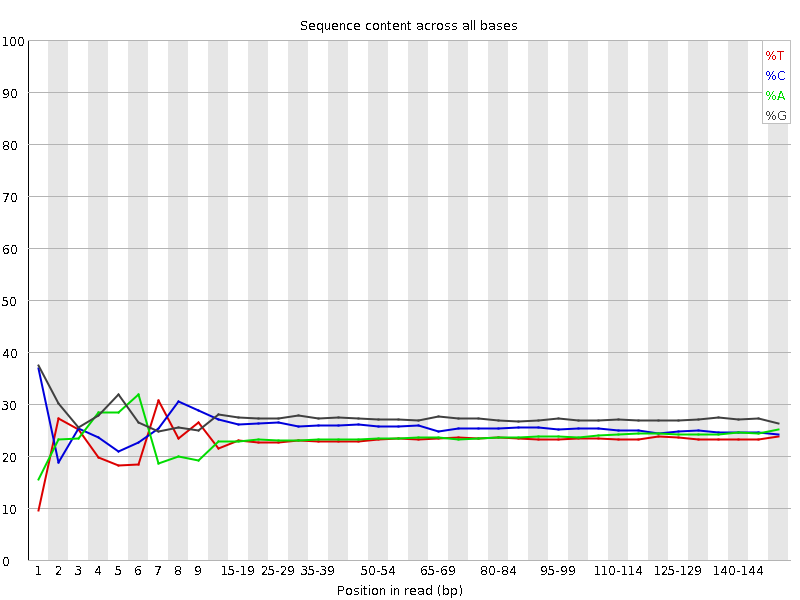

Supplement: Supplementary file 18 [file 953FileS3.zip › QC/A2-1_2.per_base_sequence_content.png]

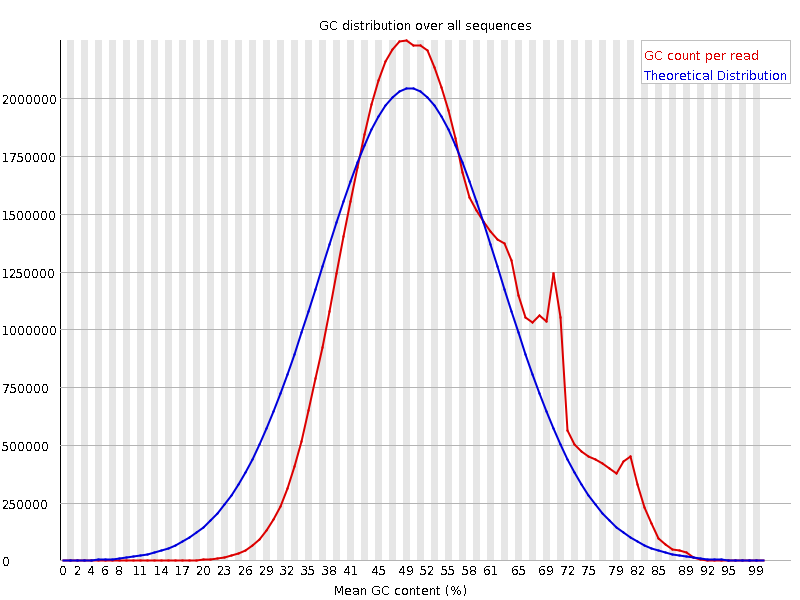

Supplement: Supplementary file 18 [file 953FileS3.zip › QC/A2-1_2.per_sequence_gc_content.png]

## Classification of Raw Reads(A2-2)

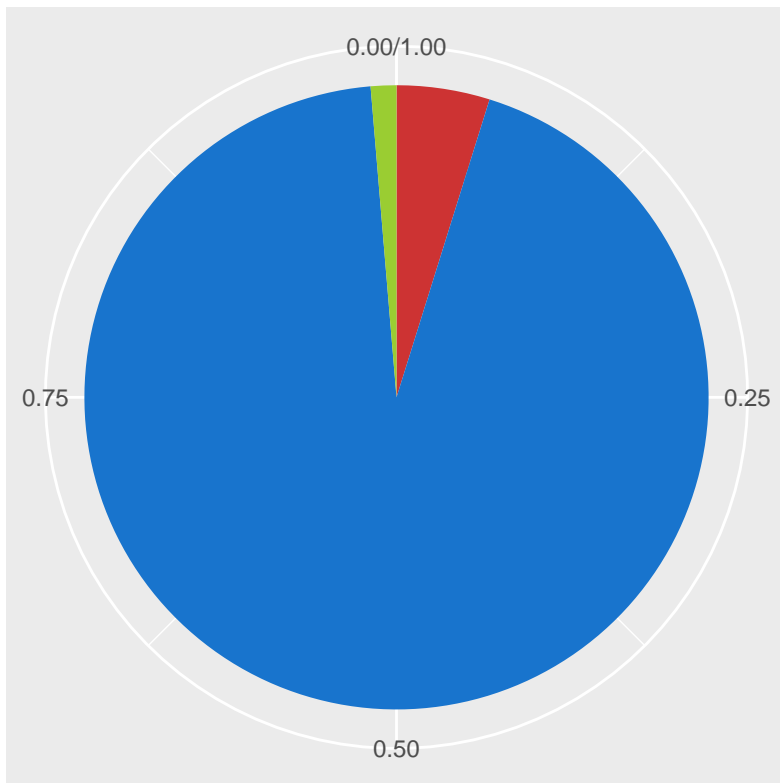

- Adapter(5356306,4.82%)
- Clean Reads(104305988,93.85%)
- Containing N(96,0.00%)
- Low Quality(1473594,1.33%)

Supplement: Supplementary file 18 [file 953FileS3.zip › QC/A2-2.qc.pdf]

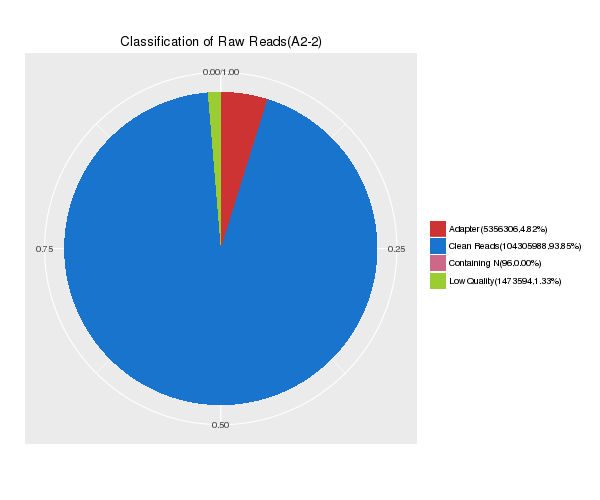

Supplement: Supplementary file 18 [file 953FileS3.zip › QC/A2-2.qc.png]

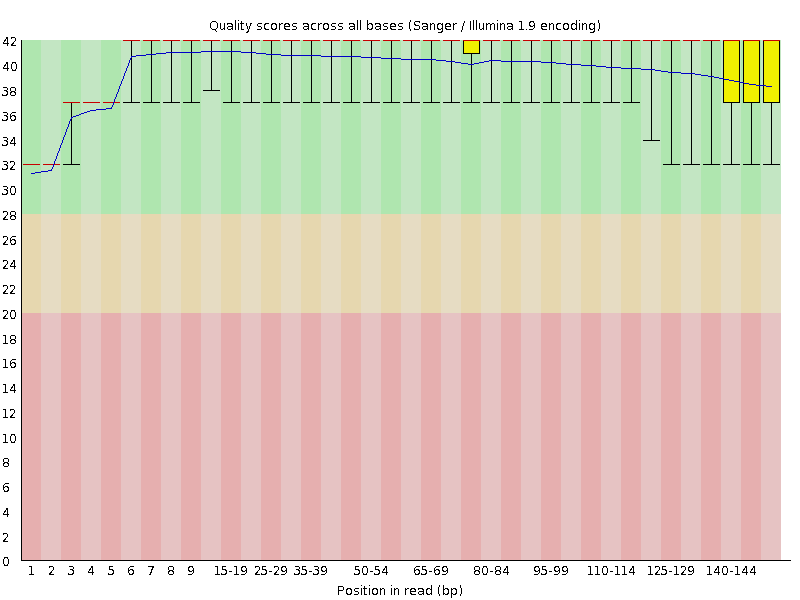

Supplement: Supplementary file 18 [file 953FileS3.zip › QC/A2-2_1.per_base_quality.png]

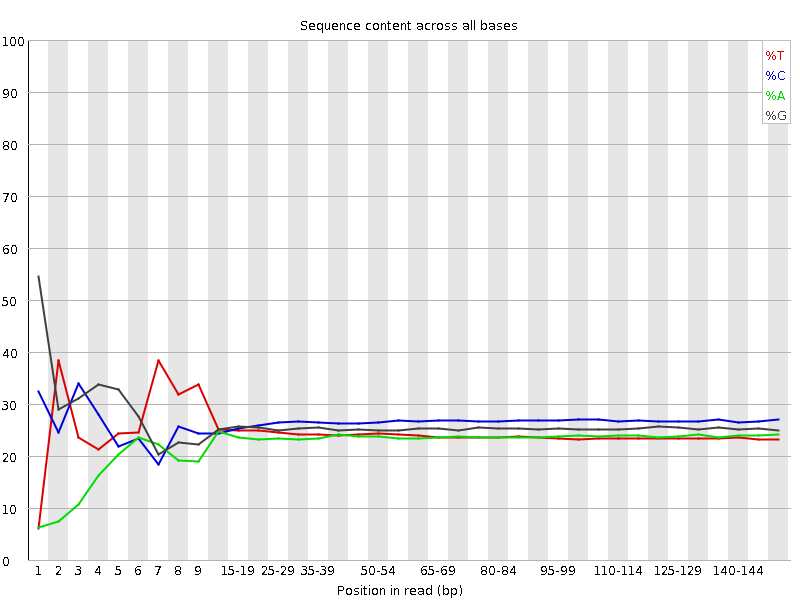

Supplement: Supplementary file 18 [file 953FileS3.zip › QC/A2-2_1.per_base_sequence_content.png]

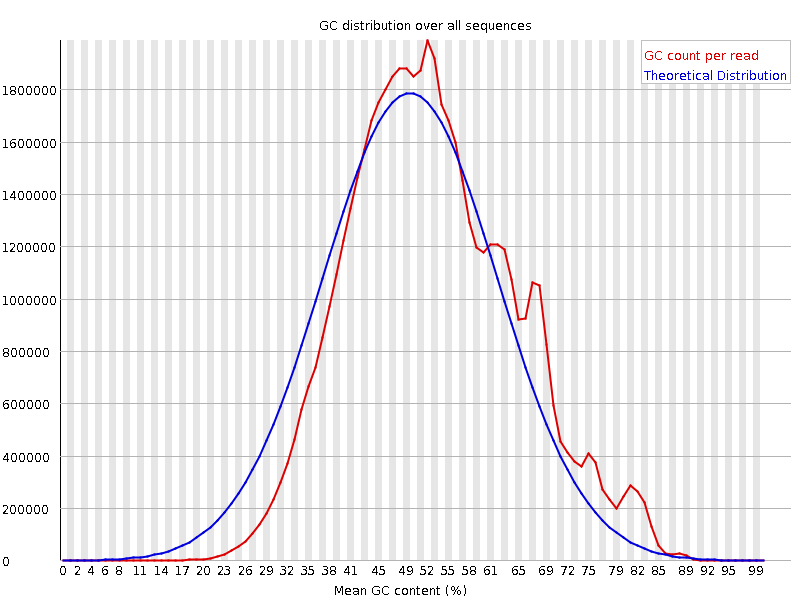

Supplement: Supplementary file 18 [file 953FileS3.zip › QC/A2-2_1.per_sequence_gc_content.png]

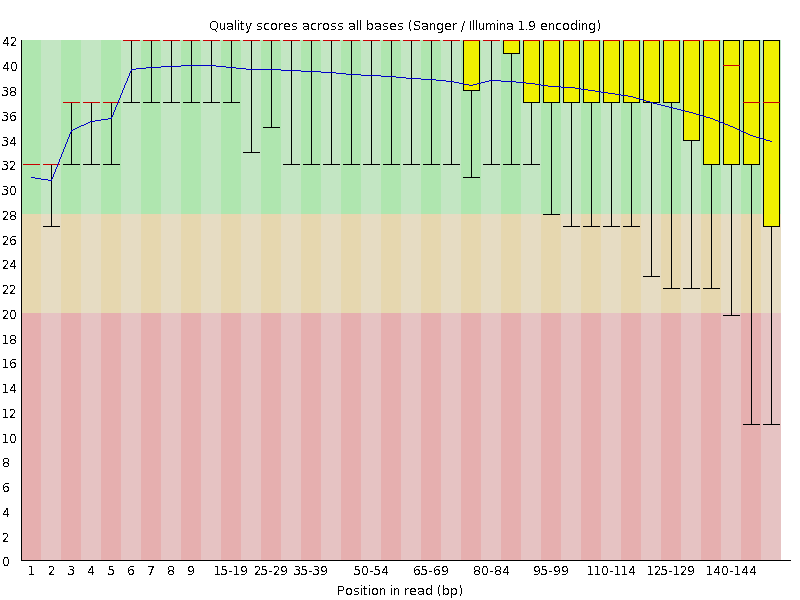

Supplement: Supplementary file 18 [file 953FileS3.zip › QC/A2-2_2.per_base_quality.png]

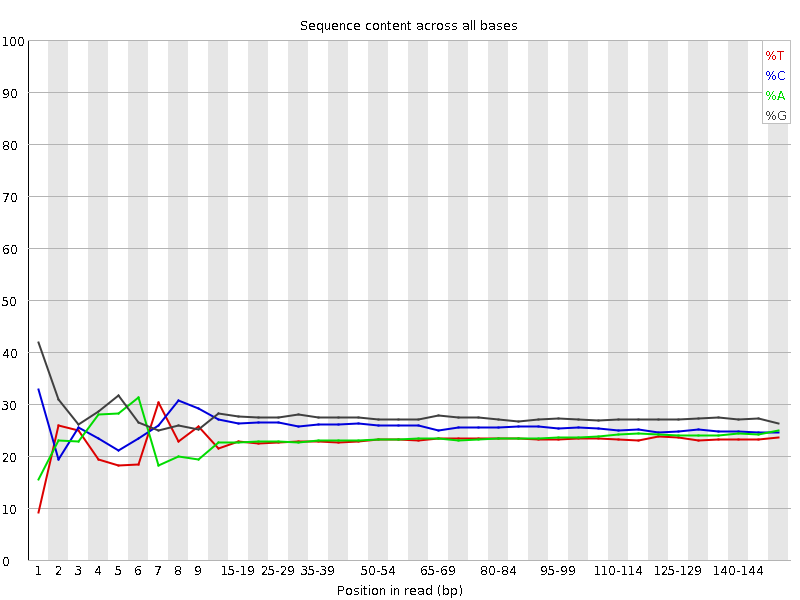

Supplement: Supplementary file 18 [file 953FileS3.zip › QC/A2-2_2.per_base_sequence_content.png]

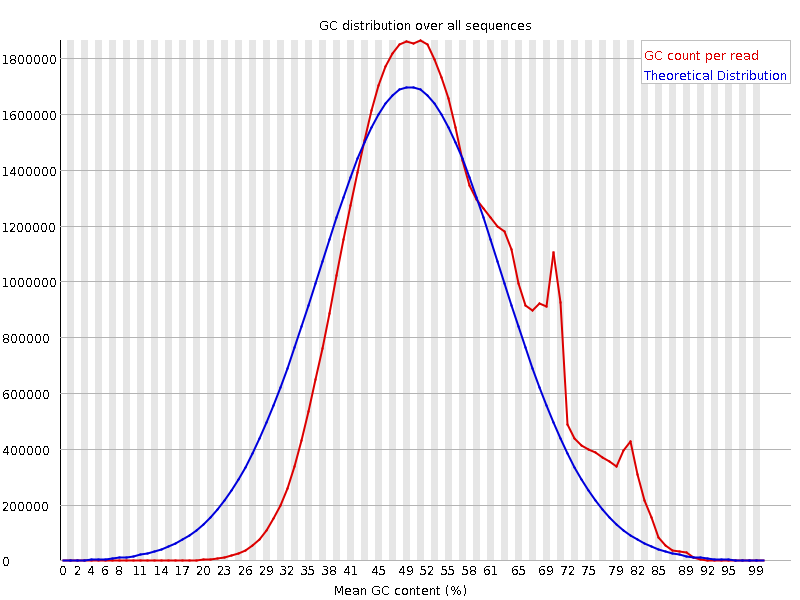

Supplement: Supplementary file 18 [file 953FileS3.zip › QC/A2-2_2.per_sequence_gc_content.png]

## Classification of Raw Reads(A2-3)

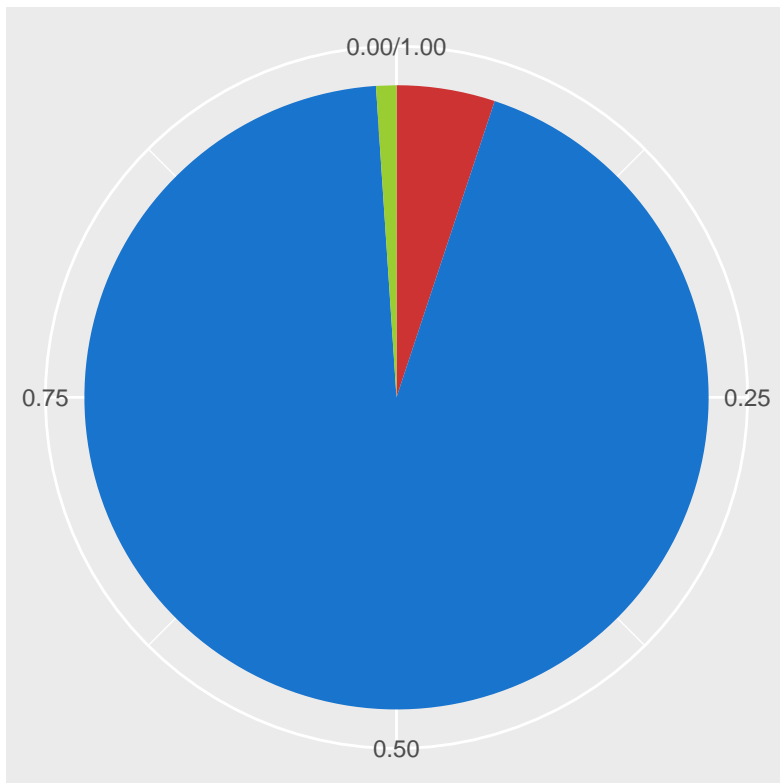

- Adapter(6379248,5.09%)
- Clean Reads(117527324,93.85%)
- Containing N(118,0.00%)
- Low Quality(1316736,1.05%)

Supplement: Supplementary file 18 [file 953FileS3.zip › QC/A2-3.qc.pdf]

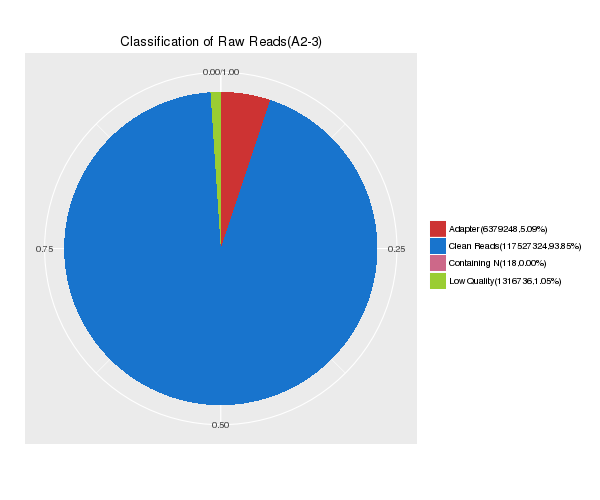

Supplement: Supplementary file 18 [file 953FileS3.zip › QC/A2-3.qc.png]

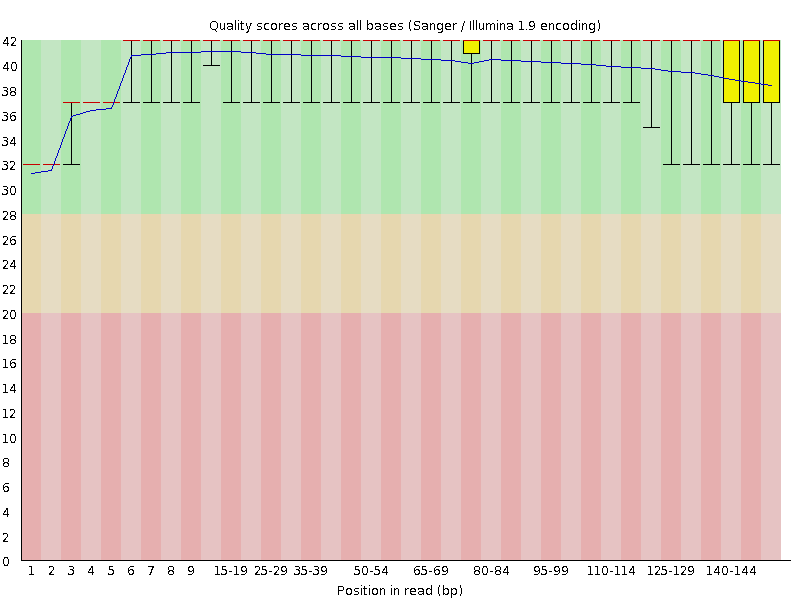

Supplement: Supplementary file 18 [file 953FileS3.zip › QC/A2-3_1.per_base_quality.png]

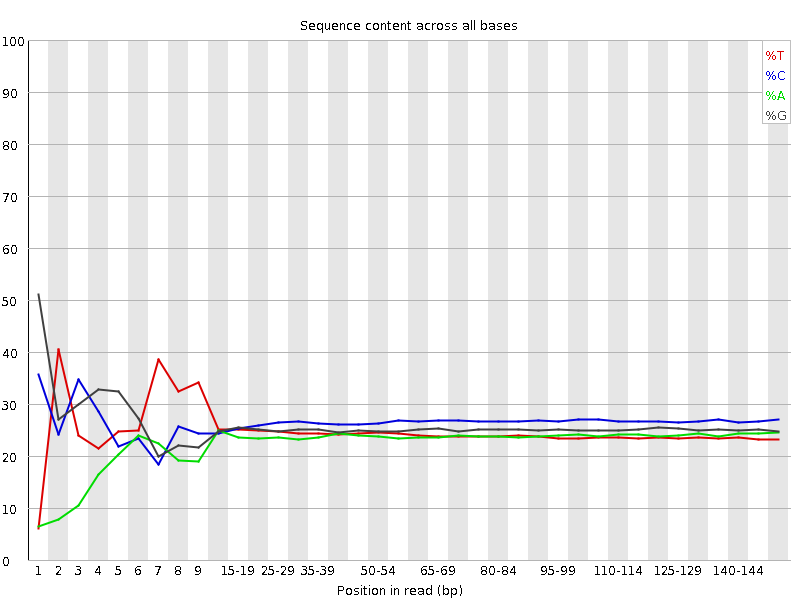

Supplement: Supplementary file 18 [file 953FileS3.zip › QC/A2-3_1.per_base_sequence_content.png]

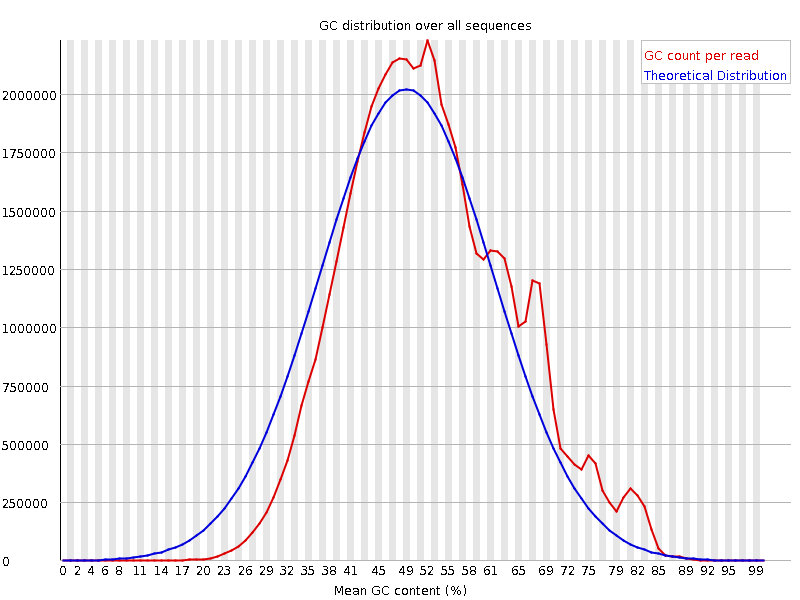

Supplement: Supplementary file 18 [file 953FileS3.zip › QC/A2-3_1.per_sequence_gc_content.png]

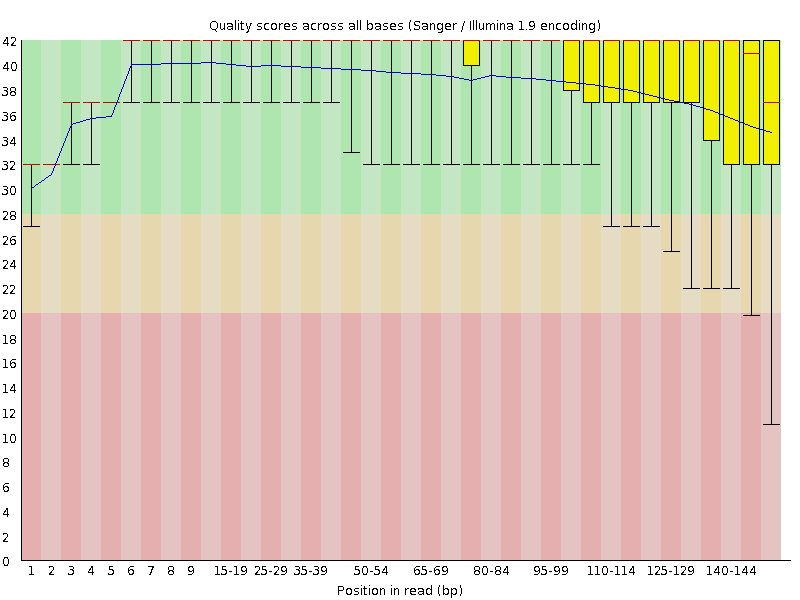

Supplement: Supplementary file 18 [file 953FileS3.zip › QC/A2-3_2.per_base_quality.png]

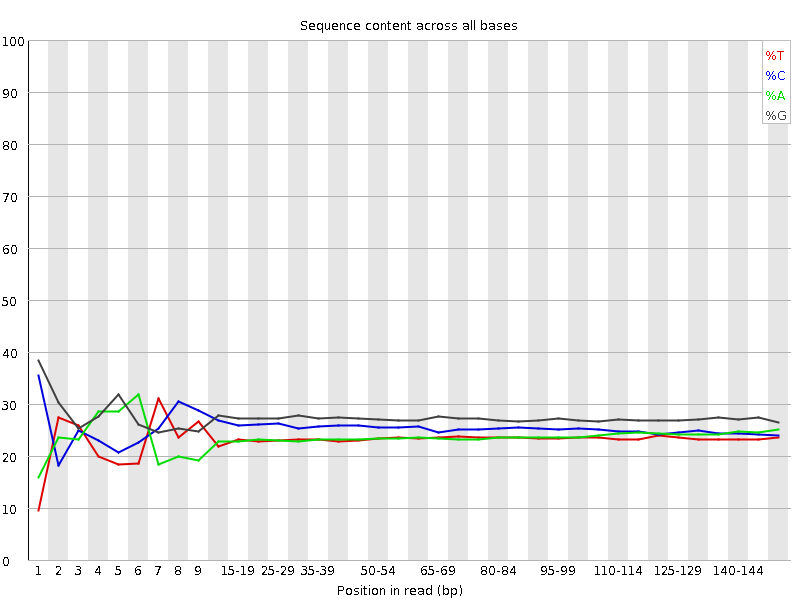

Supplement: Supplementary file 18 [file 953FileS3.zip › QC/A2-3_2.per_base_sequence_content.png]

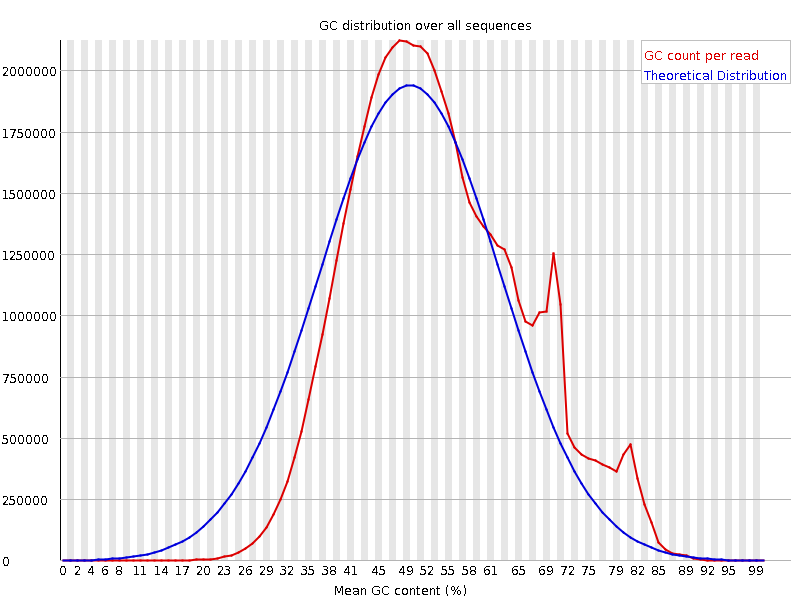

Supplement: Supplementary file 18 [file 953FileS3.zip › QC/A2-3_2.per_sequence_gc_content.png]

## Classification of Raw Reads(A4-1)

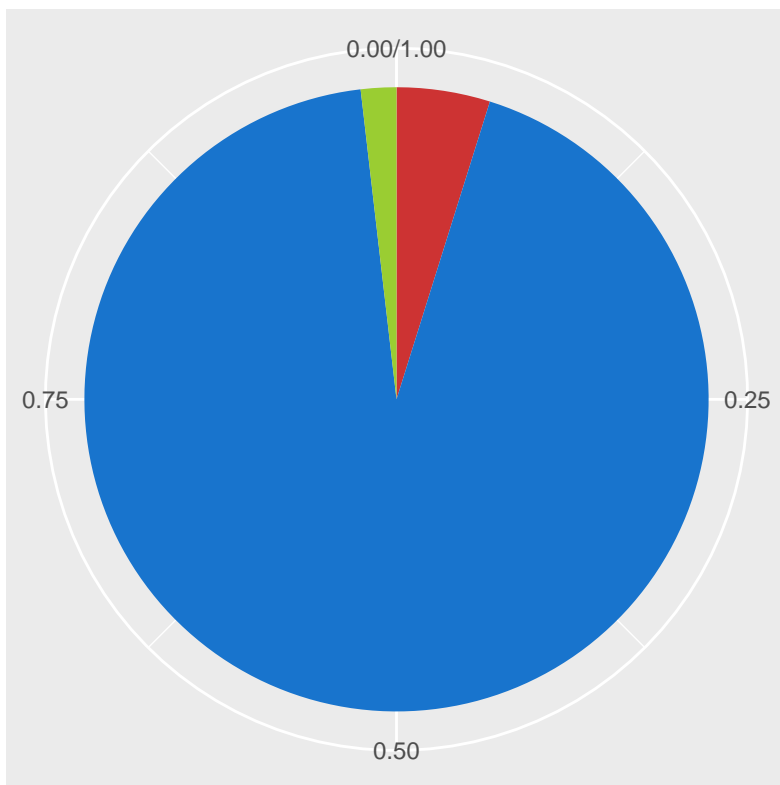

- Adapter(5353376,4.84%)
- Clean Reads(103271936,93.33%)
- Containing N(80,0.00%)
- Low Quality(2031948,1.84%)

Supplement: Supplementary file 18 [file 953FileS3.zip › QC/A4-1.qc.pdf]

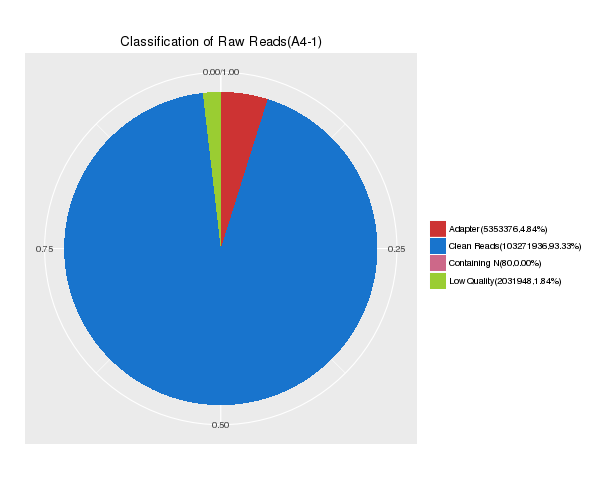

Supplement: Supplementary file 18 [file 953FileS3.zip › QC/A4-1.qc.png]

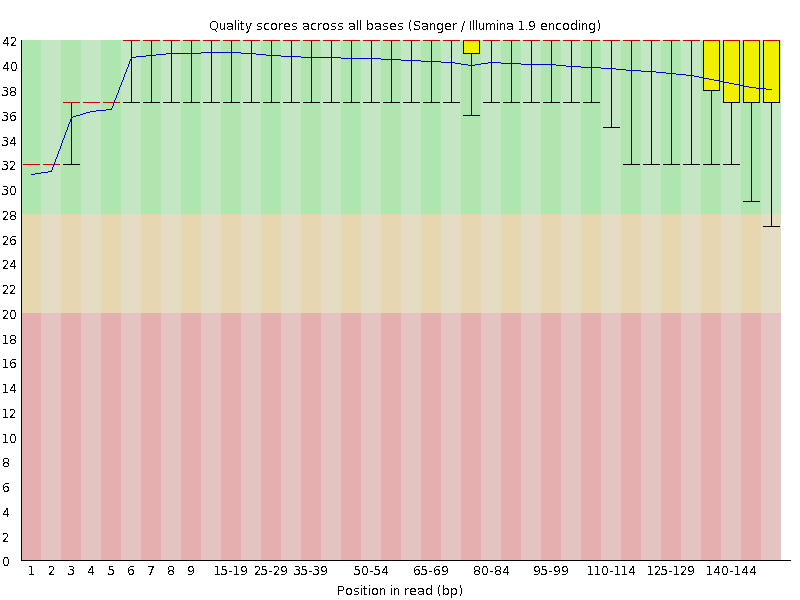

Supplement: Supplementary file 18 [file 953FileS3.zip › QC/A4-1_1.per_base_quality.png]

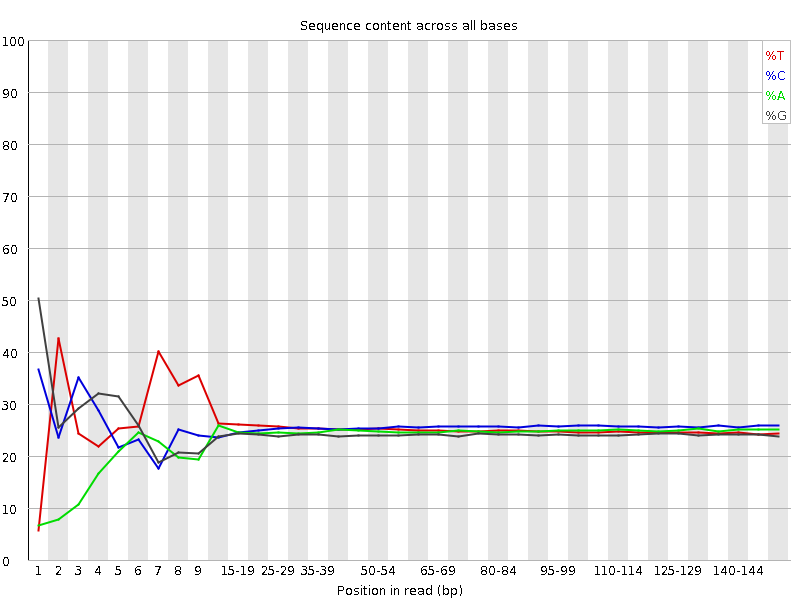

Supplement: Supplementary file 18 [file 953FileS3.zip › QC/A4-1_1.per_base_sequence_content.png]

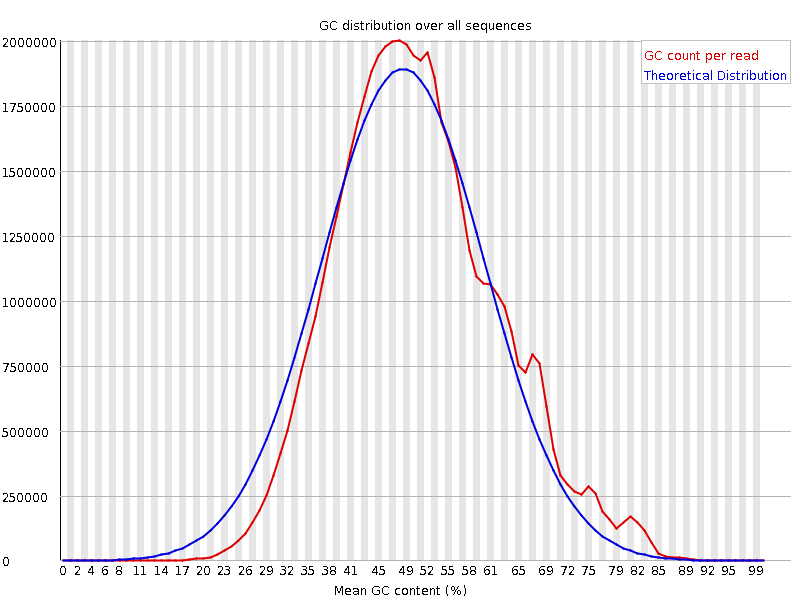

Supplement: Supplementary file 18 [file 953FileS3.zip › QC/A4-1_1.per_sequence_gc_content.png]

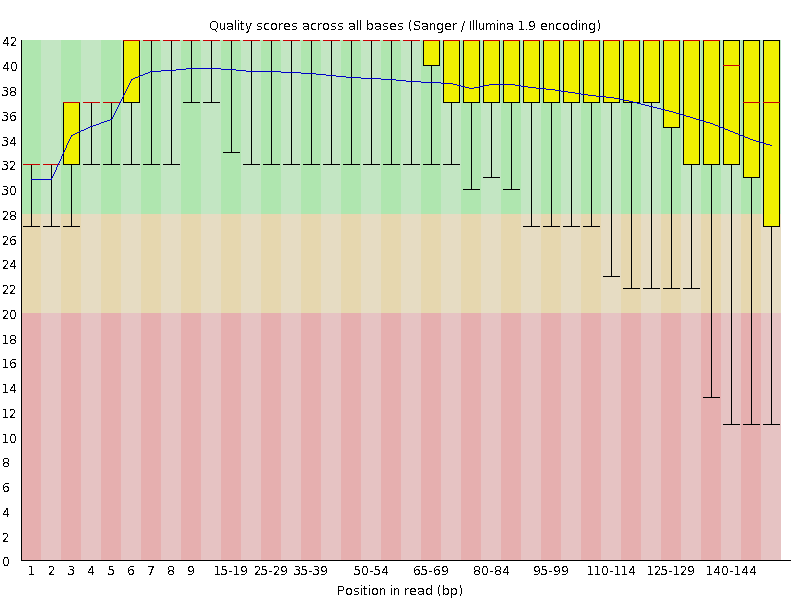

Supplement: Supplementary file 18 [file 953FileS3.zip › QC/A4-1_2.per_base_quality.png]

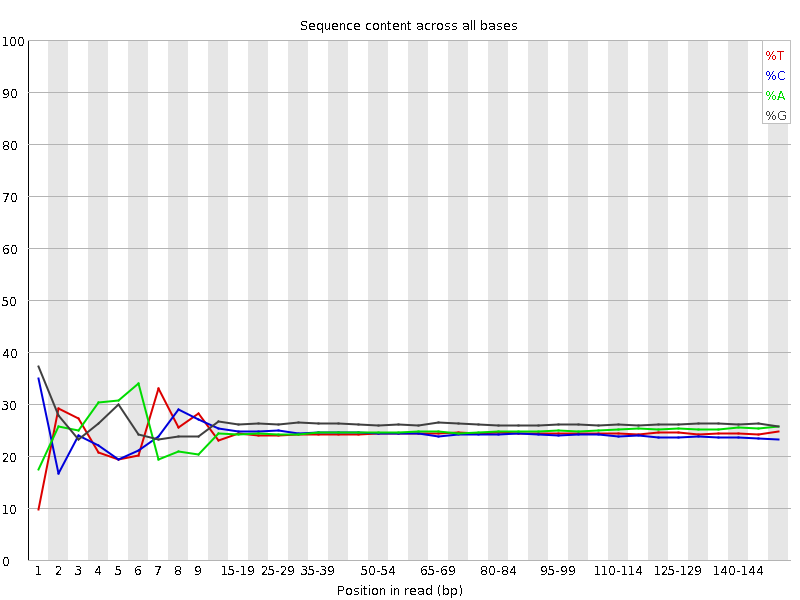

Supplement: Supplementary file 18 [file 953FileS3.zip › QC/A4-1_2.per_base_sequence_content.png]

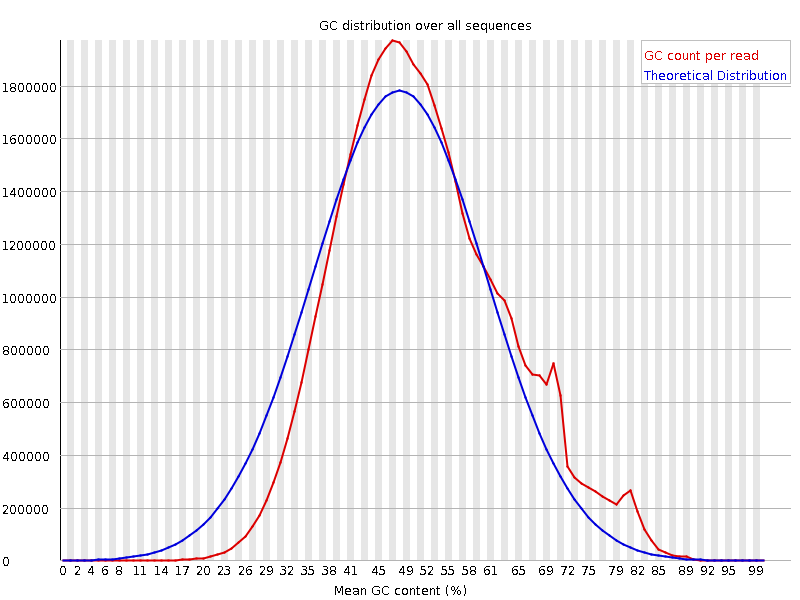

Supplement: Supplementary file 18 [file 953FileS3.zip › QC/A4-1_2.per_sequence_gc_content.png]

## Classification of Raw Reads(A4-2)

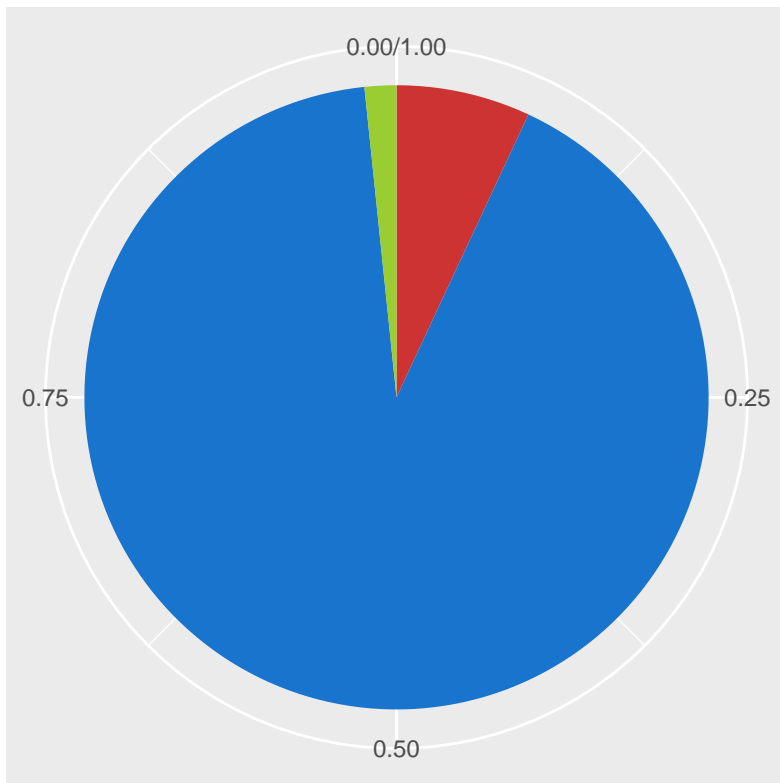

- Adapter(9033192,6.95%)
- Clean Reads(118853588,91.41%)
- Containing N(80,0.00%)
- Low Quality(2136434,1.64%)

Supplement: Supplementary file 18 [file 953FileS3.zip › QC/A4-2.qc.pdf]

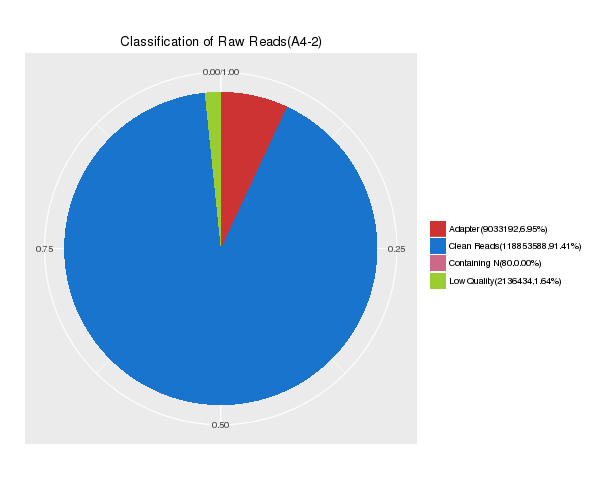

Supplement: Supplementary file 18 [file 953FileS3.zip › QC/A4-2.qc.png]

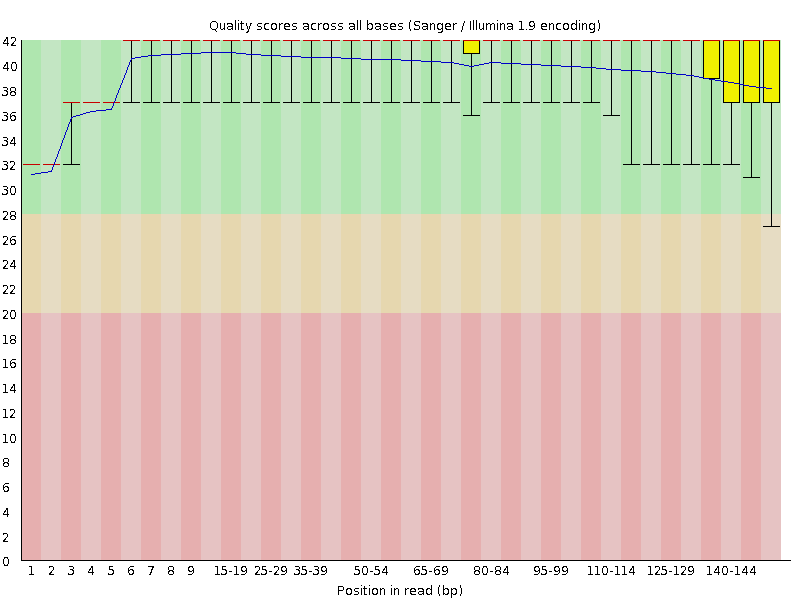

Supplement: Supplementary file 18 [file 953FileS3.zip › QC/A4-2_1.per_base_quality.png]

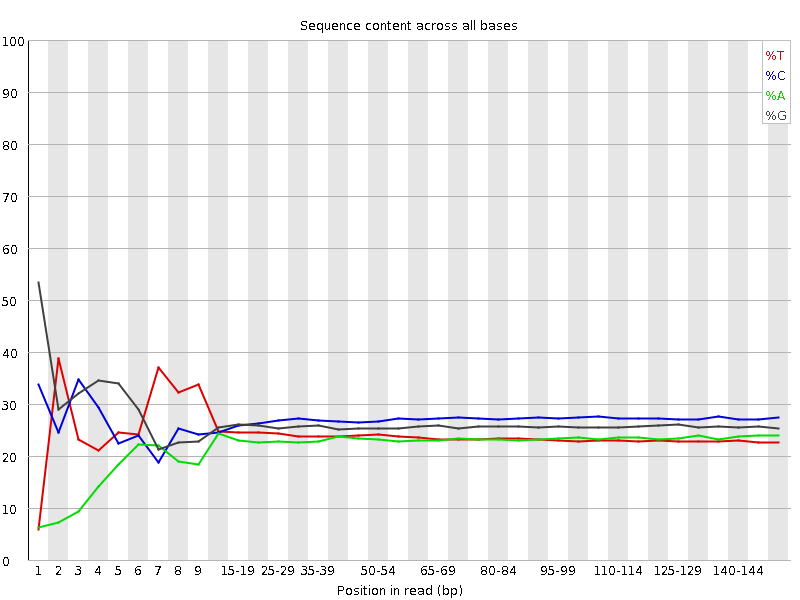

Supplement: Supplementary file 18 [file 953FileS3.zip › QC/A4-2_1.per_base_sequence_content.png]

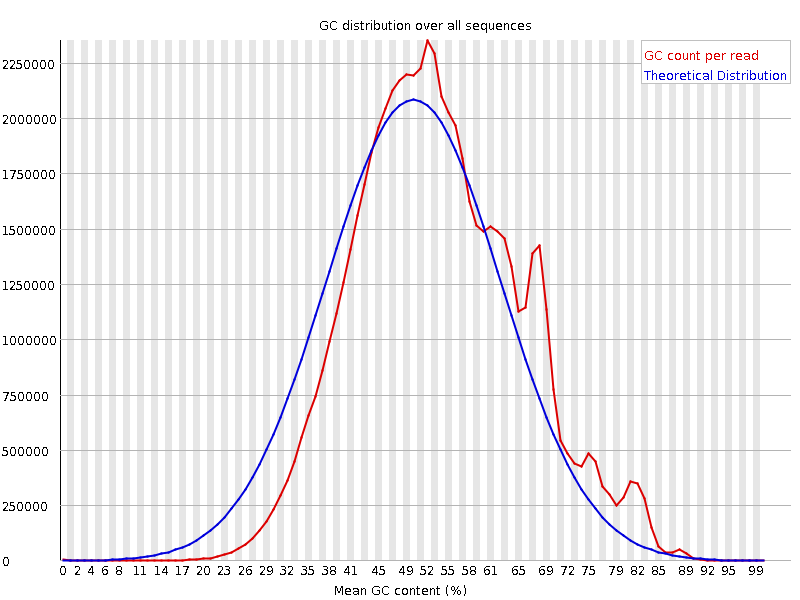

Supplement: Supplementary file 18 [file 953FileS3.zip › QC/A4-2_1.per_sequence_gc_content.png]

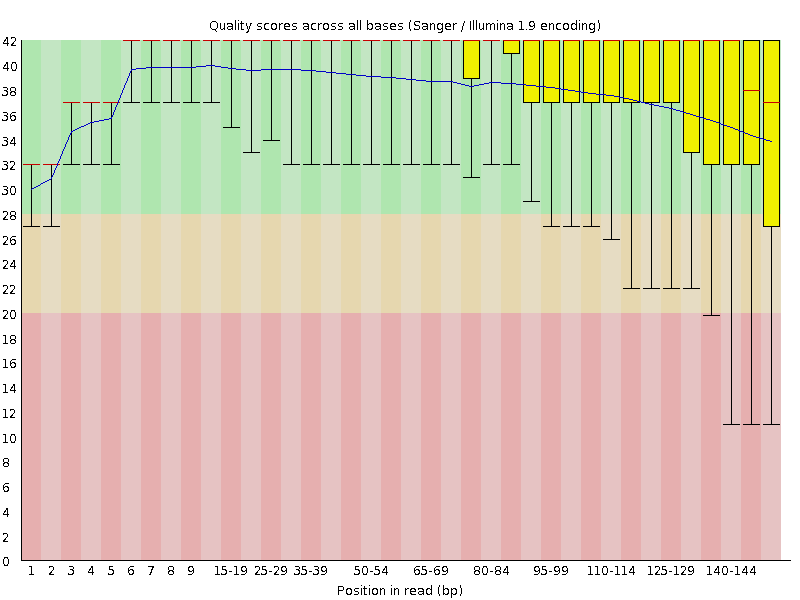

Supplement: Supplementary file 18 [file 953FileS3.zip › QC/A4-2_2.per_base_quality.png]

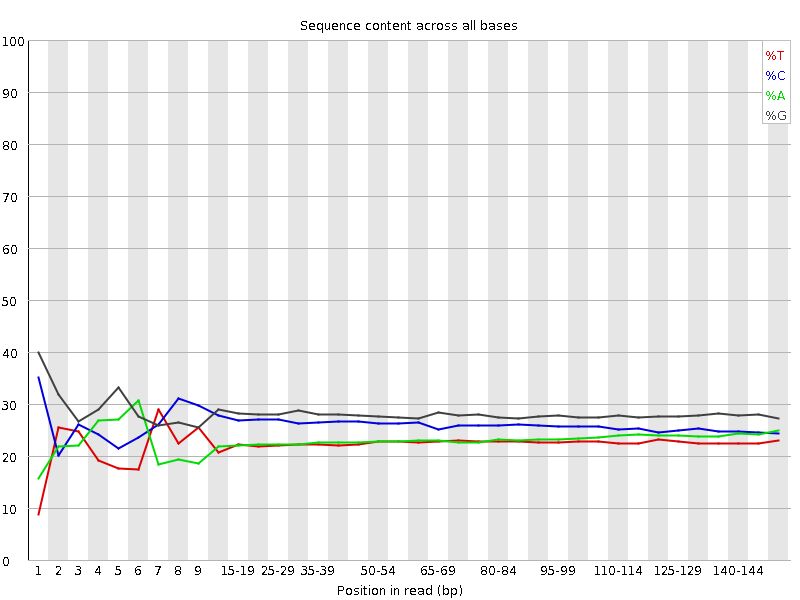

Supplement: Supplementary file 18 [file 953FileS3.zip › QC/A4-2_2.per_base_sequence_content.png]

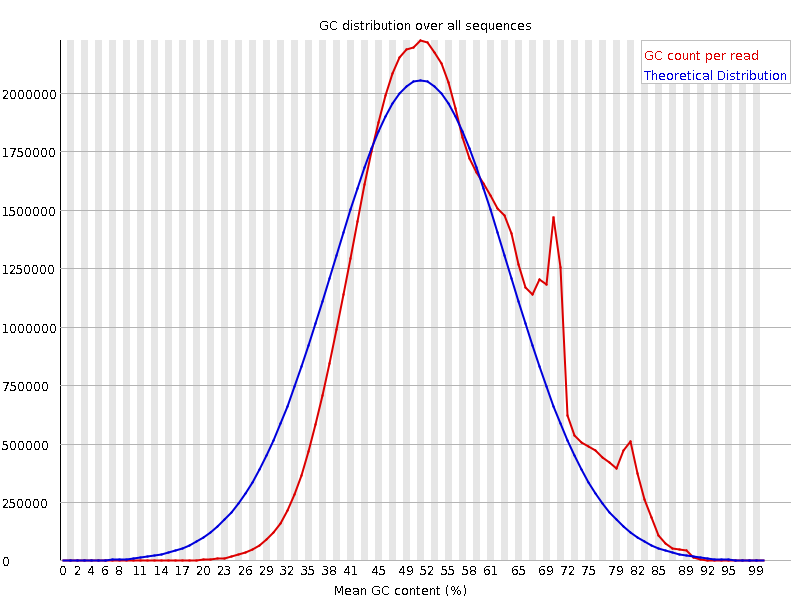

Supplement: Supplementary file 18 [file 953FileS3.zip › QC/A4-2_2.per_sequence_gc_content.png]

## Classification of Raw Reads(A4-3)

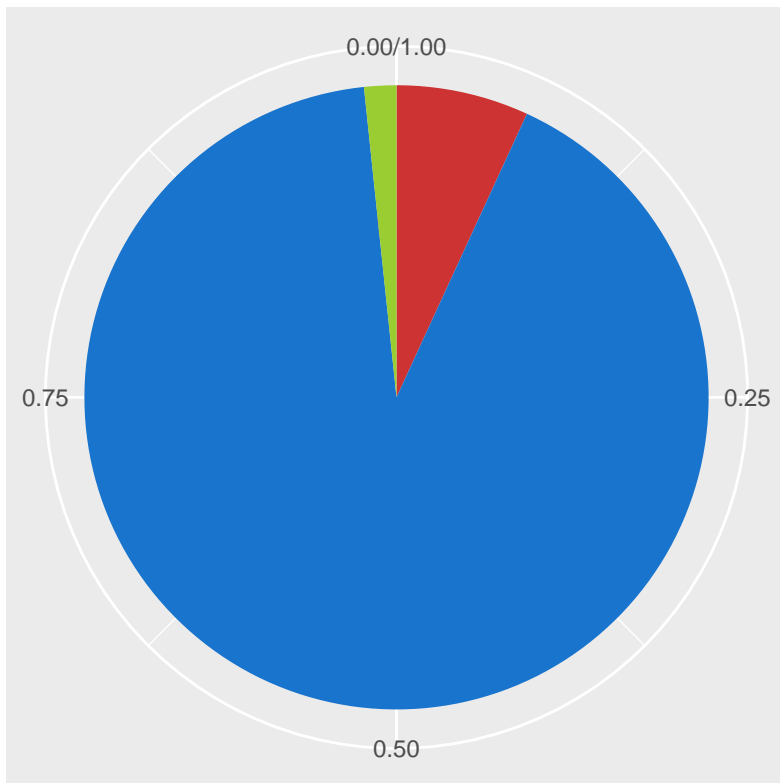

- Adapter(8735798,6.86%)
- Clean Reads(116411784,91.47%)
- Containing N(94,0.00%)
- Low Quality(2120874,1.67%)

Supplement: Supplementary file 18 [file 953FileS3.zip › QC/A4-3.qc.pdf]

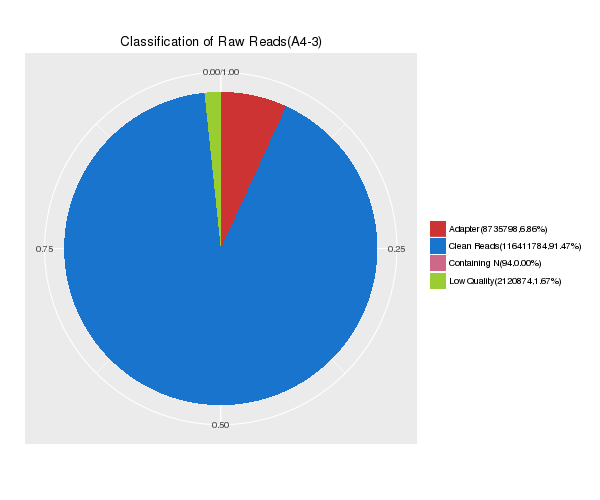

Supplement: Supplementary file 18 [file 953FileS3.zip › QC/A4-3.qc.png]

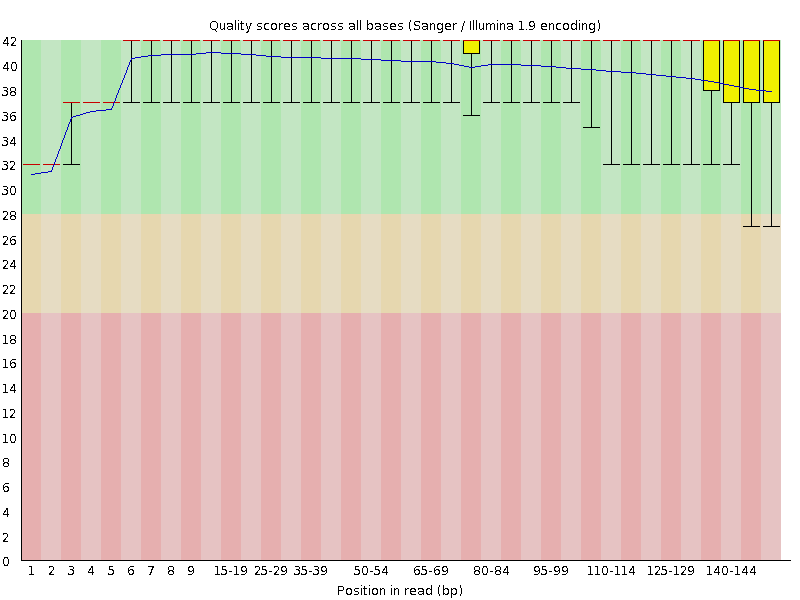

Supplement: Supplementary file 18 [file 953FileS3.zip › QC/A4-3_1.per_base_quality.png]

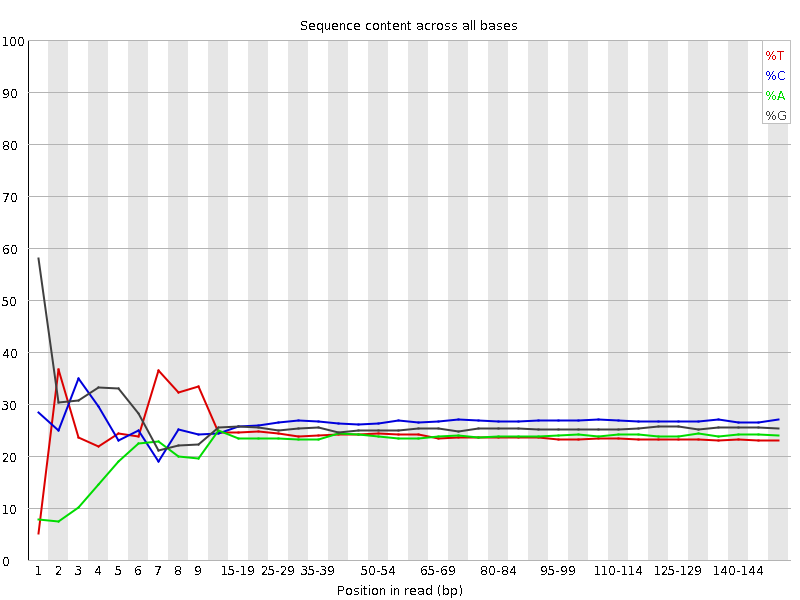

Supplement: Supplementary file 18 [file 953FileS3.zip › QC/A4-3_1.per_base_sequence_content.png]

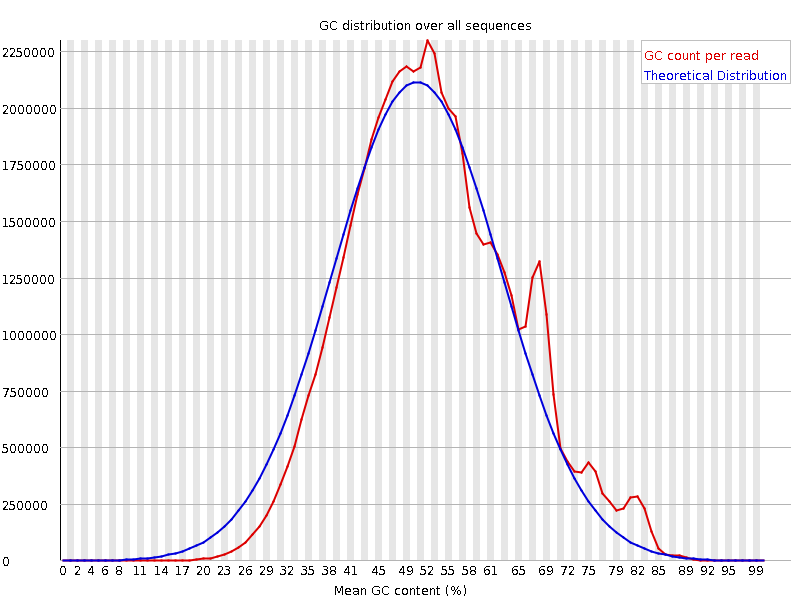

Supplement: Supplementary file 18 [file 953FileS3.zip › QC/A4-3_1.per_sequence_gc_content.png]

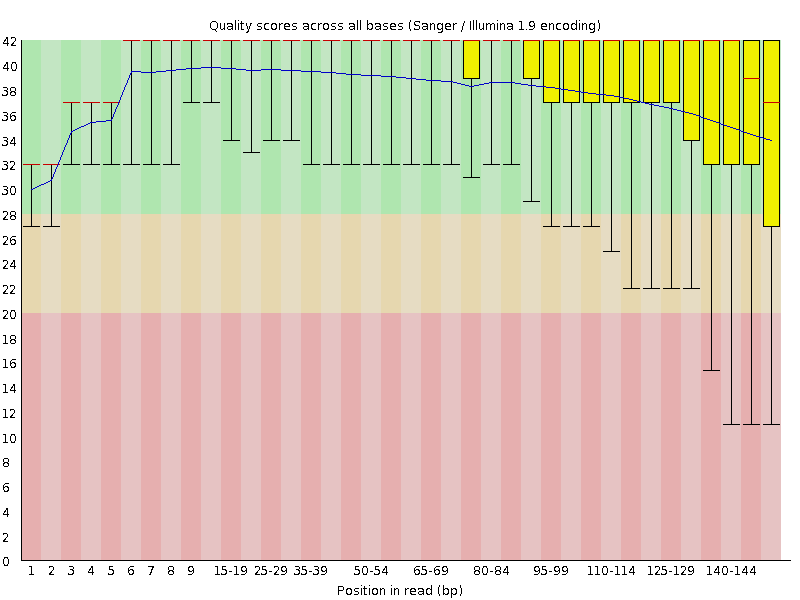

Supplement: Supplementary file 18 [file 953FileS3.zip › QC/A4-3_2.per_base_quality.png]

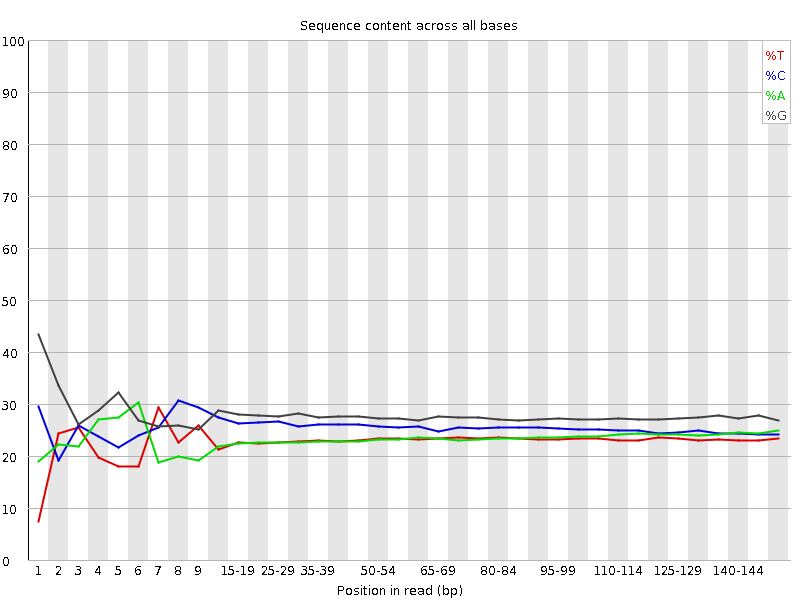

Supplement: Supplementary file 18 [file 953FileS3.zip › QC/A4-3_2.per_base_sequence_content.png]

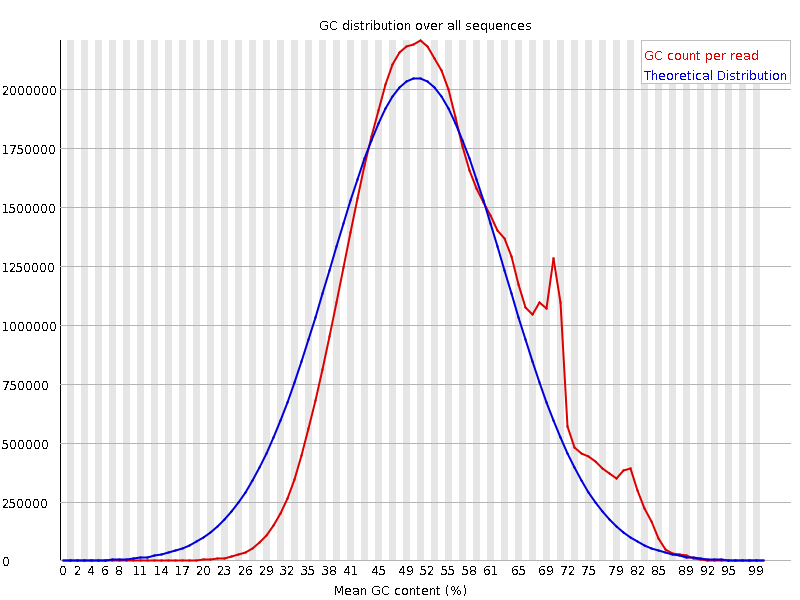

Supplement: Supplementary file 18 [file 953FileS3.zip › QC/A4-3_2.per_sequence_gc_content.png]

Classification of Raw Reads(A6-1)

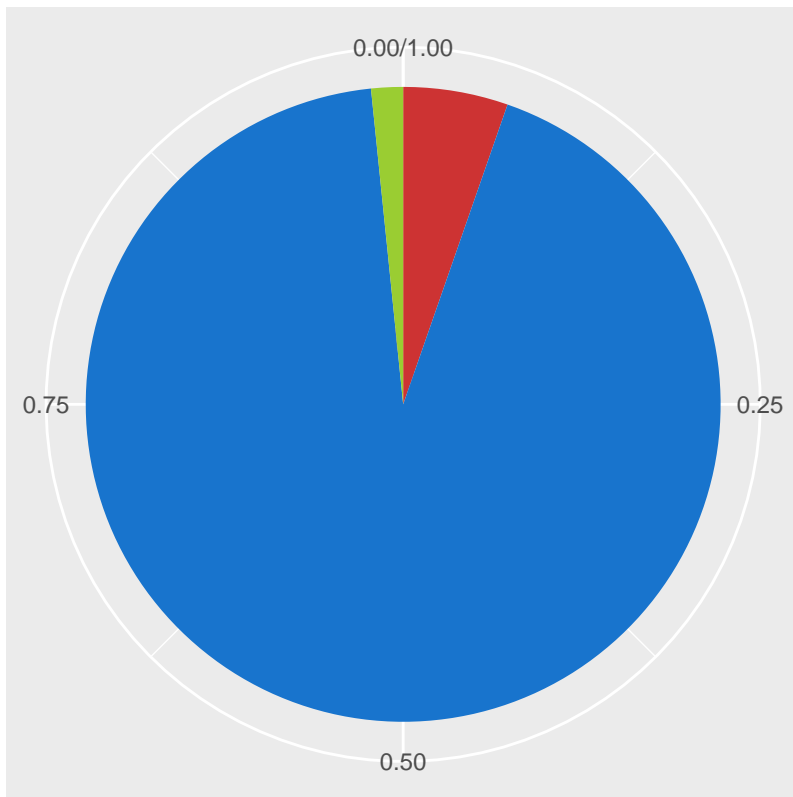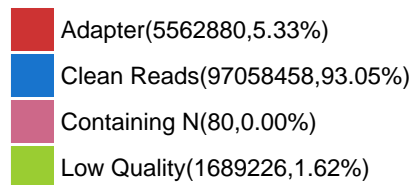

Supplement: Supplementary file 18 [file 953FileS3.zip › QC/A6-1.qc.pdf]

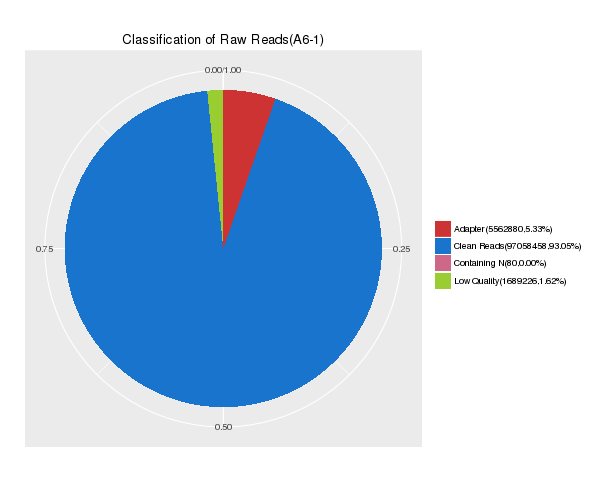

Supplement: Supplementary file 18 [file 953FileS3.zip › QC/A6-1.qc.png]

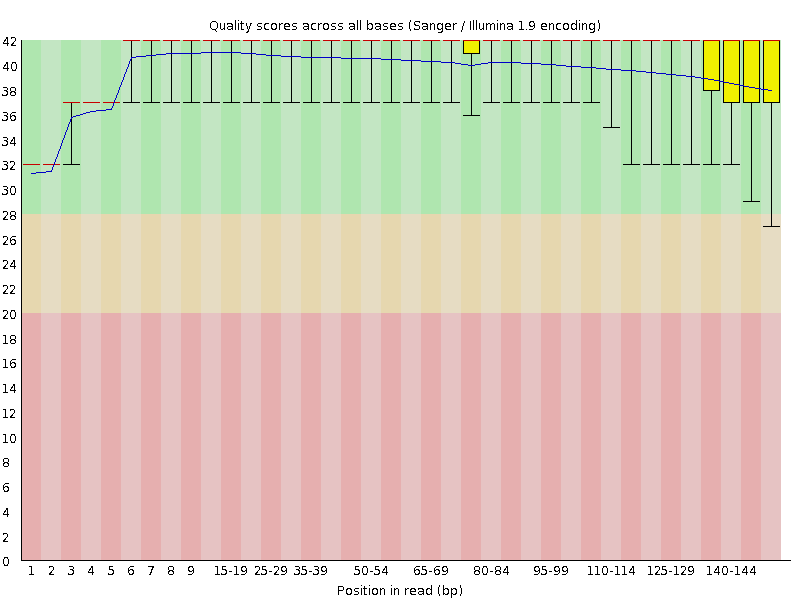

Supplement: Supplementary file 18 [file 953FileS3.zip › QC/A6-1_1.per_base_quality.png]

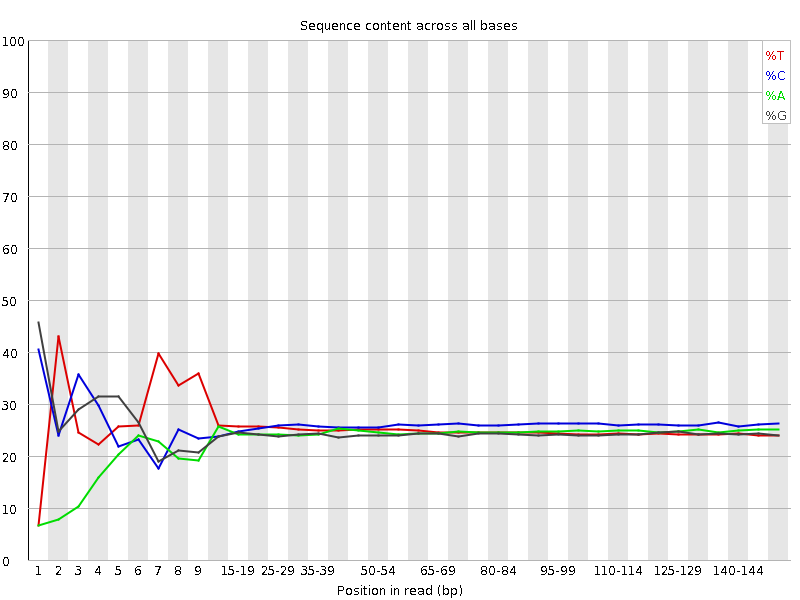

Supplement: Supplementary file 18 [file 953FileS3.zip › QC/A6-1_1.per_base_sequence_content.png]

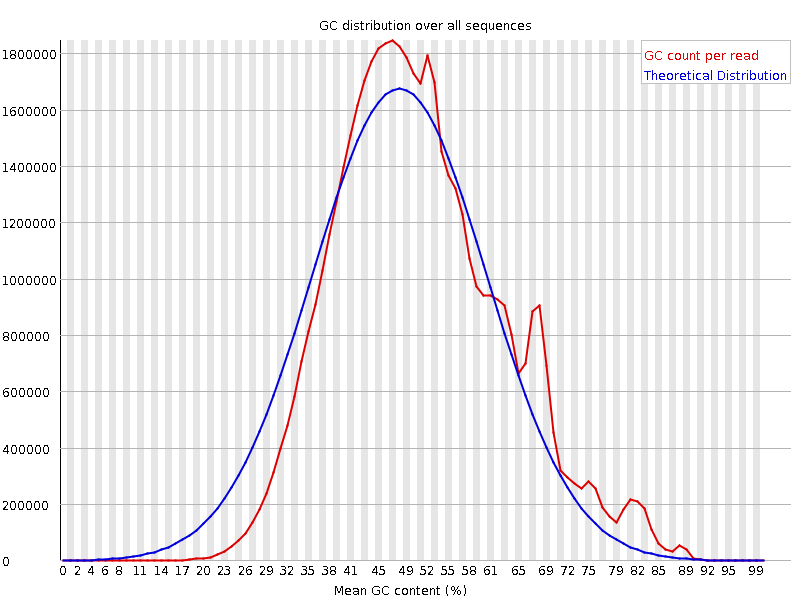

Supplement: Supplementary file 18 [file 953FileS3.zip › QC/A6-1_1.per_sequence_gc_content.png]

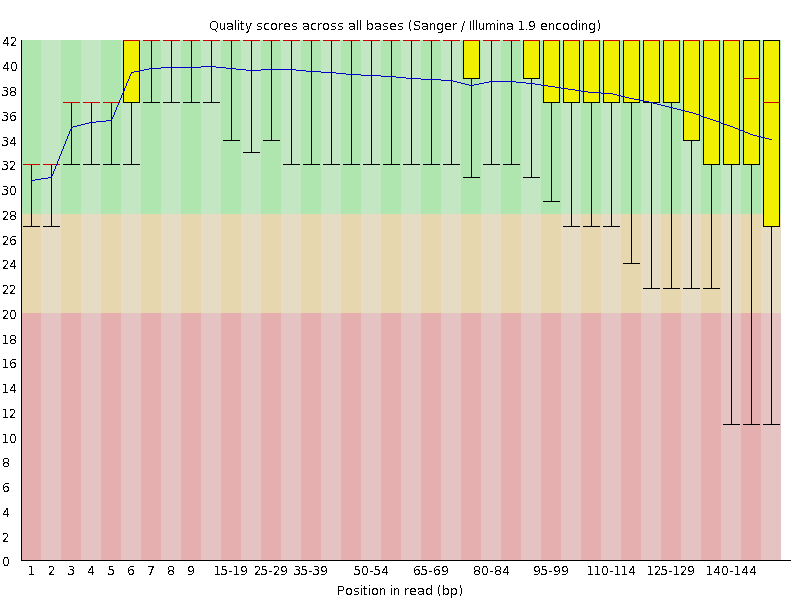

Supplement: Supplementary file 18 [file 953FileS3.zip › QC/A6-1_2.per_base_quality.png]

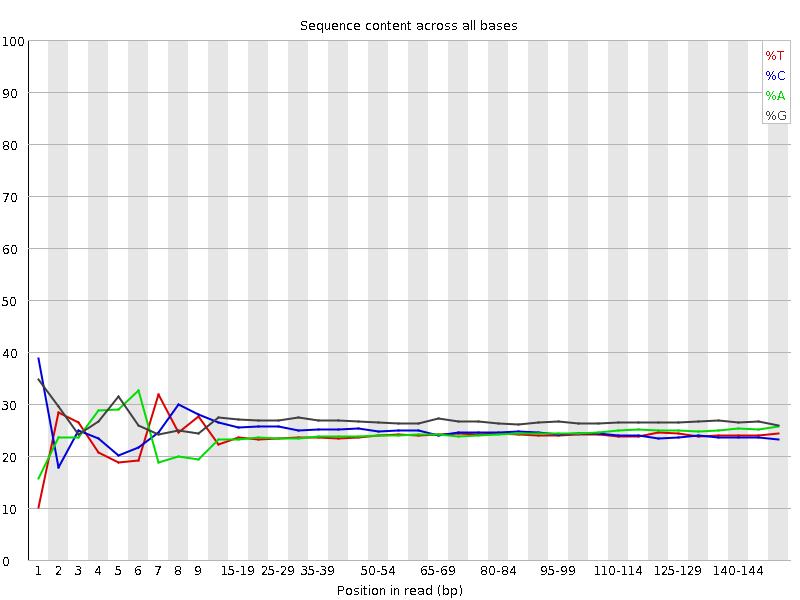

Supplement: Supplementary file 18 [file 953FileS3.zip › QC/A6-1_2.per_base_sequence_content.png]

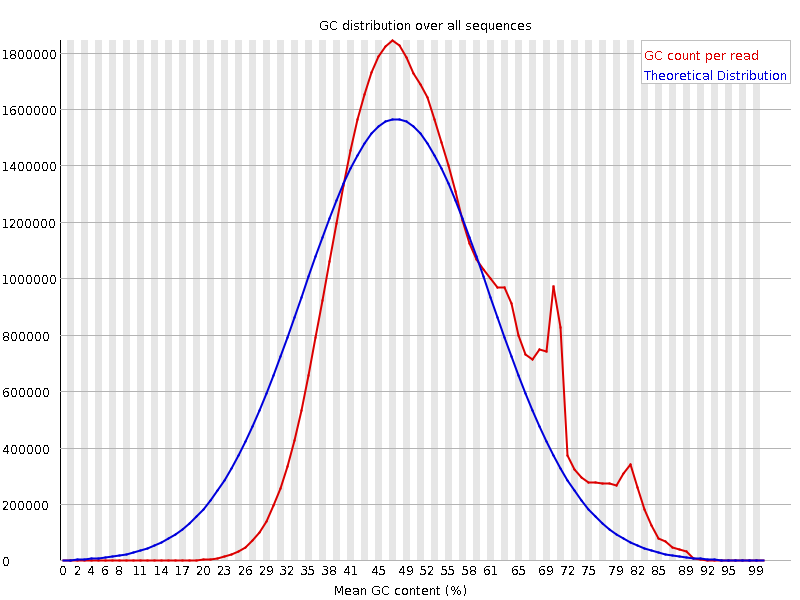

Supplement: Supplementary file 18 [file 953FileS3.zip › QC/A6-1_2.per_sequence_gc_content.png]

Classification of Raw Reads(A6-2)

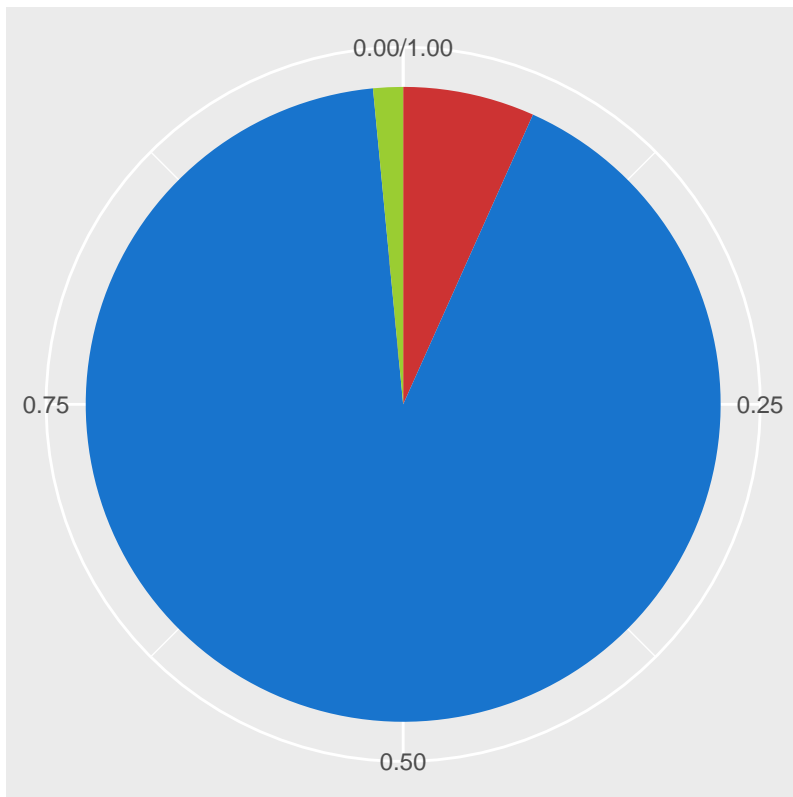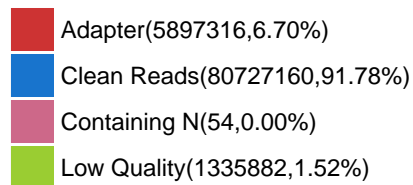

Supplement: Supplementary file 18 [file 953FileS3.zip › QC/A6-2.qc.pdf]

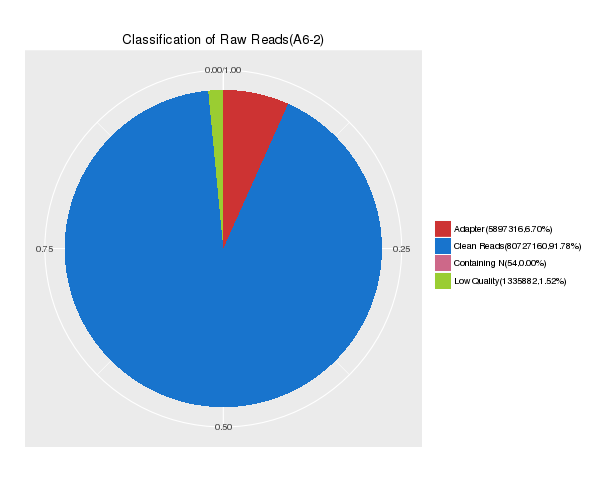

Supplement: Supplementary file 18 [file 953FileS3.zip › QC/A6-2.qc.png]

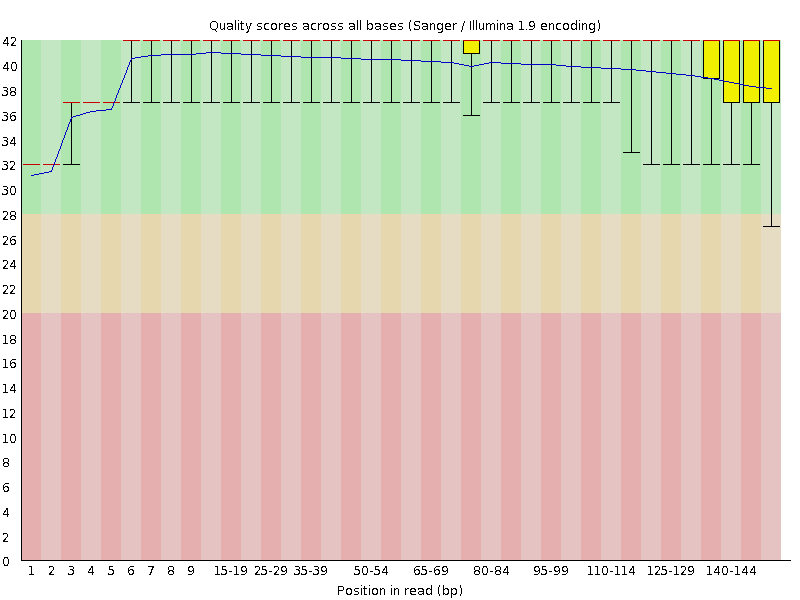

Supplement: Supplementary file 18 [file 953FileS3.zip › QC/A6-2_1.per_base_quality.png]

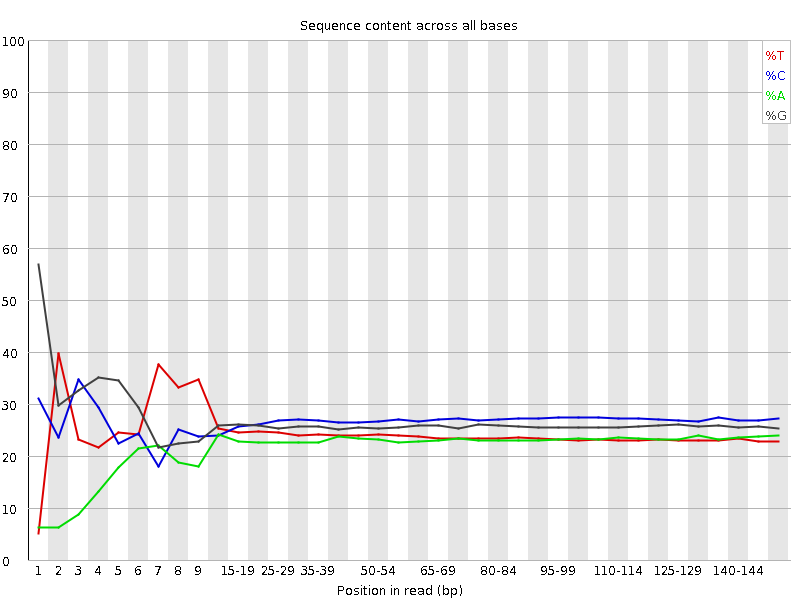

Supplement: Supplementary file 18 [file 953FileS3.zip › QC/A6-2_1.per_base_sequence_content.png]

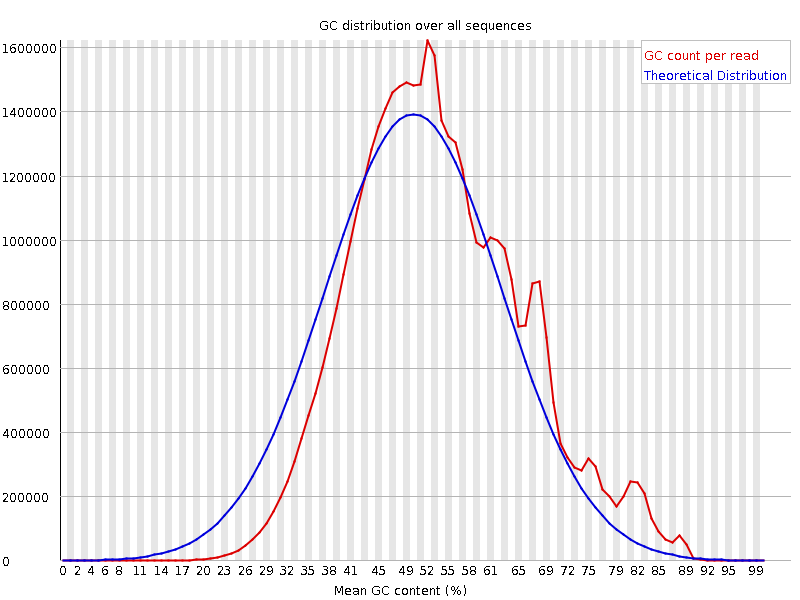

Supplement: Supplementary file 18 [file 953FileS3.zip › QC/A6-2_1.per_sequence_gc_content.png]

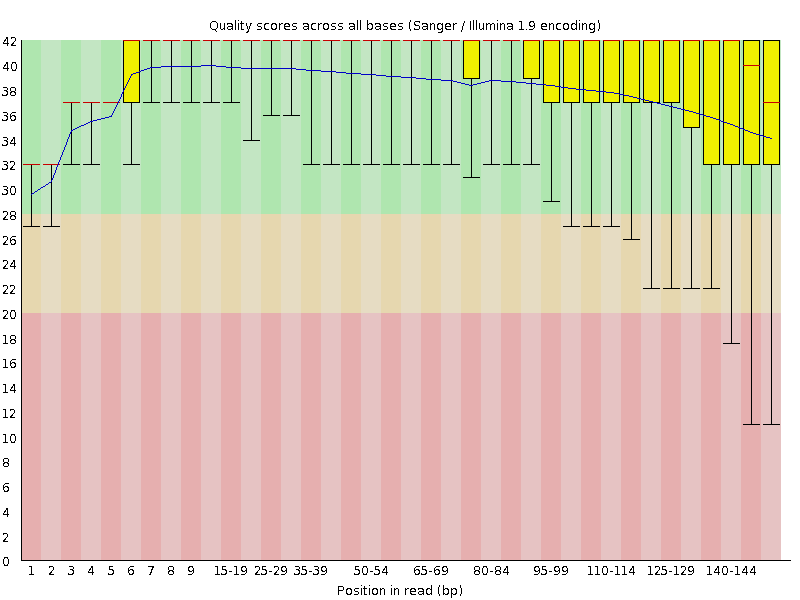

Supplement: Supplementary file 18 [file 953FileS3.zip › QC/A6-2_2.per_base_quality.png]

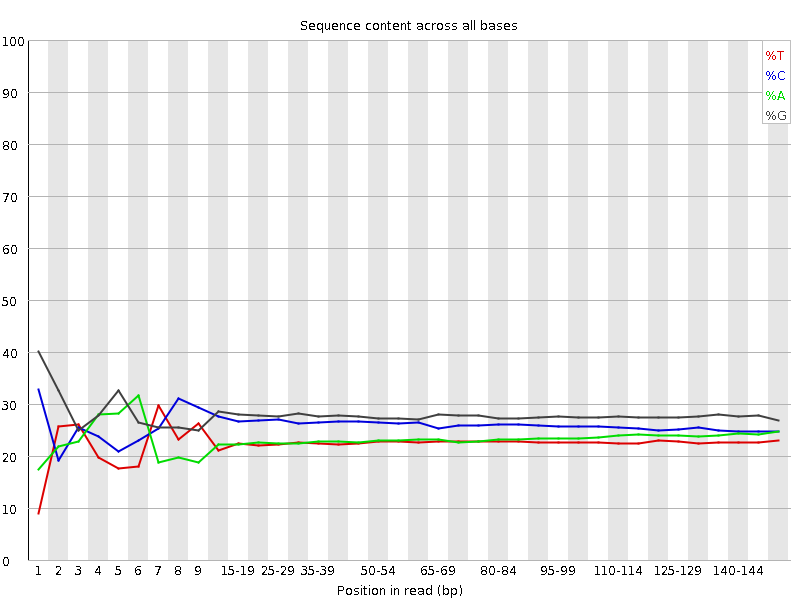

Supplement: Supplementary file 18 [file 953FileS3.zip › QC/A6-2_2.per_base_sequence_content.png]

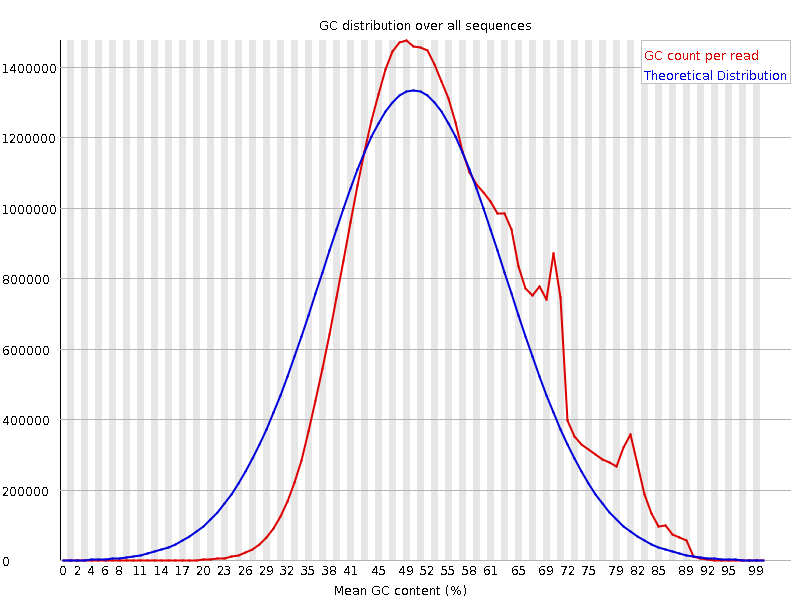

Supplement: Supplementary file 18 [file 953FileS3.zip › QC/A6-2_2.per_sequence_gc_content.png]

Classification of Raw Reads(A6-3)

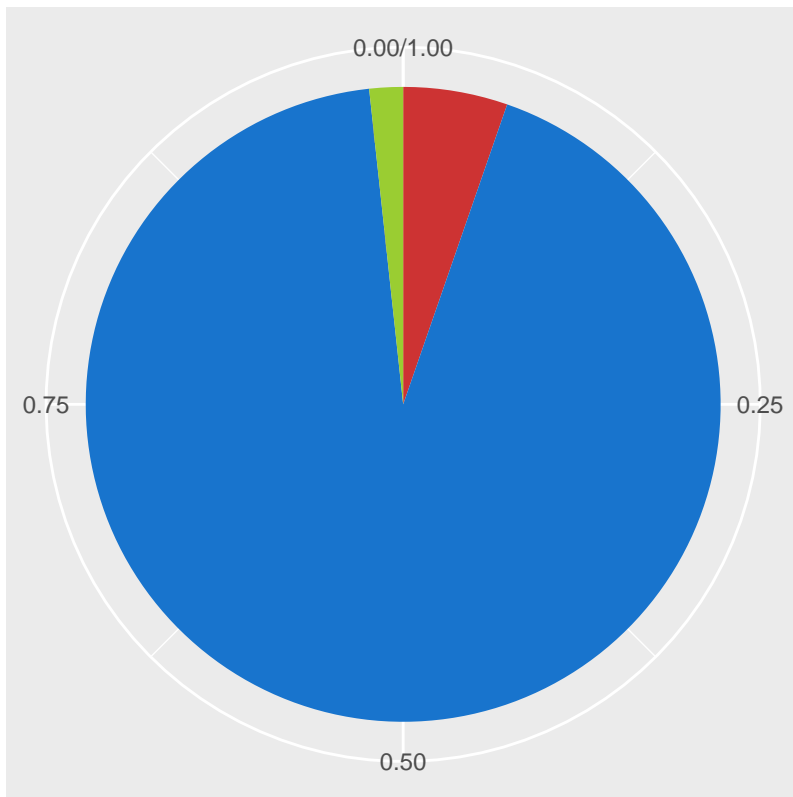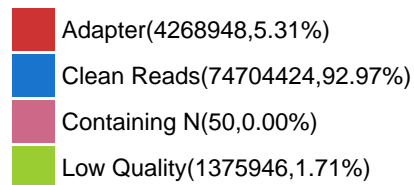

Supplement: Supplementary file 18 [file 953FileS3.zip › QC/A6-3.qc.pdf]

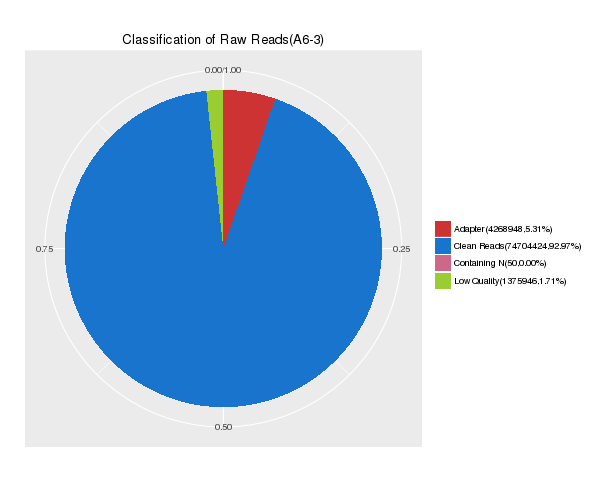

Supplement: Supplementary file 18 [file 953FileS3.zip › QC/A6-3.qc.png]

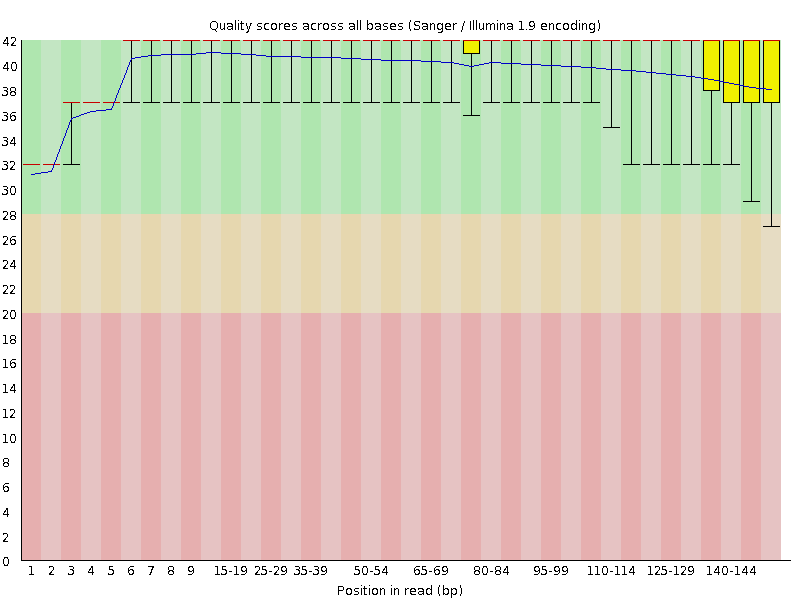

Supplement: Supplementary file 18 [file 953FileS3.zip › QC/A6-3_1.per_base_quality.png]

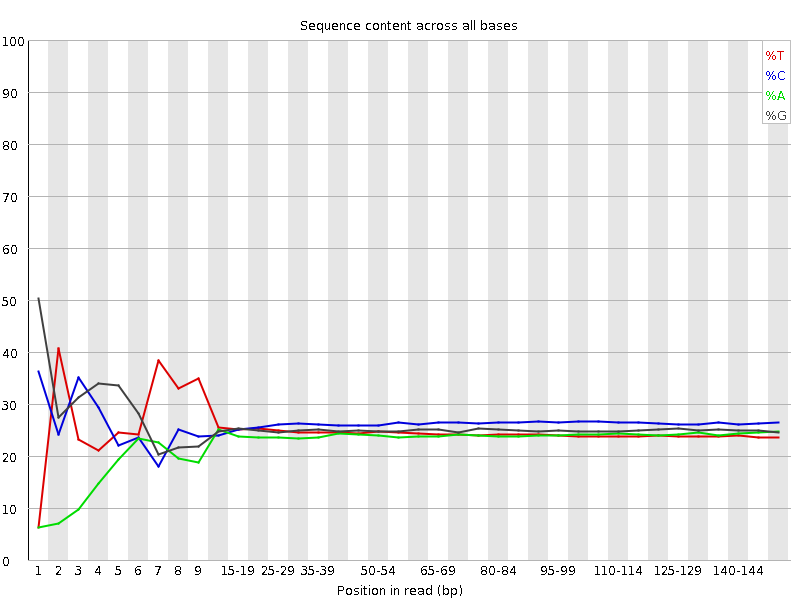

Supplement: Supplementary file 18 [file 953FileS3.zip › QC/A6-3_1.per_base_sequence_content.png]

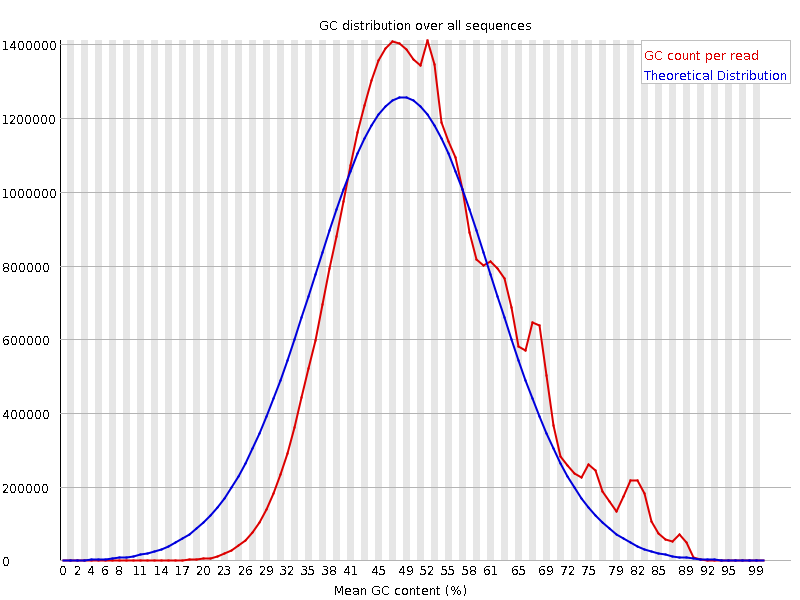

Supplement: Supplementary file 18 [file 953FileS3.zip › QC/A6-3_1.per_sequence_gc_content.png]

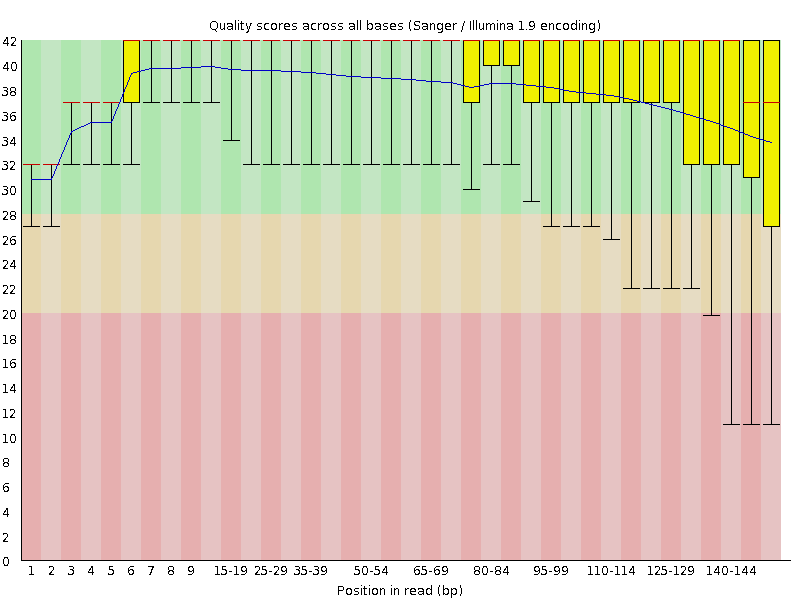

Supplement: Supplementary file 18 [file 953FileS3.zip › QC/A6-3_2.per_base_quality.png]

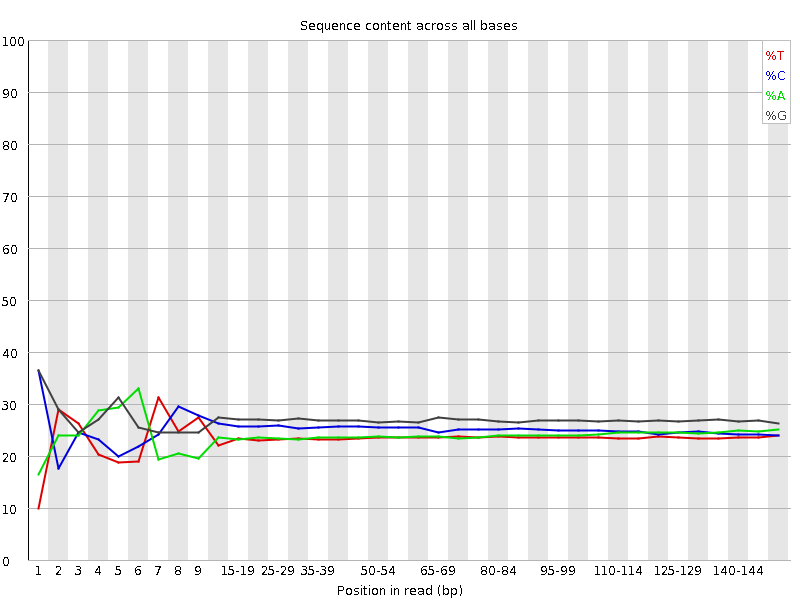

Supplement: Supplementary file 18 [file 953FileS3.zip › QC/A6-3_2.per_base_sequence_content.png]

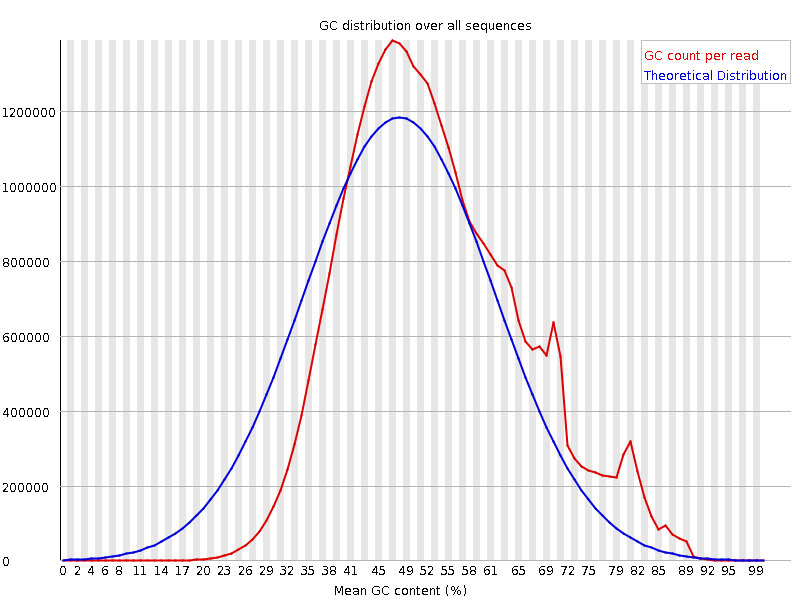

Supplement: Supplementary file 18 [file 953FileS3.zip › QC/A6-3_2.per_sequence_gc_content.png]
